# Polyphenols from the Peels of *Punica granatum* L. and Their Bioactivity of Suppressing Lipopolysaccharide-Stimulated Inflammatory Cytokines and Mediators in RAW 264.7 Cells via Activating p38 MAPK and NF-κB Signaling Pathways

**DOI:** 10.3390/molecules27144622

**Published:** 2022-07-20

**Authors:** Hui-Min Li, Ongher Kouye, Ding-Shan Yang, Ya-Qi Zhang, Jing-Ya Ruan, Li-Feng Han, Yi Zhang, Tao Wang

**Affiliations:** 1 State Key Laboratory of Component-Based Chinese Medicine, Tianjin University of Traditional Chinese Medicine, 10 Poyanghu Road, West Area, Tuanbo New Town, Jinghai District, Tianjin 301617, China; 15380711687@163.com (H.-M.L.); kouye2022@163.com (O.K.); ruanjingya@tjutcm.edu.cn (J.-Y.R.); hanlifeng_1@sohu.com (L.-F.H.); 2 Tianjin Key Laboratory of TCM Chemistry and Analysis, Tianjin University of Traditional Chinese Medicine, 10 Poyanghu Road, West Area, Tuanbo New Town, Jinghai District, Tianjin 301617, China; yangdingshan1996@163.com (D.-S.Y.); zhangyaq1997@163.com (Y.-Q.Z.)

**Keywords:** *Punica granatum* L., polyphenols, anti-inflammation, RAW 264.7 cells, p38 MAPK, NF-κB signaling pathway

## Abstract

*Punica granatum* L. (Punicaceae) is a popular fruit all over the world. Owning to its enriched polyphenols, *P. granatum* has been widely used in treating inflammation-related diseases, such as cardiovascular diseases and cancer. Twenty polyphenols, containing nine unreported ones, named punicagranins A–I (**1**–**9**), along with eleven known isolates (**10**–**20**), were obtained from the peels. Their detailed structures were elucidated based on UV, IR, NMR, MS, optical rotation, ECD analyses and chemical evidence. The potential anti-inflammatory activities of all polyphenols were examined on a lipopolysaccharide (LPS)-induced inflammatory macrophages model, which indicated that enhancing nitric oxide (NO) production in response to inflammation stimulated in RAW 264.7 cells was controlled by compounds **1**, **3**, **5**–**8**, **10**, **11**, **14** and **16**–**20** in a concentration-dependent manner. The investigation of structure–activity relationships for tannins **6**–**8** and **12**–**20** suggested that HHDP, flavogallonyl and/or gallagyl were key groups for NO production inhibitory activity. Western blotting indicated that compounds **6**–**8** could down-regulate the phosphorylation levels of proteins p38 MAPK, IKKα/β, IκBα and NF-κB p65 as well as inhibit the levels of inflammation-related cytokines and mediators, such as IL-6, TNF-α, iNOS and COX-2, at the concentration of 30 μM. In conclusion, polyphenols are proposed to be the potential anti-inflammatory active ingredients in *P. granatum* peels, and their molecular mechanism is likely related to the regulation of the p38 MAPK and NF-κB signaling pathways.

## 1. Introduction

Though moderate inflammation is a protective immune response against micro-organism invasion or damage, a pathological inflammation is the product of an uncontrolled of immune balance leading to chronic diseases, including inflammatory bowel disease, cardiovascular diseases, allergy, type 2 diabetes or even some kinds of cancer [1]. In the view of over-expression of pro-inflammatory cytokines and mediators lead to inflammatory response, this is a useful way to suppress their productions to avoid inflammation.

On the other hand, the pro-inflammatory cytokines and mediators are regulated by a large amount of signaling pathways, which indicates that selectively inhibiting the key target of corresponding signaling pathway may be a promising therapeutic strategy [2]. Macrophages, distributing throughout the body, can be activated by external stimuli, such as lipopolysaccharides (LPS—a major component of the outer membrane of Gram-negative bacteria). 

As an endotoxin, LPS can activate the nuclear transcription factor kappa-B (NF-κB) pathway through LPS-binding protein (LBP), myeloid differentiation receptor (CD14) and Toll-like receptor 4 (TLR4) receptors, promote the release of inflammatory factors and induce a series of inflammatory reactions [3]. They will be recruited to inflammatory sites and will release cascades of inflammatory molecules, thereby, producing a variety of inflammatory cytokines, including interleukin (IL)-6 and tumor necrosis factor (TNF)-α, and inflammatory mediators, such as nitric oxide (NO), prostaglandin E2 (PGE2) and inducible cyclooxygenase-2 (COX-2), leading to severe damage at the inflamed sites by amplifying the inflammation response [4].

In recent years, greater attention has been paid to developing natural functional compounds or herbal plant extracts to prevent and treat inflammatory disorders or, at least, relieve its symptoms [5]. Polyphenols are usually found in vegetables, fruits, beverages cereals, etc. The long-term intake of diets rich in polyphenols can protect against gastrointestinal tract, arthritis, cancer and cardiovascular disease because of their anti-inflammatory properties to [2,6].

*Punica granatum* L. belongs to the Punicaceae family, is originated from central Asia and is now widely planted all around the world, such as India, Iran, America and the Mediterranean regions due to its strong adaptability to different climates and soil conditions [7]. *P. granatum*, famous for super-fruit, has been widely used for centuries in folk medicine for the treatment of various inflammation-related diseases, including cardiovascular diseases and cancer, owning to a high content in polyphenols, particularly ellagitannins [2,7]. 

The nonedible part, namely the peels, is an excellent source of polyphenols that are more abundant in specific medicinal and nutritional value compared with the edible part of the fruit [8], which was reported to have anti-oxidant, anti-inflammatory, anti-hyperglycemia; anti-hypertension, reduce body weight, anti-cancer and other activities [2,9,10,11]. However, its effective substances are not totally clear, and little is known about the anti-inflammatory mechanism.

Therefore, the phytochemistry investigation of polyphenols from *P. granatum* peels was developed. An LPS-treated RAW 264.7 macrophages model was established to evaluate the anti-inflammatory activity of polyphenols by measuring the NO level. Furthermore, the signaling pathways of compounds exerted anti-inflammatory effect were also studied through a western blot assay.

## 2. Results and Discussion

### 2.1. Structural Characterization

**Figure 1:**
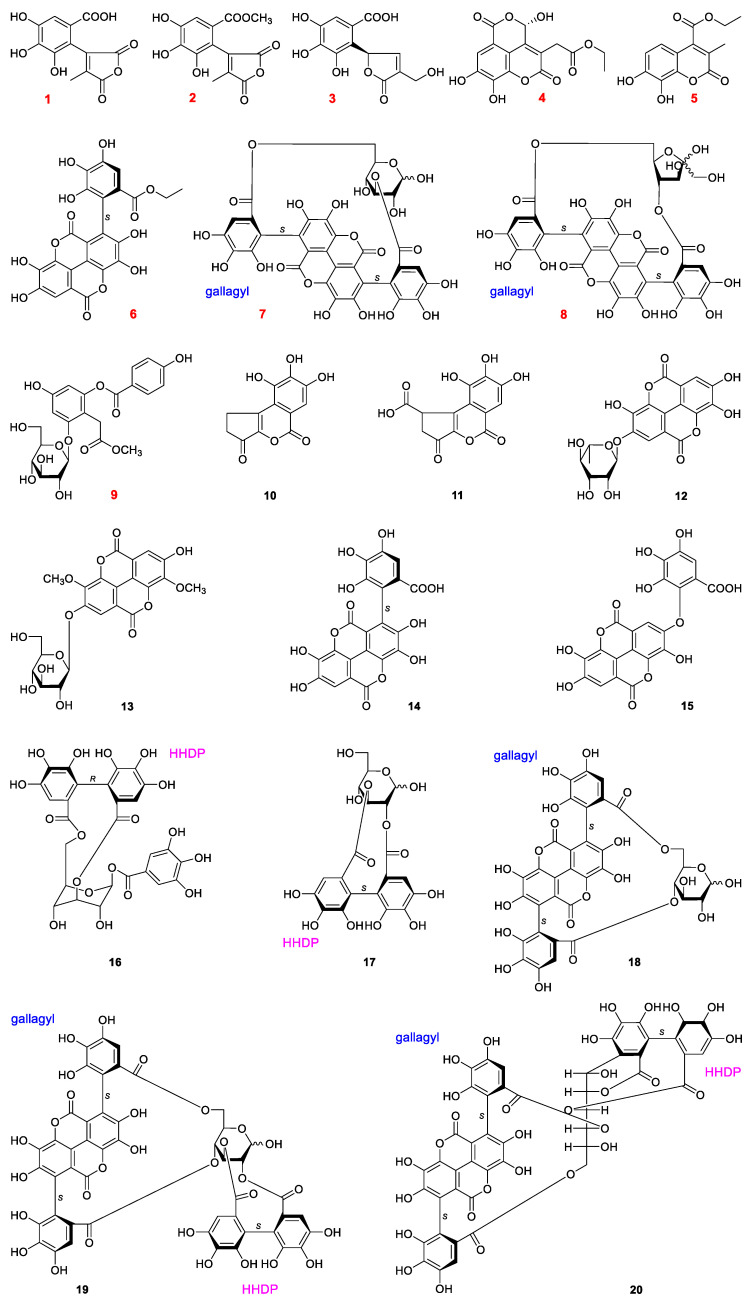
Structures of polyphenols obtained from the peels of *P. granatum*.

The 70% ethanol extraction of *P. granatum* peels were fractionated with various of materials for chromatographic separation, including D101 resin, silica gel, ODS and Sephadex LH-20 column chromatographies (CC). Then, the obtained fractions were purified by preparative high-performance liquid chromatography (pHPLC) to obtain polyphenols. The structures of gained polyphenols were identified by using kinds of spectroscopic methods, such as UV, IR, NMR, [α]_D_, MS, as well as electronic circular dichroism (ECD) spectra. The results were nine unreported polyphenols, named punicagranins A–I (**1**–**9**), together with eleven known isolates, brevifolin (**10**) [12], brevifolincarboxylic acid (**11**) [13], 4-*O*-*α*-l-rhamnopyranosyl ellagic acid (**12**) [14], 4-*O*-*β*-d-glucopyranosyl-3,3′-di-*O*-methylellagic acid (**13**) [15], (*S*)-flavogallonic acid (**14**) [16], valoneic acid dilactone (**15**) [17], corilagin (**16**) [18], 2,3-(*S*)-hexahydroxydiphenoyl-d-glucose (**17**) [19], punicalin (**18**) [20], punicalagin (**19**) [20] and punicacortein C (**20**) [21] (Figure 1) were obtained and identified.

Punicagranin A (**1**) was obtained as a white powder with molecular formula, C_12_H_8_O_8_ established on negative-ion HRESIMS analysis. Its IR spectrum exhibited characteristic absorptions accounting for hydroxyl (3171 cm^−1^), carbonyl (1790, 1715 cm^−1^) and aromatic ring (1608, 1551 cm^−1^). The ^1^H, ^13^C NMR (DMSO-*d*_6_) and HSQC spectra indicated the existences of one methyl [δ 2.14 (3H, s, H_3_-6)], one aromatic proton [δ 7.17 (1H, s, H-5′)] and ten quaternary carbons.

**Figure 2:**
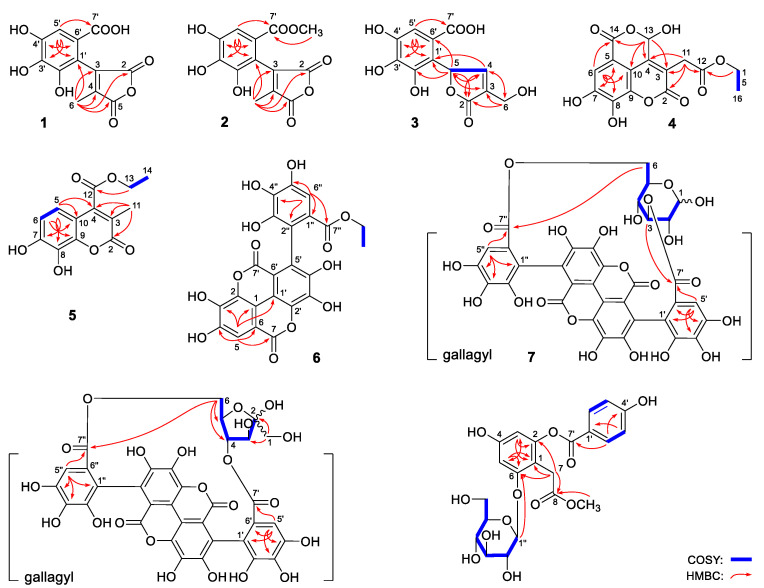
The main ^1^H ^1^H COSY and HMBC correlations of **1**–**9**.

The presence of gallic acid fragment was speculated by the correlations from δ_H_ 7.17 (H-5′) to δ_C_ 108.0 (C-1′), 121.5 (C-6′), 134.5 (C-3′), 146.4 (C-4′), 167.7 (C-7′). In addition, the cross peaks from δ_H_ 2.14 (H_3_-6) to δ_C_ 108.0 (C-1′), 119.7 (C-4), 142.2 (C-3), 160.8 (C-5), 166.3 (C-2) were also observed in its HMBC spectrum (Figure 2). Consequently, the structure of punicagranin A (**1**) was elucidated to be 3-(2,3,4-trihydroxy-4-carboxyphenyl)-4-methylfuran-2,5-dione.

The HRESIMS determining result revealed the molecular formula of punicagranin B (**2**) was C_13_H_10_O_8_. Comparing its ^1^H and ^13^C NMR with compound **1**, it was found that one more methoxyl [δ_H_ 3.70 (3H, s, 7′-COO*CH_3_*); δ_C_ 51.3 (7-COO*CH_3_*)] appeared in compound **2**. The ^13^C NMR signal of C-7′ was decreased 0.8 [δ_C_ 167.7 (C-7′) for **1**; 166.9 (C-7′) for **2**]. In combination with the correlation from δ_H_ 3.70 (3H, s, 7′-COO*CH_3_*) to δ_C_ 166.9 (C-7′) (Figure 2), punicagranin B (**2**) was deduced to be punicagranin A 7′-methyl ester.

Punicagranin C (**3**) was obtained as a white powder with negative optical rotation ([α]_D_^25^ −7.0). HRESIMS spectrum revealed its molecular formula was C_12_H_10_O_8_. The ^1^H NMR signal at δ 6.21 (1H, br. s, H-5), 7.79 (1H, br. s, H-4) and ^13^C NMR signal at δ 71.6 (C-5), 127.4 (C-3), 153.3 (C-4), 171.2 (C-2), along with the HMBC correlations from δ_H_ 7.79 (H-4) to δ_C_ 71.6 (C-5), 171.2 (C-2); δ_H_ 6.21 (H-5) to δ_C_ 127.4 (C-3), 171.2 (C-2) confirmed the presence of α,β-unsaturated-γ-lactone. It was similar to compound **1**, ^1^H, ^13^C NMR and 2D NMR spectra (Figure 2) suggested one 2-substituted gallic acid was present in compound **3**. Moreover, the ^1^H NMR spectrum suggested the existence of one hydroxymethyl [δ 4.94, 5.02 (1H each, both d, *J* = 19.0 Hz, H_2_-6)].

Then, α,β-unsaturated-γ-lactone, 2-substituted gallic acid and hydroxymethyl were grouped together according to the correlations from δ_H_ 6.21 (H-5) to δ_C_ 115.6 (C-6′), 125.8 (C-1′), 139.9 (C-2′); δ_H_ 4.94, 5.01 (H_2_-6) to δ_C_ 127.4 (C-3), 153.3 (C-4), 171.2 (C-2). Finally, the absolute configuration of 5*R* was clarified by the positive Cotton effect at 223 nm displayed in its ECD spectrum [22].

Punicagranin D (**4**) was a pale yellow powder with molecular formula of C_15_H_12_O_9_ deduced from HRESIMS analysis. The thin layer chromatography (TLC) of it showed blue-purple fluorescent spots under the UV lamp of 365 nm before and after spraying with 10% aqueous H_2_SO_4_-EtOH. Additionally, its UV spectrum displayed the maximum absorption peaks at 274 and 344 nm. Then, punicagranin D (**4**) was speculated to be a coumarin.

Its ^1^H, ^13^C NMR and HSQC spectra suggested the presence of one ethyoxyl [δ 1.18 (3H, t, *J* = 7.0 Hz, H_3_-16), 4.09 (2H, q, *J* = 7.0 Hz, H_2_-15)], one methylene [δ 3.63 (2H, s, H_2_-11)], one oxygenated methine [δ 6.64 (1H, s, H-13)] and ten quaternary carbons. The long-range correlations from δ_H_ 7.42 (1H, s, H-6) to δ_C_ 109.6 (C-10), 109.8 (C-5), 139.6 (C-8), 149.7 (C-7), 161.2 (C-14) indicated the presence of galloyl.

Its planar structure was clarified by the cross peaks from δ_H_ 3.63 (H_2_-11) to δ_C_ 116.6 (C-3), 141.8 (C-4), 160.7 (C-2), 169.2 (C-12); δ_H_ 6.64 (1H, s, H-13) to δ_C_ 109.6 (C-10), 116.6 (C-3), 141.8 (C-4), 161.2 (C-14); δ_H_ 4.09 (H_2_-15) to δ_C_ 169.2 (C-12) observed in the HMBC spectrum (Figure 2). Its ECD spectrum displayed positive Cotton effects at 222 nm, which was similar to that of (*S*)-euphorhirtin H [23]. Then, its absolute configuration, 13*R* was revealed.

The molecular formula of punicagranin E (**5**) was determined to be C_13_H_12_O_6_ by HRESIMS. Its UV absorption (λ_max_: 266 and 328 nm) and the similarity phenomenon displayed in TLC indicated it was also a coumarin. Thirteen carbon signals appeared in its ^13^C NMR spectrum. Combining with its ^1^H NMR and HSQC spectra, the existence of two aromatic protons [δ 6.75, 6.84 (1H each, both d, *J* = 8.5 Hz, H-5 and 6)], one methyl [δ 2.01 (3H, s, H_3_-11)], one ethyoxyl [δ 1.35 (3H, t, *J* = 7.0 Hz, H_3_-14), 4.46 (2H, q, *J* = 7.0 Hz, H_2_-13)], two unsaturated carboxyl [δ_C_ 161.0 (C-2), 165.1 (C-12)], as well as six aromatic quaternary carbon were consolidated. In the HMBC spectrum, the correlations from δ_H_ 6.75 (H-5) to δ_C_ 108.7 (C-10), 142.3 (C-9), 142.5 (C-4), 149.0 (C-7); δ_H_ 6.84 (H-6) to δ_C_ 108.7 (C-10), 132.3 (C-8); δ_H_ 2.01 (H_3_-11) to δ_C_ 116.7 (C-3), 142.5 (C-4), 161.0 (C-2); δ_H_ 4.46 (H_2_-13) to δ_C_ 165.1 (C-12) were observed. Then, the structure of punicagranin E (**5**) was elucidated.

Punicagranin F (**6**) was measured on HRESIMS to give the molecular formular, C_23_H_24_O_13_. There was one ethyoxyl signals showed in its ^1^H NMR spectrum at δ_H_ 0.87 (3H, t, *J* = 7.0 Hz, 7″-OCH_2_C*H_3_*), 3.86 (2H, q, *J* = 7.0 Hz, 7″-OC*H_2_*CH_3_) and in its ^13^C NMR spectrum at δ_C_ 13.6 (7″-OCH_2_*C*H_3_), 59.2 (7″-O*C*H_2_CH_3_). Twenty-three carbon signals were observed in its ^13^C NMR spectrum, and twenty-one of them were in the field of 107–166 ppm. In particularly, carbonyl signal at δ_C_ 157.4 (C-7′), 159.2 (C-7), 165.9 (C-7″) suggested the presence of three gallic acid moieties. However, there were only two aromatic protons [δ_H_ 7.11 (1H, s, H-6″), 7.50 (1H, s, H-5)] displayed in its ^1^H NMR spectrum.

The above-mentioned information indicated that punicagranin F (**6**) was a tannin formed by the condensation of three molecules of gallic acid. The long-range correlations from δ_H_ 7.50 (1H, s, H-5) to δ_C_ 107.1 (C-6), 112.6 (C-1), 139.4 (C-3), 147.7 (C-4), 159.2 (C-7); δ_H_ 7.11 (1H, s, H-6″) to δ_C_ 117.9 (C-2″), 119.2 (C-6′), 125.3 (C-1″), 137.2 (C-4″), 143.9 (C-5″), 165.9 (C-7″), along with the comparison of its spectroscopic data with those of compound **14**, the presence of flavogallonyl moiety was clarified. Moreover, according to the cross peak from δ_H_ 3.86 (7″-OC*H*_2_CH_3_) to δ_C_ 165.9 (C-7″), the substituted position of ethyoxyl was consolidated.

Finally, its Cotton effects displayed at 326 nm (positive), 298 nm (negative) and 255 nm (negative) were consistent with those of methyl (*S*)-heptamethylflavogallonate [24], suggesting the chirality of the flavogallonyl group was in the *S*-series. Moreover, its opposite optical rotation [([α]_D_^25^ −15.0, in MeOH)] with that of (*R*)-flavogallonic acid [([α]_D_^25^ +20.3, in MeOH)] [25] further clarified the conclusion. Then, the structure of punicagranin F (**6**) was elucidated to be ethyl (*S*)-flavogallonate. Though the plannar structure of it had been speculated by Abiodun et al. through LC-MS analysis [26], the absolute configuration of it was expounded here for the first time.

Punicagranin G (**7**) was obtained as yellow powder with negative optical rotation ([α]_D_^25^ −73.9, in H_2_O). Its molecular formular, C_34_H_22_O_22_, was established by HRESIMS. However, sixty-eight signals displayed in its ^13^C NMR spectrum. The ^1^H and ^13^C NMR spectra of **7** showed duplicated signals for the sugar and polyphenol moieties, indicating an equilibrium mixture of α and β anomeric forms with the ratio about 1:2 in solution. After hydrolysis with 1 M HCl, d-glucose was detected from its hydrolysate [27]. The cross-peaks between δ_H_ 2.46 (1H, dd, *J* = 3.6, 9.6 Hz, Glc-H-2α) and δ_H_ 4.81 (1H, d, *J* = 3.6 Hz, Glc-H-1α), 4.83 (1H, dd, *J* = 9.6, 10.0 Hz, Glc-H-3α); δ_H_ 1.75 (1H, dd, *J* = 10.0, 10.0 Hz, Glc-H-4α) and δ_H_ 3.55 (1H, m, Glc-H-5α), 4.83 (Glc-H-3α); δ_H_ 3.55 (Glc-H-5α) to δ_H_ [3.55 (1H, br. d, ca. *J* = 12 Hz), 4.67 (1H, dd, *J* = 3.2, 12.0 Hz), Glc-H_2_-6α] consolidated the α-d-glucopyranosyl.

The presence of β-d-glucopyranosyl was clarified by the correlations between δ_H_ 2.28 (1H, dd, *J* = 7.6, 9.2 Hz, Glc-H-2β) and δ_H_ 4.25 (1H, d, *J* = 7.6 Hz, Glc-H-1β), 4.57 (1H, dd, *J* = 9.2, 9.6 Hz, Glc-H-3β); δ_H_ 1.79 (1H, dd, *J* = 9.6, 9.6 Hz, Glc-H-4β) and δ_H_ 3.03 (1H, m, Glc-H-5β), 4.57 (Glc-H-3β); δ_H_ 3.03 (Glc-H-5β) and δ_H_ [3.53 (1H, br. d, ca. *J* = 12 Hz), 4.75 (1H, dd, *J* = 3.2, 12.4 Hz), Glc-H_2_-6β]. Except for the six carbon signals assigned to d-glucose, all of the twenty-eight duplicated carbon signals were in the low field.

The four pairs of carbonyl carbon signals at δ_C_ 160.0, 160.8 (lactone carbonyl-C-α), 169.1 (C-7′α), 170.7 (C-7″α); 159.9, 160.8 (lactone carbonyl-C-β), 169.0 (C-7′β), 170.7 (C-7″β) together with twenty-four duplicated aromatic carbon signals in the field of 108–161 ppm suggested the presence of four galloyl groups. The presence of (*S*,*S*)-gallagyl was confirmed by the comparison of its NMR data (in CD_3_OD), optical rotation ([α]_D_^25^ −73.9, in H_2_O) as well as ECD spectrum (Figure S50, Supplementary Materials) with those of punicalin (4,6-(*S*,*S*)-gallagyl-d-glucose (**18**) [20,28], whose ^1^H, ^13^NMR data, [α]_D_^25^ and ECD spectrum (Figure S69, Supplementary Materials) could be found in the supporting information.

Moreover, the correlations from δ_H_ 4.83 (Glc-H-3α) to δ_C_ 169.1 (C-7′α); δ_H_ 4.57 (Glc-H-3β) to δ_C_ 169.0 (C-7′β); δ_H_ 3.55, 4.67 (Glc-H_2_-6α) to δ_C_ 170.7 (C-7″α); δ_H_ 3.53, 4.75 (Glc-H_2_-6β) to δ_C_ 170.7 (C-7″β) indicated the two ester carbonyl groups in gallagyl were linked with C-3 and C-6 of d-glucosyl. Thus, the structure of punicagranin G (**7**) was determined to be 3,6-(*S*,*S*)-gallagyl-d-glucose.

Punicagranin H (**8**), [α]_D_^25^ −85.1 (in H_2_O). The HRESIMS revealed it had the same molecular formular, C_34_H_22_O_22_ as compound **7**. d-fructose was yielded from compound **8** after acid hydrolysis [27]. Its ^1^H and ^13^C NMR spectra suggested that it also contained (*S*,*S*)-gallagyl [δ_C_ 160.3, 160.5 (lactone carbonyl-C-β); 160.2, 160.4 (lactone carbonyl-C-α), 169.3, 169.7 (ester carbonyl-C-β); 169.4, 170.0 (ester carbonyl-C-α)].

d-glucosyl disappeared, and d-fructofuranosyl appeared in compound **8**, which was clarified by the cross peaks showed in the ^1^H ^1^H COSY spectrum, as well as the correlations from H_2_-1 to C-2, C-3 displayed in its HMBC spectrum (Figure 2). The correlations from H-4 to C-7′; H_2_-6 to C-7″ and the ECD similarity between it and compound **7** suggested the structure of punicagranin H (**8**) was 4,6-(*S*,*S*)-gallagyl-d-fructofuranose. It was a mixture of 4,6-(*S*,*S*)-gallagyl-α-d-fructofuranose and 4,6-(*S*,*S*)-gallagyl-β-d-fructofuranose with the ratio 1:3 nearly.

Punicagranin I (**9**) was obtained as a white powder. HRESIMS analysis showed its molecular formular was C_22_H_24_O_12_. Its IR spectrum displayed characteristic absorptions assigned to hydroxyl (3318 cm^−1^), α,β-unsaturated ketone (1718 cm^−1^), aromatic ring (1606, 1512, 1457 cm^−1^) and ether function (1076 cm^−1^). After hydrolyzing with 1 M HCl, the product of compound **9** was analyzed by HPLC combining with an optical rotation detector.

As a result, d-glucose was detected [27]. The ^1^H, ^13^C NMR and 2D NMR (^1^H ^1^H COSY, HSQC and HMBC) spectra indicated the existence of one AA′BB′ spin-coupled benzene ring [δ 6.90 (2H, d, *J* = 8.5 Hz, H-3′,5′), 7.98 (2H, d, *J* = 8.5 Hz, H-2′,6′)], one 1,2,3,5-tetra substituted benzene ring [δ 6.37, 6.65 (1H each, both d, *J* = 2.5 Hz, H-3 and 5)], one methylene [δ 3.60, 3.66 (1H each, both d, *J* = 16.5 Hz, H_2_-7)], two carboxyl [δ_C_ 166.0 (C-7′), 174.3 (C-8)], one methoxy group [δ 3.53 (3H, s, 8-COOC*H*_3_)] and one β-d-glucopyranosyl [δ 4.86 (1H, d, *J* = 7.5 Hz, H-1″)]. Finally, the long-range correlations were found from δ_H_ 3.60, 3.66 (H_2_-7) to δ_C_ 111.0 (C-1), 152.1 (C-2), 158.5 (C-6), 174.3 (C-8); δ_H_ 3.53 (8-COOC*H*_3_) to δ_C_ 174.3 (C-8); δ_H_ 7.98 (H-2′,6′) to δ_C_ 166.0 (C-7′); δ_H_ 4.86 (H-1″) to 158.5 (C-6) in its HMBC spectrum. Consequently, the structure of punicagranin I (**9**) was clarified.

The structures of the known compounds **10**–**20** were identified by comparing the spectroscopic data with those reported in literature. Among all of the obtained compounds, **6**–**8** and **12**–**20** were tannins.

To clarify whether compounds **2**, **4**–**6** and **9** were artificial products, a 70% ethanol extract and 70% methanol extract of *P. granatum* peels, together with the reference compounds of **2**, **4**–**6** and **9** were analyzed by LC-MS (Figure S65, Supplementary Materials). As compounds **6** and **9** could not be detected in the 70% MeOH and 70% EtOH extracts, respectively, they were elucidated to be artificial products. Compound **6** might be produced by refluxing with ethanol in the process of *P. granatum* peel extraction. Compound **9** might be the reaction product of the sample and methanol used as the mobile phase.

### 2.2. In Vitro Anti-Inflammatory Evaluation of Compounds ***1**–**20***

As an excess of NO can trigger severe damage at the inflamed sites by amplifying the inflammation response [4] and exploiting new strategies to treat the over production; thus, the release of NO is important and urgent. The LPS-induced RAW 264.7 macrophages model was established to evaluate the potential anti-inflammatory activities of the twenty polyphenols (**1**–**20**) by measuring the NO level as what has been reported [29].

In order to test the NO release inhibitory effects of all compounds at a safe concentration, a MTT assay was conducted first. The results showed that at the concentration of 30 μM, all compounds showed no cytotoxic on RAW 264.7 cell, except compound **19**, which showed no toxic until 15 μM (Figure S72, Supplementary Materials). Thus, in the in vitro anti-inflammatory assay, the concentration for compounds **1**–**18**, **20** and **19** were determined to be 30 μM and 15 μM, respectively. The results were compounds **1**, **3**, **5**–**8**, **10**, **11**, **14** and **16**–**20** displayed significant inhibitory effects on the NO release levels in LPS-activated RAW 264.7 cells (Table 1) at a non-toxic concentration.

**Figure 3:**
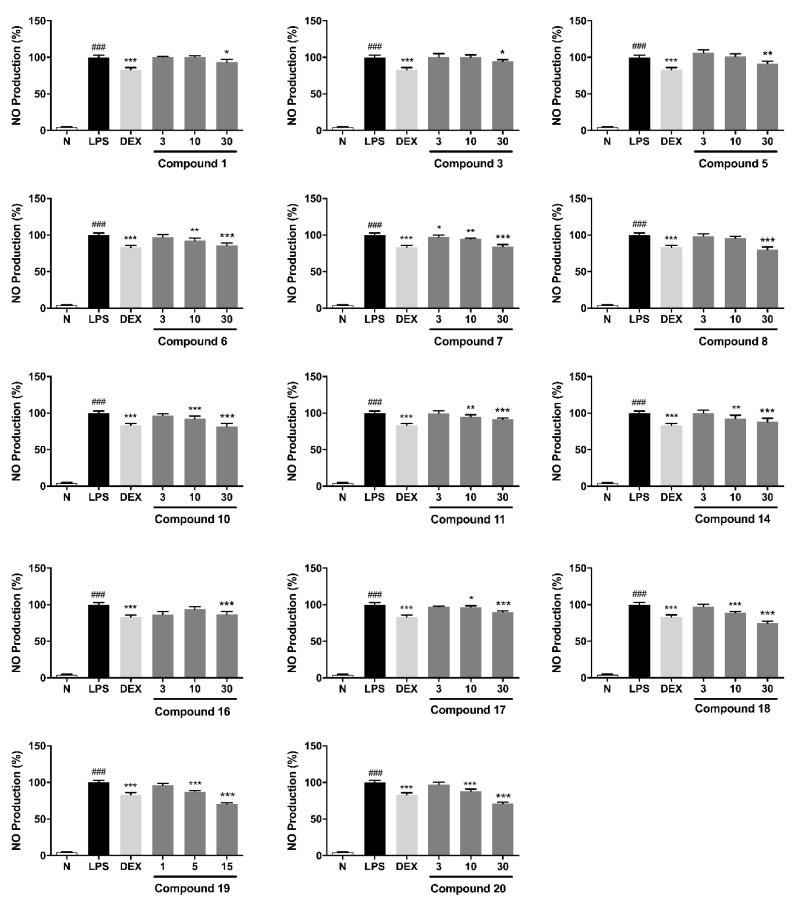
Concentration of compounds **1**, **3**, **5**–**8**, **10**, **11, 14** **and 16**–**20** on NO production in LPS-induced RAW 264.7 cells. N: normal group without LPS, DEX and tested samples; LPS: model group with 0.5 μg/mL LPS; DEX: positive drug group with 0.5 μg/mL LPS + 1.5 μg/mL DEX; tested compound groups were treated with 0.5 μg/mL LPS + compounds, and the final concentrations were 3, 10 and 30 μM for compounds **1**, **3**, **5**–**8**, **10**, **11, 14, 16**–**18** and **20** and 1, 5 and 15 μM for compound **19**, respectively. Values represent the mean ± SD of six determinations. * *p* < 0.05, ** *p* < 0.01, *** *p* < 0.001 (Differences between the compound-treated group and control group). ^###^ *p* < 0.001 (Differences between the control group and normal group).

It was found that compounds **1**, **3**, **5**–**8**, **10**, **11**, **14**, **16**–**18** and **20** reduced NO production in a concentration-dependent manner at 3, 10 and 30 μM, while compound **19** showed a similar mode at 1, 5 and 15 μM (Figure 3).

By comparing their bioactivities, the structure–activity relationships (SARs) can be summarized:

(1) Tannins containing gallagyl (**6**–**8** and **18**–**20**) had good inhibitory activity on NO release production. Compound **19** displayed stronger activity than **17**. Both of the results suggested gallagyl group may be a positive factor for NO inhibitory activity.
(2) The HHDP group in tannins also played an important role in inhibiting NO release (**18** vs. **19**).
(3) The activities of flavogallonic acid derivatives formed by the condensation of ellagic acid and gallic acid through carbon-carbon bonds were significantly stronger than those of valoneic acid derivatives produced by the condensation of ellagic acid and gallic acid through carbon-oxygen-carbon bond (**6** and **14** vs. **15**).
(4) Moreover, it was found that ethyl or methyl esterification of carboxyl group could affect the activity slightly only (**2** vs. **1** and **6** vs. **14**).
(5) Ellaganosides (**12** and **13**) showed no activity, which might be related to the introduction of sugar groups into ellagic acid (Table 1 and Figure 3).

This summary may provide more evidence for the structural modification of this type of polyphenols in the development of potential anti-inflammatory drugs.

### 2.3. Polyphenols from P. granatum Peels Exerted Anti-Inflammatory Effects through p38 MAPK and NF-κB Signaling Pathway

Moreover, the anti-inflammatory mechanism of compounds **6**–**8** at a concentration of 30 μM in LPS-induced RAW 264.7 macrophages model was investigated by western blot analyses. Comparing with the normal group, increased p38 mitogen-activated protein kinase (MAPK), IκB kinase (IKK)α/β, inhibitory nuclear factor κB-α (IκBα) and NF-κB p65 phosphorylation were observed in the model group.

**Figure 4:**
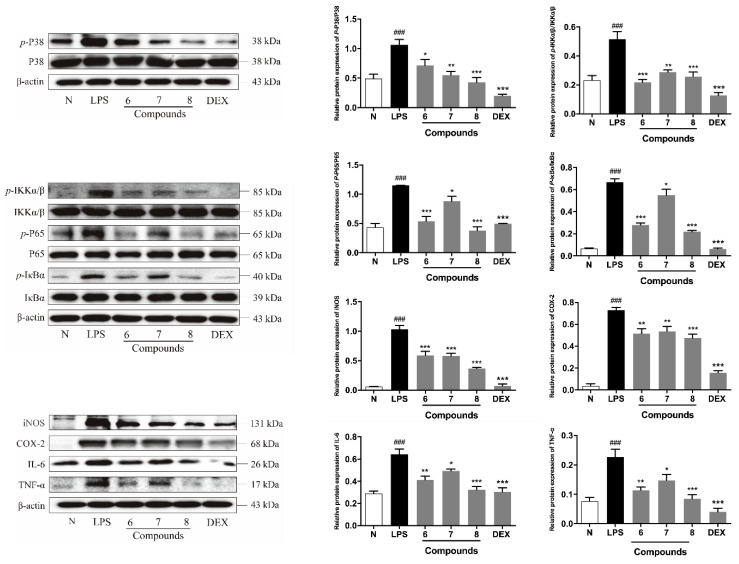
Inhibitory effects of compounds **6**–**8** on the protein expression of TNF-α, IL-6, iNOS, COX-2, IKK-α/β, *p*-IKK-α/β, IκBα, *p*-IκBα, NF-κB/p65, *p*-NF-κB/p65, p38 MAPK and *p*-p38 MAPK in RAW 264.7 cells. N: normal group without LPS, DEX and tested samples; LPS: model group with 0.5 μg/mL LPS; DEX: positive drug group with 0.5 μg/mL LPS + 1.5 μg/mL DEX; tested compound groups were treated with 0.5 μg/mL LPS + 30 μM compounds **6**–**8**, respectively. Values represent the mean ± SD of three determinations. * *p* < 0.05; ** *p* < 0.01; *** *p* < 0.001 (Differences between the compound-treated group and control group). ^###^ *p* < 0.001 (Differences between the LPS-treated group and control group).

Then, it was found that compounds **6**–**8** could down-regulate the phosphorylation levels of above-mentioned proteins. The expression levels of inflammation-related cytokines and mediators, such as IL-6, TNF-α, iNOS and COX-2 were down-regulated compared with the model group (Figure 4). The obtained findings also revealed that compound **8** owned the best anti-inflammatory activities, either in inhibiting NO production or iNOS expression, as well as down-regulating the IL-6 level in the LPS-treated RAW 264.7 cells.

p38 MAPK is one major member of MAPKs. As upstream modulators of inflammatory molecules, MAPKs have been considered to play critical role in the regulation of inflammatory mediators and cytokines, such as COX-2, inducible nitric oxide synthase (iNOS) in macrophage cells [30]. The p38 MAPK pathway, plays an important role in inflammation in particularly [31].

In addition, NF-κB, consists of subunits p50, p52, c-Rel, Rel A (p65) and Rel B, is normally interacted with IκBα proteins in the cytoplasm [32,33]. MAPKs, as one of the main regulatory factors of NF-κB, can up-regulate the expression of IKK, induce the phosphorylation and degradation of IκBα [34], thus, causing NF-κB p50 or p65 released to promote the transcription of pro-inflammatory genes, such as COX-2, iNOS, TNF-α, IL-6 and IL-1β [33,35,36]. Hence, p38 MAPK and NF-κB pathways have been regarded as potentially molecular targets for anti-inflammatory therapy.

According to the above-mentioned evidence, compounds **6**–**8** were confirmed to conduct anti-inflammatory activities by inhibiting p38 MAPK and NF-κB signaling pathways.

## 3. Conclusions

**Figure 5:**
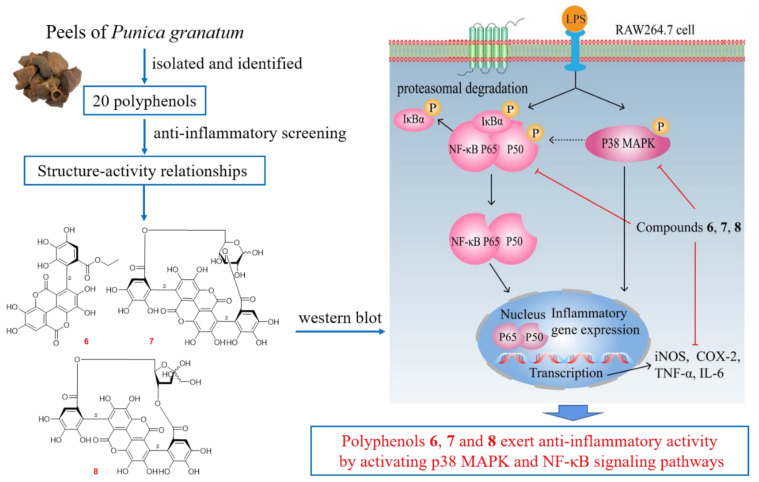
Summary graph of the investigation.

In summary, nine unreported polyphenols named punicagranins A–I (**1**–**9**), along with eleven known isolates (**10**–**20**) were gained from *P. granatum* peels. New compounds **6** and **9** were elucidated to be artificial products by LC-MS analysis. The potential anti-inflammatory activities of all the polyphenols were examined on an LPS-induced inflammatory macrophages model. It was found that compounds **1**, **3**, **5**–**8**, **10**, **11**, **14** and **16**–**20** reduced the LPS-induced secretion of NO in a concentration-dependent manner. The SARs analysis for tannins **6**–**8** and **12**–**20** suggested the introduction of HHDP, flavogallonyl and gallagyl played central roles in the NO inhibitory activity. Moreover, the new compounds **6**–**8** were found to suppress the expression of inflammatory cytokines and mediators, such as IL-6, TNF-α, iNOS and COX-2, through activating the p38 MAPK and NF-κB signaling pathways (Figure 5).

As described in the literature, corilagin (**16**) could restrict M1 macrophage activation by regulating the MAPK, NF-κB and interferon regulatory factor signaling pathways, resulting in a reduction in the expression of pro-inflammatory cytokines, such as IL-6, IL-12 and iNOS [37]. In addition, punicalin (**18**) reduced the release of inflammatory factors, including IL-1β and IL-18, via the ROS/NLRP3 pathway to exert anti-inflammatory function [38]. Punicalagin (**19**) could inhibit ERK/MAPK and JAK/SAT pathways in LPS-stimulated mouse primary peritoneal macrophages [39].

Combined with our investigation, polyphenols are proposed to be the potential anti-inflammatory active ingredients in *P. granatum* peels.

## 4. Experimental

### 4.1. Materials and Methods for Phytochemistry Research

#### 4.1.1. General Experimental Procedures

NMR spectra were determined on a Varian 400 MR spectrometer and/or 500 MHz NMR (Bruker BioSpin AG Industriestrasse 26 CH-8117, Fällanden, Switzerland) spectrometer with tetramethylsilane (TMS) as an internal standard. Negative-ion or positive-ion mode ESI-Q-Orbitrap MS was acquired using a Thermo ESI-Q-Orbitrap MS mass spectrometer connected to the UltiMate 3000 UHPLC instrument via ESI interface (Thermo, Waltham, MA, USA). Optical rotations, UV and IR spectra were determined on a Rudolph Autopol^®^ IV automatic polarimeter (l = 50 mm) (Rudolph Research Analytical, Hackettstown, NJ, USA), Varian Cary 50 UV-Vis (Varian, Inc., Hubbardston, MA, USA) and Varian 640-IR FT-IR spectrophotometer (Varian Australia Pty Ltd., Mulgrave, Australia), respectively.

CC were conducted on macroporous resin D101 (Haiguang Chemical Co., Ltd., Tianjin, China), silica gel (48–75 μm, Qingdao Haiyang Chemical Co., Ltd., Qingdao, China), ODS (40–75 µm, Chromatorex ODS MB, Fuji Silisia Chemical Co., Ltd., Tokyo, Japan) and Sephadex LH-20 (Ge Healthcare Bio-Sciences, Uppsala, Sweden). HPLC column: Cosmosil 5C_18_-MS-II column (4.6 mm i.d. × 250 mm, 5 µm, Nakalai Tesque, Inc., Tokyo, Japan) and Cosmosil 5C_18_-MS-II column (20 mm i.d. × 250 mm, 5 µm, Nakalai Tesque, Inc., Tokyo, Japan) were used to analysis and prepare the constituents, respectively. Precoated silica gel GF254 plates (10 cm × 20 cm, 0.25 mm thickness, Tianjin Silida Technology Co., Ltd., Tianjin, China) were used in TLC, and spots were detected under UV lights (254 and 365 nm) or by using the 10% sulfuric acid reagent.

Chloroform (CHCl_3_), methanol (MeOH), acetonitrile (CH_3_CN), and acetic acid (HAc) (chromatographically pure or analytical pure) were purchased from (Tianjin Concord Technology Co., Ltd., Tianjin, China).

#### 4.1.2. Plant Material

The medicinal herbs of the dried *P. granatum* peels were purchased from Beijing Tong ren Tang drug store, whose origin is from Anqing city, Anhui province, China. Then, the medicinal herbs were identified by Professor Lin Ma according to their characters (School of Chinese Materia Medica, Tianjin University of Traditional Chinese Medicine). The voucher specimen was deposited at the Academy of Traditional Chinese Medicine of Tianjin University of TCM.

#### 4.1.3. Extraction and Isolation

The dried peels of *P. granatum* (9.0 kg) were extracted three times with 70% EtOH under reflux for 3, 2 and 2 h, successively. Evaporation of the solvent under reduced pressure provided the 70% EtOH extract (2450.0 g). Then, an aliquot (1.63 kg) of it was dissolved in water and subjected to D101 resin CC (H_2_O → 95% EtOH) to obtain H_2_O (1034.0 g) and 95% EtOH (525.0 g) eluates, respectively.

The 95% EtOH eluate (PGE, 400.0 g) was subjected to silica gel CC [CHCl_3_-MeOH (100:5, *v*/*v*) → CHCl_3_-MeOH-H_2_O (10:3:1 → 7:3:1 → 6:4:1, *v*/*v*/*v*, lower layer) → MeOH] to give PGE 1–PGE 10. PGE 3 (1.2 g) was prepared by pHPLC [MeOH-H_2_O (30:70 → 40:60 → 55:45 → 60:40 → 100:0, *v*/*v*)] to obtain PGE 3-1–PGE 3-15. PGE 3-11 (52.7 mg) was purified by pHPLC [CH_3_CN-1% HAc (25:75, *v*/*v*)] to gain punicagranin E (**5**, 5.3 mg).

PGE 6 (13.6 g) was separated by ODS CC [MeOH-H_2_O (20:80 → 30:70 → 40:60 → 50:50 → 60:40 → 70:30 → 80:20 → 100:0, *v*/*v*)] and PGE 6-1–PGE 6-13 were given. PGE 6-2 (1.0 g) was isolated by Sephadex LH-20 CC (MeOH) and pHPLC [CH_3_CN-1% HAc (7:93, *v*/*v*)] to yield punicagranins A (**1**, 16.4 mg), B (**2**, 17.5 mg) and C (**3**, 284.8 mg). PGE 6-4 (2.7 g) was fractionated by Sephadex LH-20 CC (MeOH) to obtain PGE 6-4-1–PGE 6-4-13.

PGE 6-4-5 (260.1 mg) was purified by pHPLC [CH_3_CN-H_2_O (18:82, *v*/*v*)] and [MeOH-1% HAc (32:68, *v*/*v*)] to produce punicagranin D (**4**, 7.0 mg). PGE 6-4-10 (143.4 mg) was prepared by pHPLC [CH_3_CN-1% HAc (16:84, *v*/*v*)] to gain punicagranin F (**6**, 35.0 mg). PGE 7 (12.8 g) was subjected to Sephadex LH-20 CC (MeOH), then PGE 7-1–PGE 7-9 were given. PGE 7-4 (1.7 g) was separated by pHPLC [MeOH-H_2_O (20:80 → 30:70 → 40:60 → 50:50 → 100:0, *v*/*v*)] and pHPLC [CH_3_CN-1% HAc (16:84, *v*/*v*)] to give punicagranin H (**9**, 11.0 mg).

PGE 7-6 (1.1 g) was subjected to pHPLC [MeOH-H_2_O (30:70 → 40:60 → 50:50 → 60:40 → 100:0, *v*/*v*)] to get PGE 7-6-1–PGE 7-6-19. PGE 7-6-10 (63.1 mg) was purified by pHPLC [CH_3_CN-1% HAc (16:84, *v*/*v*)] to obtain brevifolin (**10**, 7.5 mg). PGE 7-7 (2.9 g) was isolated by pHPLC [MeOH-H_2_O (30:70 → 40:60 → 50:50 → 60:40 → 100:0, *v*/*v*)], then PGE 7-7-1–PGE 7-7-20 were produced. PGE 7-7-5 (108.7 mg) was purified by pHPLC [CH_3_CN-H_2_O (12:88, *v*/*v*)] to yield corilagin (**16**, 37.3 mg). Using the same separation method, valoneic acid dilactone (**15**, 37.2 mg) was obtained from PGE 7-7-6 (112.7 mg).

PGE 7-7-10 (216.9 mg) was isolated by pHPLC [CH_3_CN-H_2_O (16:84, *v*/*v*)] to gain punicagranin F (**6**, 78.4 mg). PGE 8 (12.2 g) was prepared by Sephadex LH-20 CC (MeOH) to generate PGE 8-1–PGE 8-6. PGE 8-2 (2.6 g) was separated by pHPLC [MeOH-H_2_O (30:70 → 40:60 → 50:50 → 100:0, *v*/*v*)] to yield PGE 8-2-1–PGE 8-2-16. PGE 8-2-9 (93.0 mg) was purified by pHPLC [CH_3_CN-1% HAc (17:83, *v*/*v*)] and 4-*O*-*β*-d-glucopyranosyl-3,3′-di-*O*-methylellagic acid (**13**, 14.3 mg) was given.

PGE 8-2-10 (136.0 mg) was prepared by pHPLC [CH_3_CN-1% HAc (9:91, *v*/*v*)] to give 4-*O*-*α*-l-rhamnopyranosyl ellagic acid (**12**, 16.1 mg). PGE 8-5 (3.37 g) was isolated by pHPLC [MeOH-H_2_O (30:70 → 40:60 → 50:50 → 100:0, *v*/*v*)] and PGE 8-5-1–PGE 8-5-10 were yielded. PGE 8-5-2 (630.0 mg) was further purified by pHPLC [CH_3_CN-1% HAc (9:91, *v*/*v*)] to gain (*S*)-flavogallonic acid (**14**, 46.7 mg). PGE 8-5-4 (482.7 mg) was prepared by pHPLC [CH_3_CN-1% HAc (12:88, *v*/*v*)] to give corilagin (**16**, 234.7 mg).

H_2_O eluate (PGH, 20.0 g) was fractionated by ODS CC [MeOH-H_2_O (0:100 → 10:90 → 20:80 → 100:0, *v*/*v*)] to obtain PGH 1–PGH 5. PGH 2 (7.0 g) was pHPLC [MeOH-H_2_O (2:98 → 4:96 → 100:0, *v*/*v*)] to give PGH 2-1–PGH 2-13. PGH 2-4 (292.1 mg) was prepared by pHPLC [CH_3_CN-1% HAc (1:99, *v*/*v*)] to yield PGH 2-4-1–PGH 2-4-5. PGH 2-4-3 (37.3 mg) was subjected to Sephadex LH-20 CC (MeOH) to obtain punicagranin G (**7**, 14.4 mg).

PGH 2-4-4 (28.6mg) was purified by pHPLC [CH_3_CN-1% HAc (1:99, *v*/*v*) further, and 2,3-(*S*)-hexahydroxydiphenoyl-d-glucose (**17**, 13.9 mg) was produced. PGH 2-8 (1150.9 mg) was isolated by pHPLC [CH_3_CN-1% HAc (1:99, *v*/*v*)] to yield PGH 2-8-1–PGH 2-8-8. Among them, PGH 2-8-4 was identified as punicagranin H (**8**, 94.0 mg) and punicalin (**18**, 43.1 mg). PGH 3 (2.8 g) was fractionated by pHPLC [MeOH-H_2_O (8:92 → 10:90 → 100:0, *v*/*v*)] to give PGH 3-1–PGH 3-12.

PGH 3-6 (358.9 mg) was prepared by pHPLC [CH_3_CN-1% HAc (5:95, *v*/*v*)] to produce PGH 3-6-1–PGH 3-6-5. PGH 3-6-4 (75.3 mg) was further isolated by pHPLC [CH_3_CN-1% HAc (1:99, *v*/*v*)] to gain punicacortein C (**20**, 41.9 mg). PGH 3-11 (294.6 mg) was separated by pHPLC [MeOH-1% HAc (15:85, *v*/*v*)] and flavogallonic acid dilactone (**14**, 21.6 mg) as well as punicalagin (**19**, 42.7 mg) were yielded. PGH 4 (0.7 g) was purified by Sephadex LH-20 CC [MeOH-H_2_O (1:1, *v*/*v*)] and pHPLC [CH_3_CN-1% HAc (12:88, *v*/*v*)] in turn to give brevifolincarboxylic acid (**11**, 46.6 mg).

Punicagranin A (**1**): white powder; UV λ_max_ (MeOH) nm (log ε): 264 (3.88), 338 (3.72); IR ν_max_ (KBr) cm^−1^: 3171, 1790, 1715, 1608, 1551, 1513, 1339, 1240, 1201, 1108, 1024; ^1^H NMR (DMSO-*d*_6_, 500 MHz): δ 2.14 (3H, s, H_3_-6), 7.17 (1H, s, H-5′); ^13^C NMR (DMSO-*d*_6_, 125 MHz): δ 166.3 (C-2), 142.2 (C-3), 119.7 (C-4), 160.8 (C-5), 14.4 (C-6), 108.0 (C-1′), 143.0 (C-2′), 134.5 (C-3′), 146.4 (C-4′), 114.2 (C-5′), 121.5 (C-6′), 167.7 (C-7′); HRESIMS: *m*/*z* 279.0141 [M–H]^−^ (calcd for C_12_H_7_O_8_, 279.0146).

Punicagranin B (**2**): white powder; UV λ_max_ (MeOH) nm (log ε): 261 (3.97), 334 (3.86); IR ν_max_ (KBr) cm^−1^: 3163, 1800, 1716, 1610, 1552, 1515, 1436, 1351, 1242, 1119, 1024; ^1^H NMR (DMSO-*d*_6_, 500 MHz): δ 2.15 (3H, s, H_3_-6), 7.10 (1H, s, H-5′), 3.70 (3H, s, 7′-COO*CH*_3_); ^13^C NMR (DMSO-*d*_6_, 125 MHz): δ 166.5 (C-2), 142.5 (C-3), 120.1 (C-4), 160.7 (C-5), 14.4 (C-6), 107.9 (C-1′), 142.8 (C-2′), 135.0 (C-3′), 146.6 (C-4′), 114.3 (C-5′), 120.0 (C-6′), 166.9 (C-7′), 51.3 (7′-COO*C*H_3_); HRESIMS: *m*/*z* 293.0299 [M–H]^−^ (calcd for C_13_H_9_O_8_, 293.0303).

Punicagranin C (**3**): white powder; [α]_D_^25^ −7.0 (*c* 0.31, MeOH); UV λ_max_ (MeOH) nm (log ε): 272 (3.78); CD (*c* 0.0022 M, MeOH) mdeg (λnm): +18.3 (223); IR ν_max_ (KBr) cm^−1^: 3734, 3192, 2923, 1745, 1619, 1520, 1485, 1317, 1210, 1134, 1072; ^1^H NMR (DMSO-*d*_6_, 500 MHz): δ 7.79 (1H, br. s, H-4), 6.21 (1H, br. s, H-5), 4.94, 5.02 (1H each, both d, *J* = 19.0 Hz, H_2_-6), 6.81 (1H, s, H-5′); ^13^C NMR (DMSO-*d*_6_, 125 MHz): δ 171.2 (C-2), 127.4 (C-3), 153.3 (C-4), 71.6 (C-5), 70.6 (C-6), 125.8 (C-1′), 139.9 (C-2′), 140.1 (C-3′), 147.9 (C-4′), 102.0 (C-5′), 115.6 (C-6′), 170.0 (C-7′); HRESIMS: *m*/*z* 281.0297 [M–H]^−^ (calcd for C_12_H_9_O_8_, 281.0303).

Punicagranin D (**4**): pale yellow powder; [α]_D_^25^ −14.4 (*c* 0.14, MeOH); UV λ_max_ (MeOH) nm (log ε): 274 (3.91), 280 (3.92, sh), 344 (3.91); CD (*c* 0.0018 M, MeOH) mdeg (λnm): +36.7 (222), +3.68 (278), +2.16 (343); IR ν_max_ (KBr) cm^−1^: 3168, 2922, 2839, 1713, 1615, 1586, 1451, 1339, 1203, 1097, 1021; ^1^H NMR (DMSO-*d*_6_, 500 MHz): δ 7.42 (1H, s, H-6), 3.63 (2H, s, H_2_-11), 6.64 (1H, s, H-13), 4.09 (2H, q, *J* = 7.0 Hz, H_2_-15), 1.18 (3H, t, *J* = 7.0 Hz, H_3_-16); ^13^C NMR (DMSO-*d*_6_, 125 MHz): δ 160.7 (C-2), 116.6 (C-3), 141.8 (C-4), 109.8 (C-5), 112.1 (C-6), 149.7 (C-7), 139.6 (C-8), 139.8 (C-9), 109.6 (C-10), 31.5 (C-11), 169.2 (C-12), 92.6 (C-13), 161.2 (C-14), 60.5 (C-15), 13.9 (C-16); HRESIMS: *m*/*z* 335.0400 [M–H]^−^ (calcd for C_15_H_11_O_9_, 335.0409).

Punicagranin E (**5**): pale yellow powder; UV λ_max_ (MeOH) nm (log ε): 266 (3.75), 328 (3.91); IR ν_max_ (KBr) cm^−1^: 3732, 2923, 2861, 1716, 1587, 1514, 1470, 1373, 1255, 1208, 1094, 1048; ^1^H NMR (DMSO-*d*_6_, 500 MHz): δ 6.75 (1H, d, *J* = 8.5 Hz, H-5), 6.84 (1H, d, *J* = 8.5 Hz, H-6), 2.01 (3H, s, H_3_-11), 4.46 (2H, q, *J* = 7.0 Hz, H_2_-13), 1.35 (3H, t, *J* = 7.0 Hz, H_3_-14); ^13^C NMR (DMSO-*d*_6_, 125 MHz): δ 161.0 (C-2), 116.7 (C-3), 142.5 (C-4), 115.3 (C-5), 112.7 (C-6), 149.0 (C-7), 132.3 (C-8), 142.3 (C-9), 108.7 (C-10), 13.8 (C-11), 165.1 (C-12), 62.2 (C-13), 13.9 (C-14); HRESIMS: *m*/*z* 263.0557 [M–H]^−^ (calcd for C_13_H_11_O_6_, 263.0561).

Punicagranin F (**6**): pale yellow powder; [α]_D_^25^ −15.0 (*c* 0.10, MeOH); UV λ_max_ (MeOH) nm (log ε): 256 (4.68), 372 (4.11); CD (*c* 0.00020 M, MeOH) mdeg (λnm): −0.80 (233), −1.52 (255), −0.06 (284), −0.07 (298), +0.05 (326). IR ν_max_ (KBr) cm^−1^: 3246, 1720, 1600, 1521, 1442, 1336, 1235, 1181, 1058, 887, 767; ^1^H NMR (DMSO-*d*_6_, 500 MHz): δ 7.50 (1H, s, H-5), 7.11 (1H, s, H-6″), 3.86 (2H, q, *J* = 7.0 Hz, 7″-OC*H*_2_CH_3_), 0.87 (3H, t, *J* = 7.0 Hz, 7″-OCH_2_C*H*_3_); ^13^C NMR (DMSO-*d*_6_, 125 MHz): δ 112.6 (C-1), 136.3 (C-2)*^a^*, 139.4 (C-3), 147.7 (C-4), 109.9 (C-5), 107.1 (C-6), 159.2 (C-7), 107.2 (C-1′)*^b^*, 135.4 (C-2′)*^a^*, 138.5 (C-3′)*^a^*, 146.1 (C-4′)*^c^*, 111.5 (C-5′)*^b^*, 119.2 (C-6′)*^b^*, 157.4 (C-7′), 125.3 (C-1″), 117.9 (C-2″), 143.4 (C-3″)*^c^*, 137.2 (C-4″), 143.9 (C-5″), 109.1 (C-6″), 165.9 (C-7″), 59.2 (7″-O*C*H_2_CH_3_), 13.6 (7″-OCH_2_*C*H_3_) (*^a,b,c^*the values with the same superscript markers may be interchangeable); HRESIMS: *m*/*z* 497.0358 [M–H]^−^ (calcd for C_23_H_23_O_13_, 497.0362).

Punicagranin G (**7**) (1α:1β ≈ 1:2): pale yellow powder; [α]_D_^25^ −73.9 (*c* 0.17, H_2_O); UV λ_max_ (MeOH) nm (log ε): 254 (4.73), 372 (4.16); CD (*c* 0.00013 M, MeOH) mdeg (*λ*nm): −4.20 (234), +1.36 (276), −0.21 (299), −0.07 (298), +1.29 (328), −1.87 (381); IR ν_max_ (KBr) cm^−1^: 3290, 2921, 1698, 1601, 1548, 1440, 1339, 1223, 1058; ^1^H NMR (CD_3_OD, 400 MHz): δ 4.81 (1H, d, *J* = 3.6 Hz, Glc-H-1α), 2.46 (1H, dd, *J* = 3.6, 9.6 Hz, Glc-H-2α), 4.83 (1H, dd, *J* = 9.6, 10.0 Hz, Glc-H-3α), 1.75 (1H, dd, *J* = 10.0, 10.0 Hz, Glc-H-4α), 3.55 (1H, m, Glc-H-5α), [3.55 (1H, br. d, ca. *J* = 12 Hz), 4.67 (1H, dd, *J* = 3.2, 12.0 Hz), Glc-H_2_-6α], 7.33 (1H, s, H-5′α), 7.17 (1H, s, H-5″α); 4.25 (1H, d, *J* = 7.6 Hz, Glc-H-1β), 2.28 (1H, dd, *J* = 7.6, 9.2 Hz, Glc-H-2β), 4.57 (1H, dd, *J* = 9.2, 9.6 Hz, Glc-H-3β), 1.79 (1H, dd, *J* = 9.6, 9.6 Hz, Glc-H-4β), 3.03 (1H, m, Glc-H-5β), [3.53 (1H, br. d, ca. *J* = 12 Hz), 4.75 (1H, dd, *J* = 3.2, 12.4 Hz), Glc-H_2_-6β], 7.33 (1H, s, H-5′β), 7.13 (1H, s, H-5″β); ^13^C NMR (CD_3_OD, 100 MHz): δ 93.6 (Glc-C-1α), 71.8 (Glc-C-2α), 76.8 (Glc-C-3α), 69.1 (Glc-C-4α), 70.1 (Glc-C-5α), 62.5 (Glc-C-6α); 98.7 (Glc-C-1β), 74.2 (Glc-C-2β), 78.6 (Glc-C-3β), 68.8 (Glc-C-4β), 74.9 (Glc-C-5β), 62.1 (Glc-C-6β); the signals for gallagyl group linked to α-d-glucose: δ 108.7, 110.7, 112.0 (C-5″), 112.9 (C-5′), 115.0, 115.3, 118.3 (C-1″), 119.3 (C-1′), 120.9 (C-6′), 121.9 (C-6″), 124.9, 126.3, 137.3, 137.8, 138.9 (C-3″), 139.4 (C-3′), 140.5, 140.6, 144.6, 144.8, 145.8 (C-4′), 145.9 (C-4″), 147.2, 147.6, 160.0 (lactone carbonyl-C), 160.8 (lactone carbonyl-C), 169.1 (C-7′), 170.7 (C-7″); the signals for gallagyl group linked to β-d-glucose: δ 108.9, 110.7, 111.9 (C-5″), 113.0 (C-5′), 114.9, 115.2, 118.1 (C-1″), 119.3 (C-1′), 120.9 (C-6′), 121.7 (C-6″), 124.7, 125.9, 137.3, 137.9, 138.8 (C-3″), 139.5 (C-3′), 140.5, 140.6, 144.5, 144.8, 145.8 (C-4′), 145.9 (C-4″), 147.1, 147.7, 159.9 (lactone carbonyl-C), 160.8 (lactone carbonyl-C), 169.0 (C-7′), 170.7 (C-7″); HRESIMS: *m*/*z* 781.0530 [M–H]^−^ (calcd for C_34_H_21_O_22_, 781.0530).

Punicagranin H (**8**) (1α:1β ≈ 1:3): pale yellow powder; [α]_D_^25^ −85.1 (*c* = 0.54, H_2_O); UV λ_max_ (MeOH), nm (log *ε*): 256 (4.66), 373 (4.06); CD (*c* 0.00014 M, MeOH) mdeg (*λ*nm): −7.23 (232), +9.48 (276), +1.49 (306), +4.59 (335), −5.12 (385); IR ν_max_ (KBr) cm^−1^: 3361, 2911, 2704, 1708, 1600, 1520, 1440, 1350, 1222, 1176, 1057, 877, 751; ^1^H NMR (CD_3_OD, 400 MHz): δ 3.44, 3.51 (1H each, both d, *J* = 11.6 Hz, Fruc-H_2_-1*α*), 3.91 (1H, s, Fruc-H-3α), 4.09 (1H, d, *J* = 2.8 Hz, Fruc-H-4α), 2.67 (1H, br. d, ca. *J* = 11 Hz, Fruc-H-5α), [3.12 (1H, br. d, ca. *J* = 10 Hz), 3.87 (1H, dd, *J* = 10.4, 10.8 Hz), Fruc-H_2_-6α], 6.92 (1H, s, H-5′α), 7.12 (1H, s, H-5″α); 3.23, 3.48 (1H each, both d, *J* = 11.6 Hz, Fruc-H_2_-1β), 3.93 (1H, d, *J* = 1.6 Hz, Fruc-H-3β), 4.38 (1H, t, *J* = 1.6 Hz, Fruc-H-4β), 2.36 (1H, br. d, ca. *J* = 11 Hz, Fruc-H-5β), [3.28 (1H, br. d, ca. *J* = 11 Hz), 4.03 (1H, dd, *J* = 11.2, 11.2 Hz), Fruc-H_2_-6β], 6.89 (1H, s, H-5′β), 7.15 (1H, s, H-5″β); ^13^C NMR (CD_3_OD, 100 MHz): δ 64.6 (Fruc-C-1α), 107.2 (Fruc-C-2α), 78.9 (Fruc-C-3α), 83.2 (Fruc-C-4α), 80.8 (Fruc-C-5α), 67.6 (Fruc-C-6α); 65.4 (Fruc-C-1β), 106.1 (Fruc-C-2β), 74.9 (Fruc-C-3β), 82.9 (Fruc-C-4β), 80.7 (Fruc-C-5β), 68.2 (Fruc-C-6β); the signals for gallagyl group linked to α-d-fructose: δ 109.9, 111.3, 111.4 (C-5′), 112.5 (C-5″), 115.2, 115.6, 117.5 (C-1′), 118.8 (C-1″), 121.8 (C-6″), 122.7, 123.5, 125.3, 137.1, 137.6, 138.5 (C-3′), 139.2 (C-3″), 140.1, 141.2, 144.6, 144.9, 145.9 (C-4′), 145.9 (C-4″), 147.9, 148.1, 160.2 (lactone carbonyl-C), 160.4 (lactone carbonyl-C), 169.4 (C-7″), 170.0 (C-7′); the signals for gallagyl group linked to β-d-fructose: δ 109.9, 110.8, 111.0 (C-5′), 112.6 (C-5″), 115.2, 115.4, 117.5 (C-1′), 118.7 (C-1″), 121.6 (C-6″), 122.9, 123.7, 125.5, 137.2, 138.1, 138.5 (C-3′), 139.2 (C-3″), 140.1, 141.2, 144.7, 145.0, 145.9 (C-4′), 145.9 (C-4″), 148.1, 148.2, 160.3 (lactone carbonyl-C), 160.5 (lactone carbonyl-C), 169.3 (C-7″), 169.7 (C-7′); HRESIMS: *m*/*z* 781.0519 [M–H]^−^ (calcd for C_34_H_21_O_22_, 781.0530).

Punicagranin H (**9**): white powder; UV λ_max_ (MeOH) nm (log ε): 260 (4.10); IR ν_max_ (KBr) cm^−1^: 3318, 2924, 1718, 1606, 1512, 1457, 1340, 1266, 1168, 1076, 851, 765; ^1^H NMR (CD_3_OD, 500 MHz): δ 6.37 (1H, d, *J* = 2.5 Hz, H-3), 6.65 (1H, d, *J* = 2.5 Hz, H-5), 3.60, 3.66 (1H each, both d, *J* = 16.5 Hz, H_2_-7), 3.53 (3H, s, 8-COOC*H*_3_), 7.98 (2H, d, *J* = 8.5 Hz, H-2′,6′), 6.90 (2H, d, *J* = 8.5 Hz, H-3′,5′), 4.86 (1H, d, *J* = 7.5 Hz, H-1″), 3.46 (1H, dd, *J* = 7.5, 9.0 Hz, H-2″), 3.47 (1H, dd, *J* = 9.0, 9.0 Hz, H-3″), 3.41 (1H, dd, *J* = 9.0, 9.0 Hz, H-4″), 3.42 (1H, m, H-5″), [3.72 (1H, dd, *J* = 4.5, 12.0 Hz), 3.91 (1H, br. d, ca. *J* = 12 Hz), H_2_-6″]; ^13^C NMR (CD_3_OD, 125 MHz): δ 111.0 (C-1), 152.1 (C-2), 105.4 (C-3), 159.0 (C-4), 102.4 (C-5), 158.5 (C-6), 30.1 (C-7), 174.3 (C-8), 52.4 (8-COO*C*H_3_), 121.1 (C-1′), 133.5 (C-2′,6′), 116.5 (C-3′,5′), 164.3 (C-4′), 166.0 (C-7′), 103.3 (C-1″), 74.3 (C-2″), 78.0 (C-3″), 71.3 (C-4″), 78.3 (C-5″), 62.6 (C-6″); HRESI-TOF-MS: *m*/*z* 479.1188 [M–H]^−^ (calcd for C_22_H_23_O_12_, 479.1195).

#### 4.1.4. Acid Hydrolysis of **7**–**9**

A solution of compounds **7**–**9** (each 1.5 mg) in 1 M HCl (1 mL) was heated under reflux, neutralized with Amberlite IRA-400 (OH^−^ form) and filtrated successively. Each aqueous layer was analyzed by HPLC: HPLC column, Kaseisorb LC NH_2_-60-5, 4.6 mm i.d. × 250 mm (Tokyo Kasei Co., Ltd., Tokyo, Japan); mobile phase, CH_3_CN-H_2_O [(75:25; flow rate, 1.0 mL/min)]. The results were d-glucose (t*_R_*: 8.1 min, positive optical rotation) for **7** and **9**, d-fructose (t*_R_*: 7.5 min, negative) for **8** were identified by comparison of their retention times and optical rotations with those of the authentic sample.

### 4.2. Materials and Methods for Anti-Inflammatory Assay

#### 4.2.1. Cell Culture

RAW 264.7 cells (Chinese Academy of Medical Science) were cultured in DMEM (Biological Industries, Beit HaEmek, Israel) supplemented with 10% heat-inactivated FBS (Biological Industries, Beit HaEmek, Israel), 100 U/mL penicillin (Waltham, MA, USA) and 100 µg/mL streptomycin (Waltham, MA, USA) in a 5% CO_2_ humidified incubator at 37 °C. When its confluency reached 80–90% approximately, the cells were seeded in 96-well plates.

#### 4.2.2. Cytotoxicity Assay

RAW 264.7 macrophages-like cells were seeded in 96-well plates (2 × 10^5^ cells/mL) and incubated for 24 h. After that, the supernatant was removed and RAW 264.7 cells were treated without or with test compounds (their primary concentration was 100 mM, and these solutions were then diluted to be 30 μM for compounds **1**–**18** and **20** and 15 μM for compound **19** as final concentration, respectively) for 18 h, respectively. Then, the supernatant was replaced with 0.5 mg/mL of MTT solution and incubated further. After 4 h, the supernatant was discarded, and the pellet at the bottom of the 96-well plate was dissolved with DMSO, measured at 490 nm using a BioTek Cytation five-cell imaging multi-mode reader (Winooski, VT, USA).

#### 4.2.3. Determination of NO Production

The in vitro NO production inhibitory assay was conducted as what has been reported [29]. In the experiment, four groups, including the normal group, LPS group, the positive drug DEX group and the tested groups, were settled. RAW 264.7 cells were seeded at the 96-well plates of 2 × 10^6^ cells/mL and incubated for 24 h to facilitate complete adherence to the well. The primary compound solution was the same as that in Section 4.2.2, with a concentration at 100 mM, and then, these solutions were diluted to 3, 10 and 30 μM for compounds **1**, **3**, **5**–**8**, **10**, **11, 14, 16**–**18** and **20** and 1, 5 and 15 μM for compound **19**, respectively.

Then, the media of the normal group was replaced with serum-containing medium, LPS (0.5 μg/mL) was administered to the LPS group, LPS (0.5 μg/mL) combined with positive drug DEX (1.5 μg/mL) was given to the DEX group, and the tested compounds together with LPS (0.5 μg/mL) were applied to the tested groups, followed by incubation 18 h. After, equal supernatant of the RAW 264.7 culture and the Griess reagent from Beyotime Biotechnology (Shanghai, China) were mixed and measured at 540 nm on a BioTek Cytation five-cell imaging multi-mode reader (Winooski, VT, USA). A standard sodium nitrite curve was used to calculate the amount of NO.

#### 4.2.4. Western Blot Analysis

RAW 264.7 cells were seeded in the 6-cell plates for 24 h. Then, the supernatant of normal group, LPS group, the positive drug DEX group and the tested groups were replaced with serum-containing DMEM, LPS (0.5 μg/mL), LPS (0.5 μg/mL) as well as DEX (1.5 μg/mL) and LPS (0.5 μg/mL) combined with the tested compounds (final concentration was 30 μM), respectively. After 18 h, the RAW 264.7 cells were separated from the 6-cell plates (4 × 10^6^ cells/mL) and then centrifuged at 4 °C and 12,000× *g* for 5 min. The cells were lysed by adding mixed solution of RIPA lysis buffer, protease and phosphatase inhibitor with the ratio of 100:1:1 for 30 min on ice.

Then, the supernatant was collected by centrifuging at 12,000× *g* for 5 min, and the total protein content was determined by using a BCA protein quantification kit (Thermo Fisher Scientific, Waltham, MA, USA). Next, the protein sample was mixed with 4 × sample buffer, incubated at 100 °C for 5 min, loaded onto 10% sodium dodecyl sulfate (SDS) polyacrylamide gel and electrophoresed. The electrophoretic SDS polyacrylamide gel was transferred onto polyvinylidene fluoride membranes (Merch/Millipore, Schwalbach, Germany).

They were probed with primary antibodies against NF-κB (1:1000, ab16502, Abcam, CBG, UK), phosphorylated NF-κB (p-NF-κB) (1:1000, ab16502, Abcam), p38 MAPK (1:1000, 8690, CST), phosphorylated p38 MAPK (1:1000, 4511, CST), IL-6 (1:1000, 21865-1-AP, Proteintech), iNOS (1:1000, ab3523, Abcam), COX-2 (1:1000, ab52237, Abcam), β-actin (1:1000, ab8227, Abcam), IκBα (1:1000, ab32518, Abcam), phosphorylated IκBα (1:1000, ab92700, Abcam), IKK (1:1000, 2682S, CST) and phosphorylated IKK (1:1000, 2697, CST) and incubated overnight at 4 °C.

Then, the membranes were washed three times, each for 10 min with Tris-buffered saline with 0.1% Tween 20 (TBST), followed by treatment with horseradish peroxidase (HRP)-conjugated goat anti-rabbit immunoglobulin G (IgG) (1:10,000, SR238; Beijing Solarbio Science & Technology, Beijing, China) at room temperature for 1 h and then washed with TBST buffer three times for 10 min. Finally, the immunoreactive protein bands were measured by Immobilon Western Chemilumescent HRP Substrate (Millipore, Massachusetts, USA), visualized using a ChemiDoc MP Imaging System (Bio-Rad Laboratories, Hercules, USA) and analyzed using Image Lab software (Version 1.0, National Institutes of Health, Bethesda, MD, USA).

## Figures and Tables

**Table 1:** Inhibitory effects of compounds **1**–**20** on NO production in RAW 264.7 cells.

No.	NRC (%)	No.	NRC (%)	No.	NRC (%)
Normal	4.0 ± 0.9	**6**	85.9 ± 3.2 ***	**14**	88.2 ± 4.9 ***
Control	100 ± 2.9	**7**	84.1 ± 3.0 ***	**15**	99.2 ± 2.2
Dex	83.2 ± 2.8 ***	**8**	80.2 ± 3.6 ***	**16**	88.7 ± 1.8 ***
**1**	93.4 ± 3.7 *	**9**	97.6 ± 3.0	**17**	90.2 ± 1.4 ***
**2**	101.4 ± 1.3	**10**	81.9 ± 4.1 ***	**18**	74.8 ± 2.8 ***
**3**	94.4 ± 2.5 *	**11**	91.7 ± 1.8 ***	**19**	71.0 ± 1.3 ***
**4**	100.1 ± 1.9	**12**	100.6 ± 2.5	**20**	71.1 ± 2.0 ***
**5**	91.3 ± 3.3 **	**13**	101.6 ± 2.7		

Positive control: Dexamethasone (Dex). Nitrite relative concentration (NRC): percentage of control group (set as 100%). Values represent the mean ± SD of six determinations. * *p* < 0.05, ** *p* < 0.01, *** *p* < 0.001 (Differences between the compound-treated group and control group). Final concentrations were 30 μM for compounds **1**–**18** and **20**, as well as 15 μM and 1.5 μg/mL for **19** and Dex, respectively.

## Data Availability

Not applicable.

## Supplementary Materials

The following supporting information can be downloaded at: https://www.mdpi.com/article/10.3390/molecules27144622/s1, Supplementary data (the NMR and HRESIMS spectra of compounds **1**–**9**, ECD spectra of compounds **3**, **4**, **6**–**8**, **14**, **16**–**20**, cell viability assay, the raw data for western blot assays, and the physical and chemical data of compounds **10**–**20** are available in the Supporting Information.

## Sample Availability

Not available.
