# Supplementary material for: Polyphenols from the Peels of Punica granatum L. and Their Bioactivity of Suppressing Lipopolysaccharide-Stimulated Inflammatory Cytokines and Mediators in RAW 264.7 Cells via Activating p38 MAPK and NF-κB Signaling Pathways
Source: Molecules. 2022 Jul 20;27(14):4622. doi: 10.3390/molecules27144622 (PMC9318460; doi:10.3390/molecules27144622)
Supplement: Supplementary file 1 [file molecules-27-04622-s001.zip › molecules-1820998-supplementary.pdf]

Supporting Information

# Polyphenols from the Peels of *Punica granatum* L. and Their Bioactivity of Suppressing Lipopolysaccharide-Stimulated Inflammatory Cytokines and Mediators in RAW 264.7 Cells *via* Activating p38 MAPK and NF-κB Signaling Pathways

**Hui-Min Li [1,†], Ongher Kouye [1,†], Ding-Shan Yang [2], Ya-Qi Zhang [2], Jing-Ya Ruan [1], Li-Feng Han [1], Yi Zhang [1,2,*], Tao Wang [1,2,*]**

[1] State Key Laboratory of Component-based Chinese Medicine, Tianjin University of Traditional Chinese Medicine, 10 Poyanghu Road, West Area, Tuanbo New Town, Jinghai District, 301617, Tianjin, China; 15380711687@163.com (H.-M.L.); kouye2022@163.com (O.K.); Ruanjy19930919@163.com (J.-Y.R.); hanlifeng_1@sohu.com (L.-F.H.);

[2] I Tianjin Key Laboratory of TCM Chemistry and Analysis, Tianjin University of Traditional Chinese Medicine, 10 Poyanghu Road, West Area, Tuanbo New Town, Jinghai District, 301617 Tianjin, China; yangdingshan1996@163.com (D.-S.Z.); Zhangyaq1997@163.com (Y.-Q.Z.);

* Correspondence: zhwwxzh@tjutcm.edu.cn (Y.Z.); wangtao@tjutcm.edu.cn (T.W.); Tel./Fax: +86-22-5959-6168 (T.W.)

† These authors contributed equally to this manuscript.

**Figure S1.** $^1H$ NMR (500 MHz, DMSO-$d_6$) spectrum of **1**....4
**Figure S2.** $^{13}C$ NMR (125 MHz, DMSO-$d_6$) spectrum of compound **1**....4
**Figure S3.** HSQC (DMSO-$d_6$) spectrum of compound **1**....5
**Figure S4.** HMBC (DMSO-$d_6$) spectrum of compound **1**....5
**Figure S5.** HRESIMS spectrum of compound **1**....6
**Figure S6.** $^1H$ NMR (500 MHz, DMSO-$d_6$) spectrum of compound **2**....7
**Figure S7.** $^{13}C$ NMR (125 MHz, DMSO-$d_6$) spectrum of compound **2**....7
**Figure S8.** DEPT (125 MHz, DMSO-$d_6$) spectrum of compound **2**....8
**Figure S9.** HSQC (DMSO-$d_6$) spectrum of compound **2**....8
**Figure S10.** HMBC (DMSO-$d_6$) spectrum of compound **2**....9
**Figure S11.** HRESIMS spectrum of compound **2**....9
**Figure S12.** $^1H$ NMR (500 MHz, DMSO-$d_6$) spectrum of compound **3**....10
**Figure S13.** $^{13}C$ NMR (125 MHz, DMSO-$d_6$) spectrum of compound **3**....10
**Figure S14.** DEPT (125 MHz, DMSO-$d_6$) spectrum of compound **3**....11
**Figure S15.** $^1H$ $^1H$ COSY (DMSO-$d_6$) spectrum of compound **3**....11
**Figure S16.** HSQC (DMSO-$d_6$) spectrum of compound **3**....12
**Figure S17.** HMBC (DMSO-$d_6$) spectrum of compound **3**....12
**Figure S18.** HRESIMS spectrum of compound **3**....13
**Figure S19.** The experimental ECD spectrum of compound **3** in MeOH....13
**Figure S20.** $^1H$ NMR (500 MHz, DMSO-$d_6$) spectrum of compound **4**....14
**Figure S21.** $^{13}C$ NMR (125 MHz, DMSO-$d_6$) spectrum of compound **4**....14
**Figure S22.** DEPT (125 MHz, DMSO-$d_6$) spectrum of compound **4**....15
**Figure S23.** $^1H$ $^1H$ COSY (DMSO-$d_6$) spectrum of compound **4**....15
**Figure S24.** HSQC (DMSO-$d_6$) spectrum of compound **4**....16
**Figure S25.** HMBC (DMSO-$d_6$) spectrum of compound **4**....16
**Figure S26.** HRESIMS spectrum of compound **4**....17
**Figure S27.** The experimental ECD spectrum of compound **4** in MeOH....17
**Figure S28.** $^1H$ NMR (500 MHz, DMSO-$d_6$) spectrum of compound **5**....18
**Figure S29.** $^{13}C$ NMR (125 MHz, DMSO-$d_6$) spectrum of compound **5**....18
**Figure S30.** DEPT (125 MHz, DMSO-$d_6$) spectrum of compound **5**....19
**Figure S31.** $^1H$ $^1H$ COSY (DMSO-$d_6$) spectrum of compound **5**....19
**Figure S32.** HSQC (DMSO-$d_6$) spectrum of compound **5**....20
**Figure S33.** HMBC (DMSO-$d_6$) spectrum of compound **5**....20
**Figure S34.** HRESIMS spectrum of compound **5**....21
**Figure S35.** $^1H$ NMR (500 MHz, DMSO-$d_6$) spectrum of compound **6**....22
**Figure S36.** $^{13}C$ NMR (125 MHz, DMSO-$d_6$) spectrum of compound **6**....22
**Figure S37.** DEPT (125 MHz, DMSO-$d_6$) spectrum of compound **6**....23
**Figure S38.** $^1H$ $^1H$ COSY (DMSO-$d_6$) spectrum of compound **6**....23
**Figure S39.** HSQC (DMSO-$d_6$) spectrum of compound **6**....24
**Figure S40.** HMBC (DMSO-$d_6$) spectrum of compound **6**....24
**Figure S41.** HRESIMS spectrum of compound **6**....25
**Figure S42.** The experimental ECD spectrum of compound **6** in MeOH....25
**Figure S43.** $^1H$ NMR (400 MHz, $CD_3OD$) spectrum of compound **7**....26
**Figure S44.** $^{13}C$ NMR (100 MHz, $CD_3OD$) spectrum of compound **7**....26
**Figure S45.** DEPT (100 MHz, $CD_3OD$) spectrum of compound **7**....27
**Figure S46.** $^1H$ $^1H$ COSY ($CD_3OD$) spectrum of compound **7**....27
**Figure S47.** HSQC ($CD_3OD$) spectrum of compound **7**....28
**Figure S48.** HMBC ($CD_3OD$) spectrum of compound **7**....28
**Figure S49.** HRESIMS spectrum of compound **7**....29
**Figure S50.** The experimental ECD spectrum of compound **7** in MeOH....29

**Figure S51.** $^{1}H$ NMR (400 MHz, $CD_3OD$) spectrum of compound **8**........30
**Figure S52.** $^{13}C$ NMR (100 MHz, $CD_3OD$) spectrum of compound **8**........30
**Figure S53.** $^{1}H$ $^{1}H$ COSY ($CD_3OD$) spectrum of compound **8**........31
**Figure S54.** HSQC ($CD_3OD$) spectrum of compound **8**........31
**Figure S55.** HMBC ($CD_3OD$) spectrum of compound **8**........32
**Figure S56.** HRESIMS spectrum of compound **8**........32
**Figure S57.** The experimental ECD spectrum of compound **8** in MeOH........33
**Figure S58.** $^{1}H$ NMR (500 MHz, $CD_3OD$) spectrum of compound **9**........34
**Figure S59** $^{13}C$ NMR (125 MHz, $CD_3OD$) spectrum of compound **9**........34
**Figure S60.** DEPT (125 MHz, $CD_3OD$) spectrum of compound **9**........35
**Figure S61.** $^{1}H$ $^{1}H$ COSY ($CD_3OD$) spectrum of compound **9**........35
**Figure S62.** HSQC ($CD_3OD$) spectrum of compound **9**........36
**Figure S63.** HMBC ($CD_3OD$) spectrum of compound **9**........36
**Figure S64.** HRESIMS spectrum of compound **9**........37
**Figure S65.** The LC-MS analysis of compounds **2**, **4**, **5**, **6** and **9** from *P. granatum* peels........38
**Figure S66.** The experimental ECD spectrum of compound **14** in MeOH........41
**Figure S67.** The experimental ECD spectrum of compound **16** in MeOH........41
**Figure S68.** The experimental ECD spectrum of compound **17** in MeOH........42
**Figure S69.** The experimental ECD spectrum of compound **18** in MeOH........42
**Figure S70.** The experimental ECD spectrum of compound **19** in MeOH........43
**Figure S71.** The experimental ECD spectrum of compound **20** in MeOH........43
**Figure S72.** The MTT assay of compounds **1–18, 20** at 30 μM and **19** at 20 μM........44
**Figure S73.** The raw data of Figure 4........45
**Physical and chemical data of compounds 10–20**........46

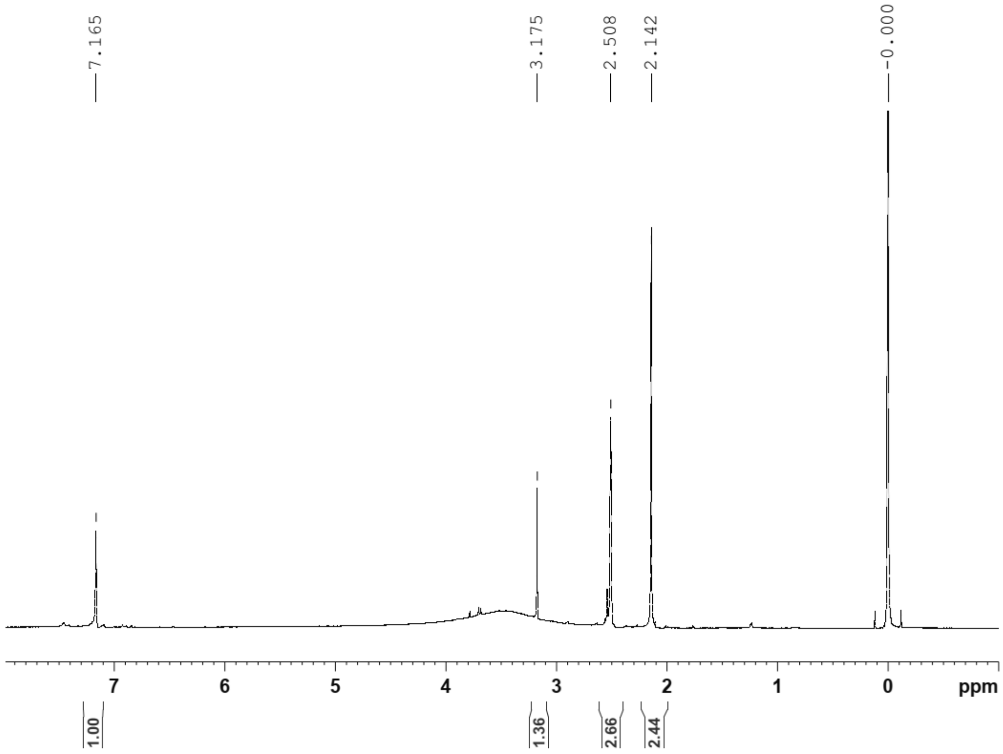


**Figure S1.** $^{1}H$ NMR (500 MHz, DMSO-$d_6$) spectrum of **1**

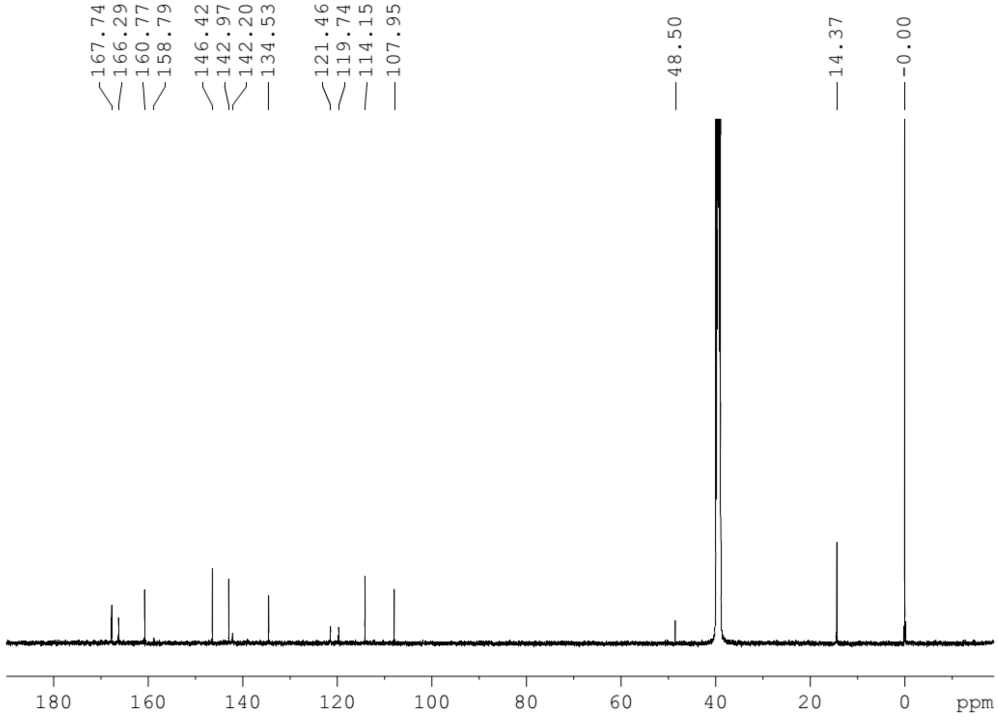


**Figure S2.** $^{13}C$ NMR (125 MHz, DMSO-$d_6$) spectrum of **1**

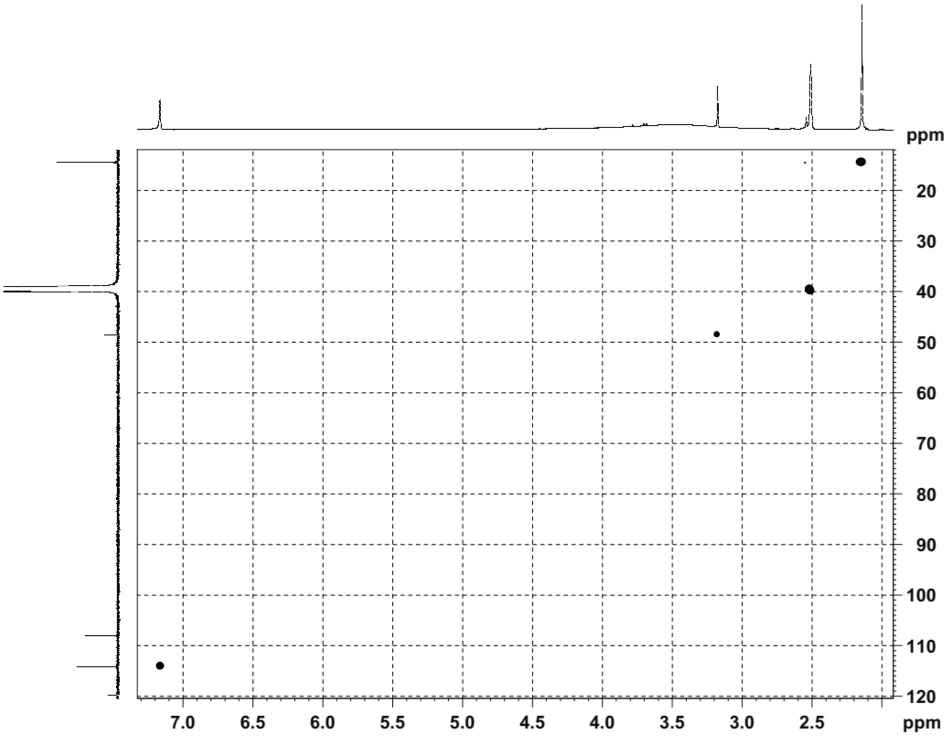


**Figure S3.** HSQC (DMSO-$d_6$) spectrum of **1**

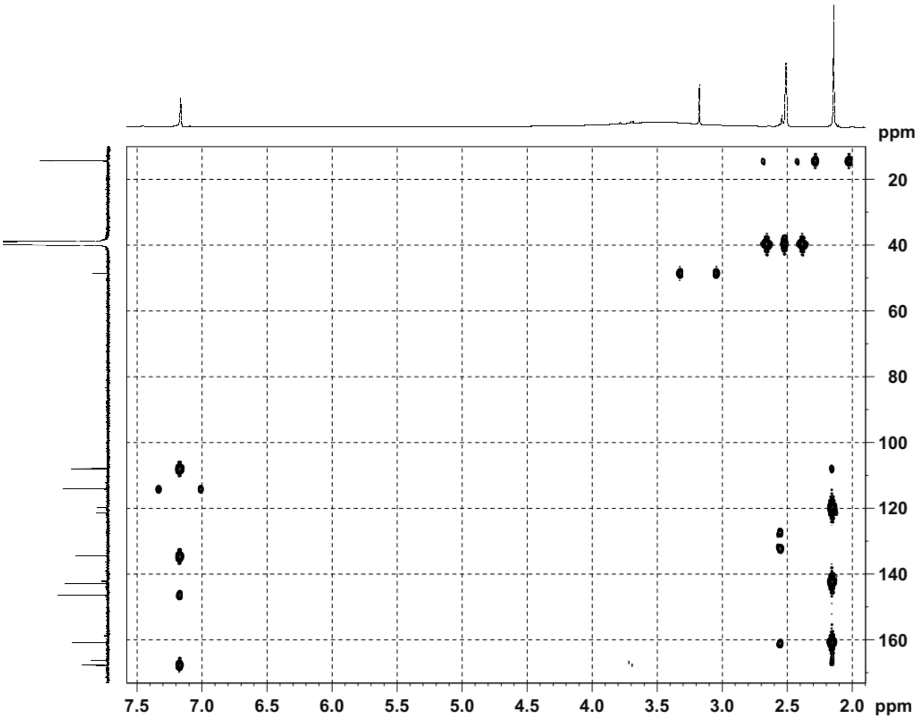


**Figure S4.** HMBC (DMSO-$d_6$) spectrum of **1**

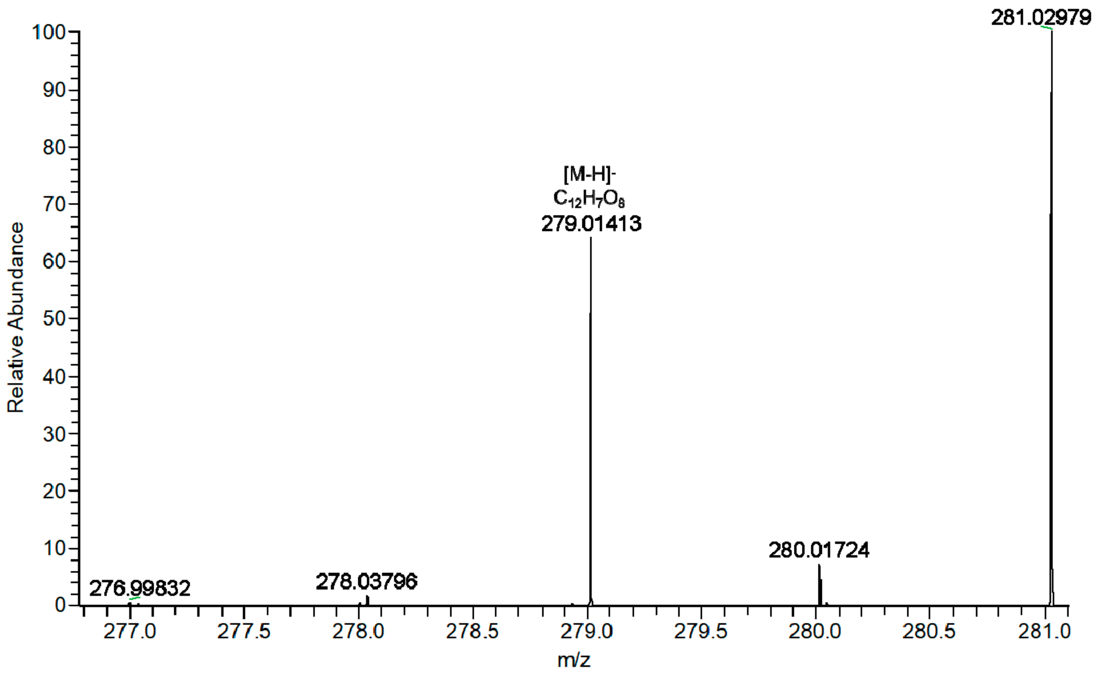


**Figure S5.** HRESIMS spectrum of **1**

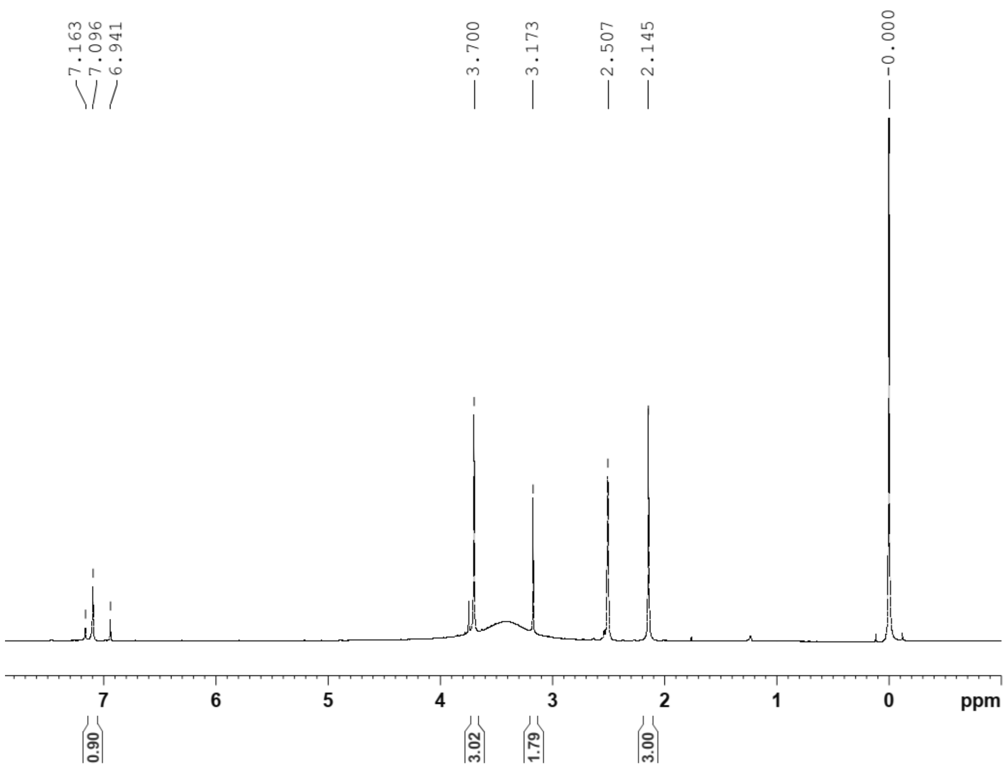


**Figure S6.** $^1$H NMR (500 MHz, DMSO-$d_6$) spectrum of **2**

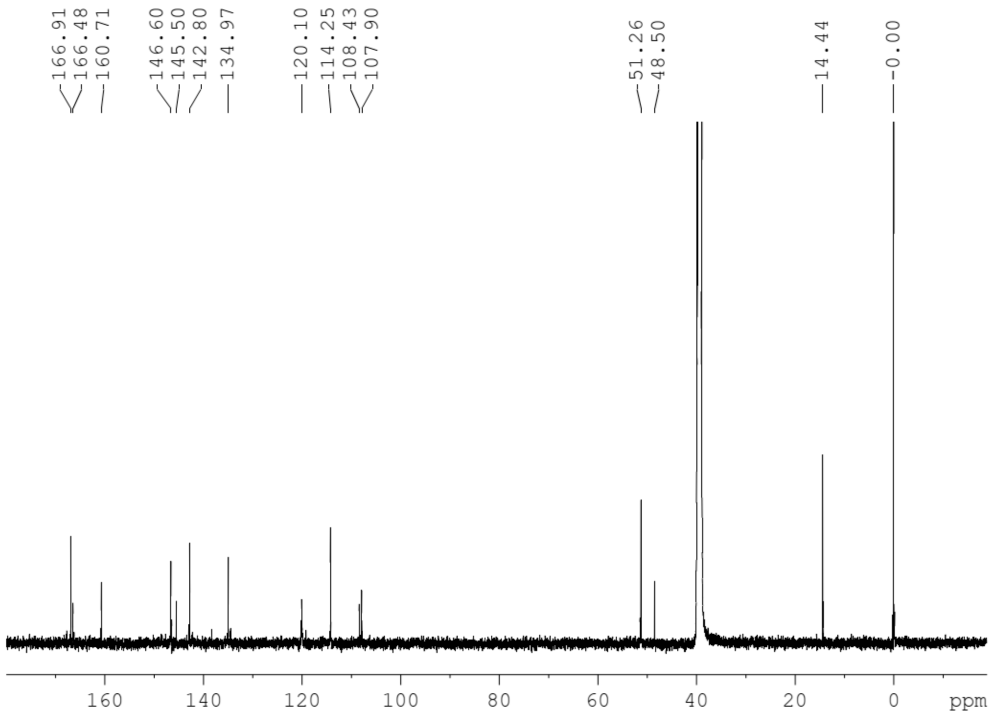


**Figure S7.** $^{13}$C NMR (125 MHz, DMSO-$d_6$) spectrum of **2**

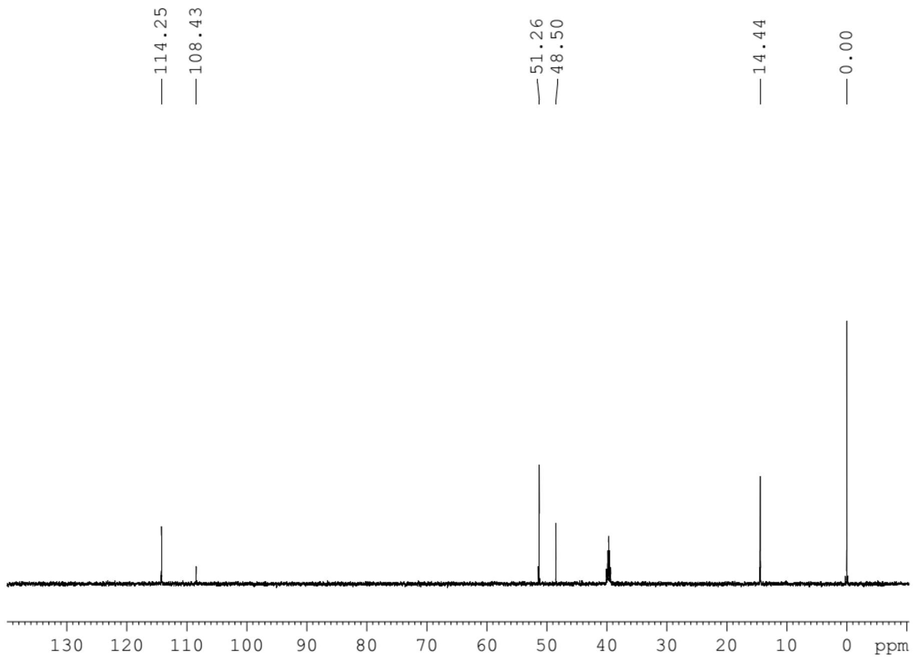


**Figure S8.** DEPT (125 MHz, DMSO-$d_6$) spectrum of **2**

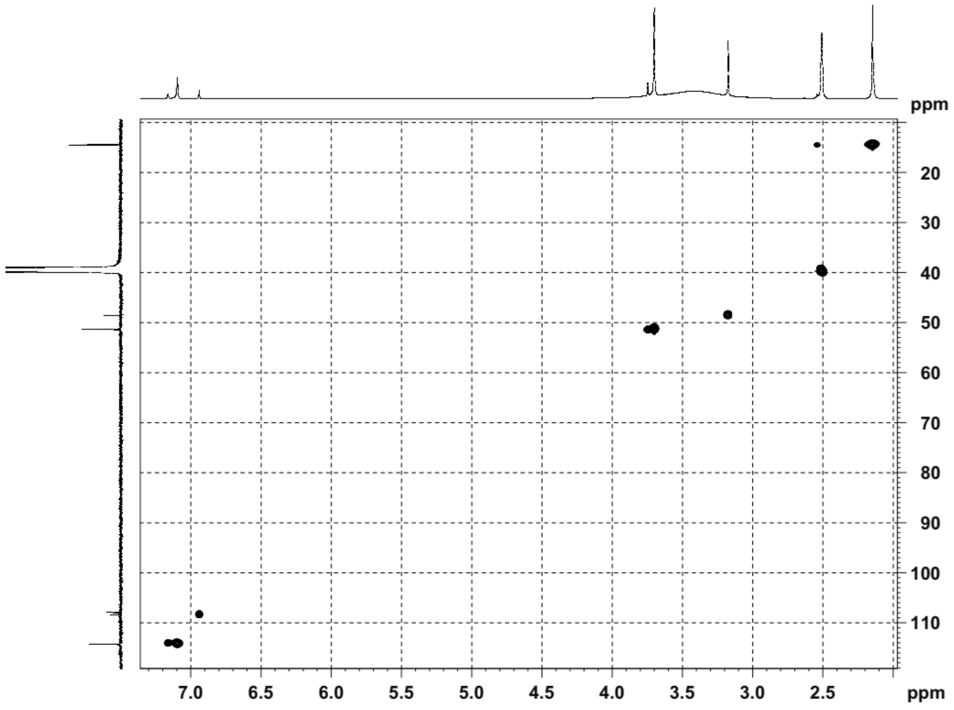


**Figure S9.** HSQC (DMSO-$d_6$) spectrum of **2**

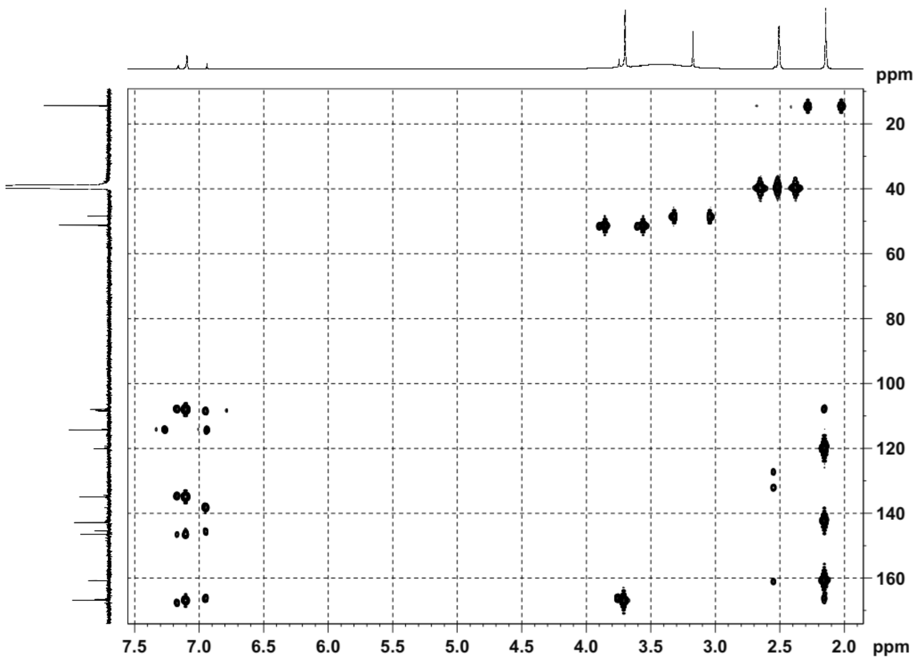


**Figure S10. S11** HMBC (DMSO-*d*6) spectrum of **2**

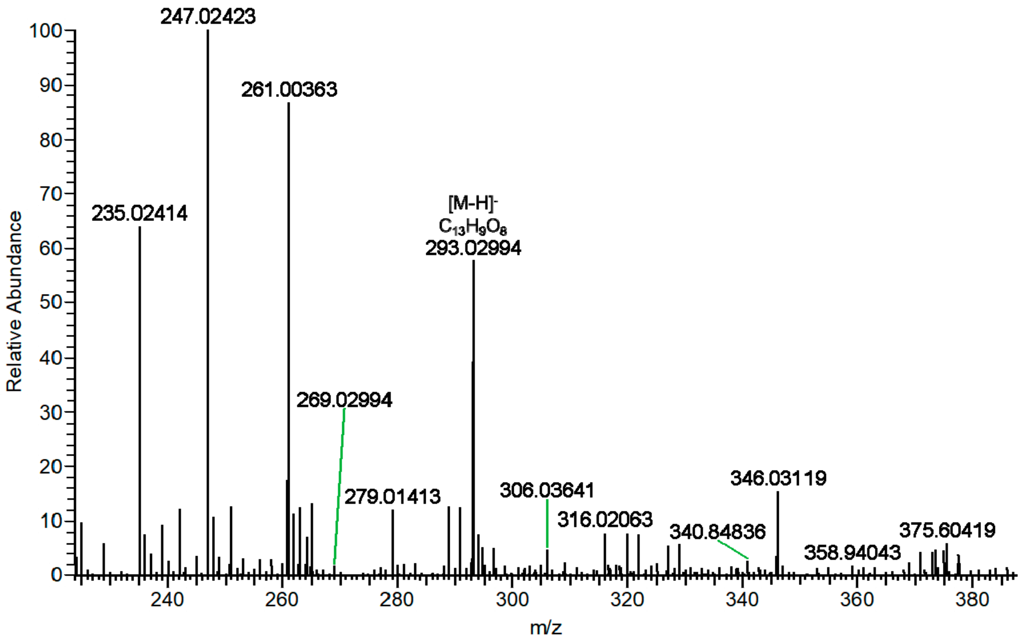


**Figure S11.** HRESIMS spectrum of **2**

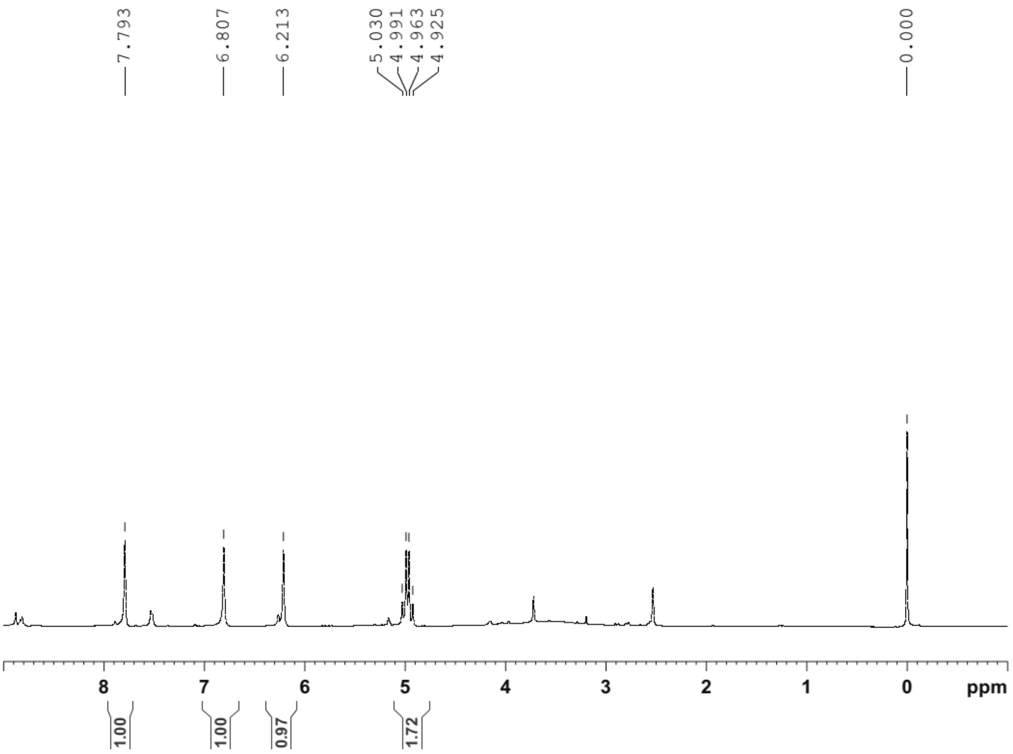


**Figure S12.** $^{1}H$ NMR (500 MHz, DMSO-$d_6$) spectrum of **3**

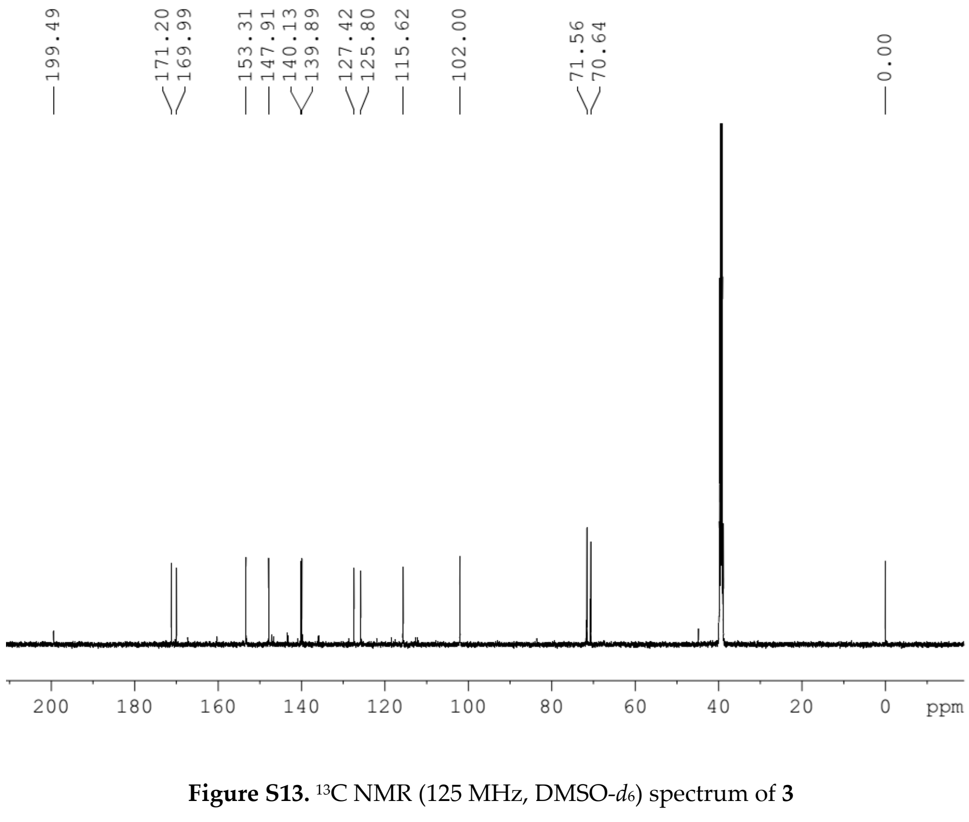


**Figure S13.** $^{13}C$ NMR (125 MHz, DMSO-$d_6$) spectrum of **3**

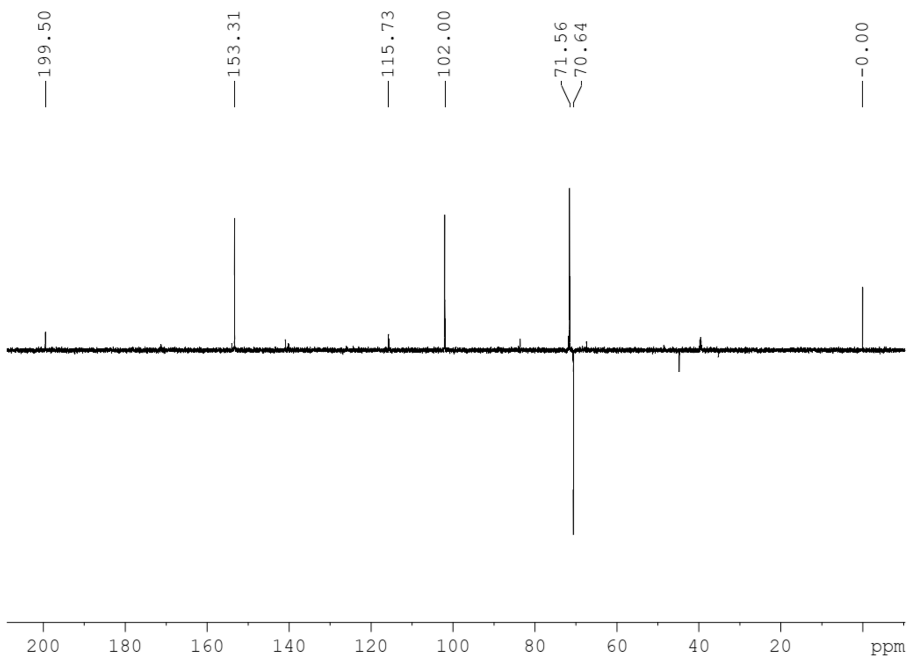


**Figure S14.** DEPT (125 MHz, DMSO-$d_6$) spectrum of **3**

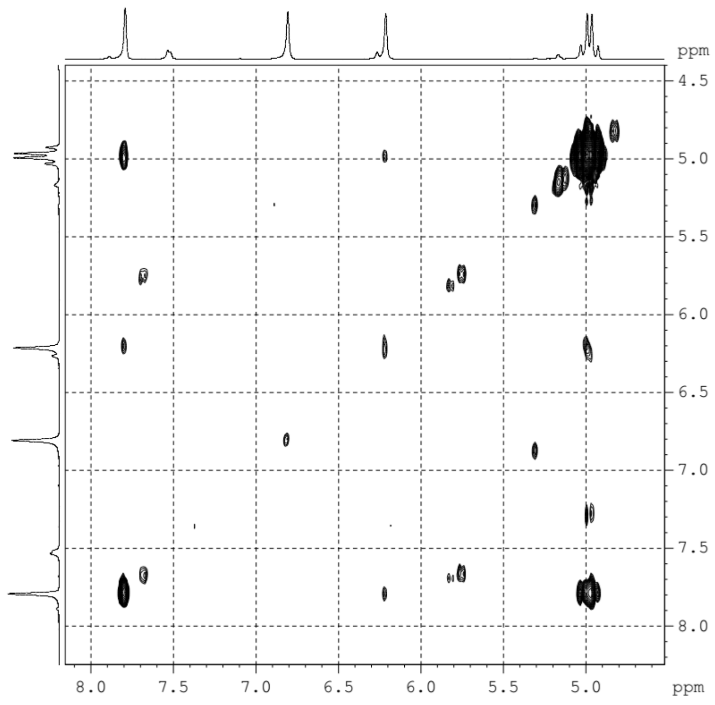


**Figure S15.** $^{1}$H $^{1}$H COSY (DMSO-$d_6$) spectrum of **3**

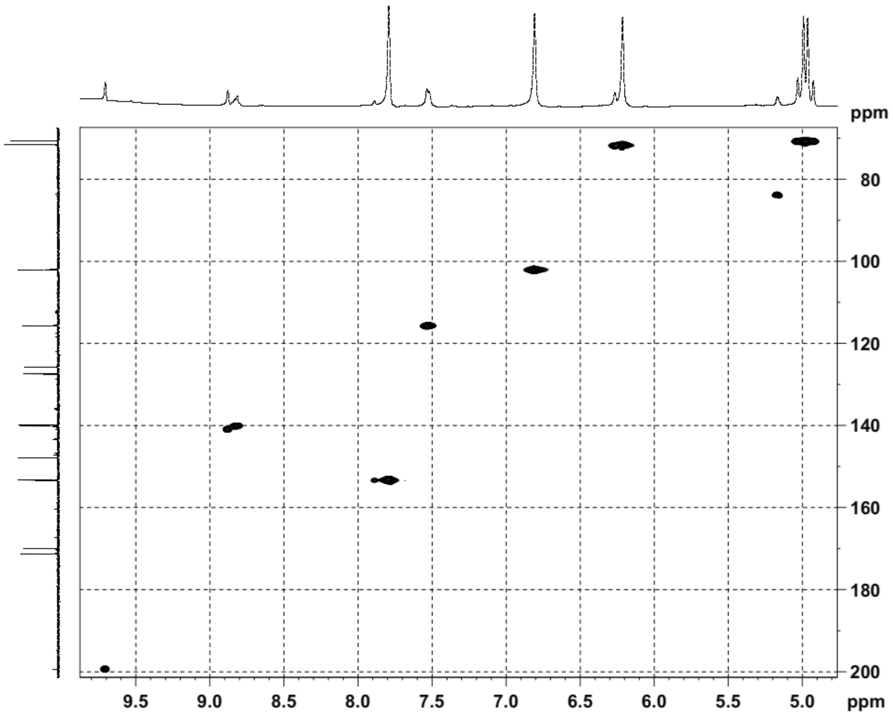


**Figure S16.** HSQC (DMSO-$d_6$) spectrum of **3**

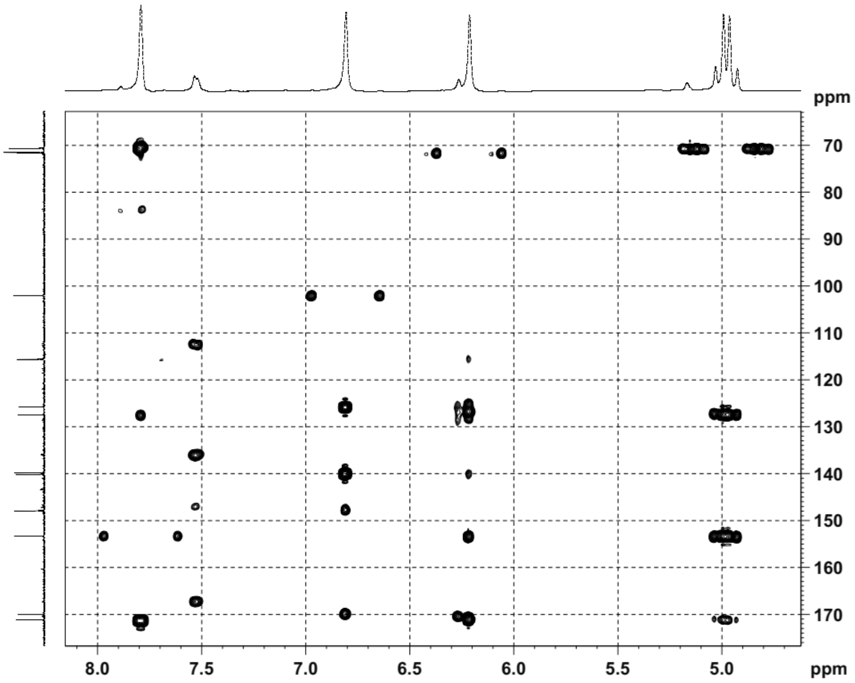


**Figure S17.** HMBC (DMSO-$d_6$) spectrum of **3**

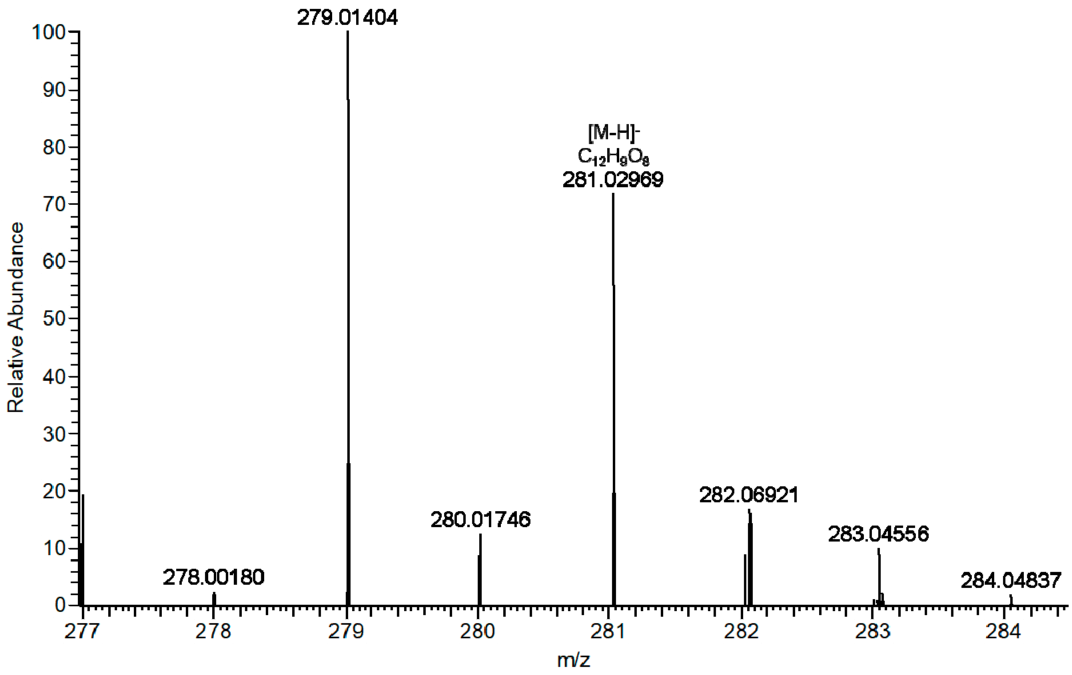


**Figure S18.** HRESIMS spectrum of **3**

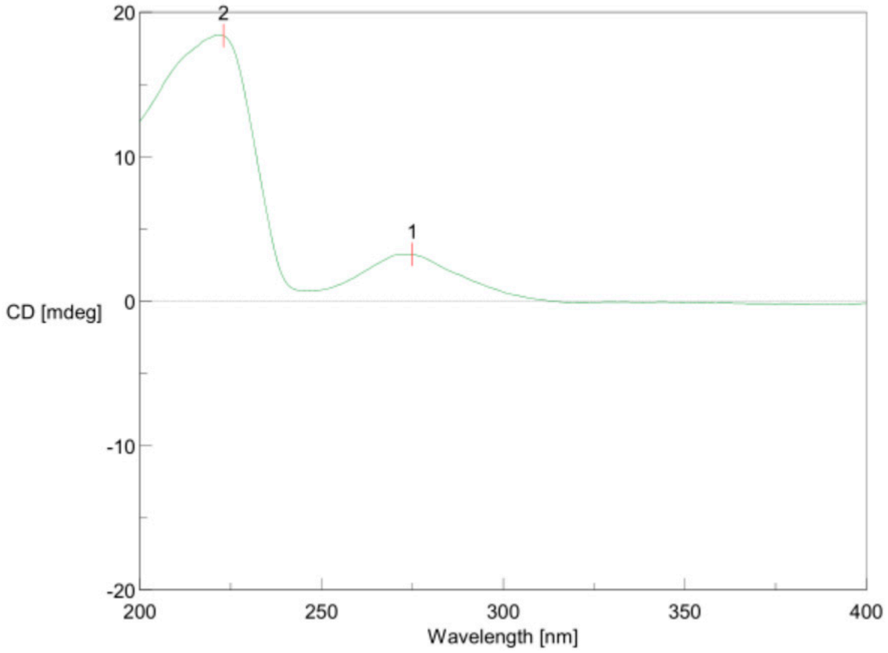


**Figure S19.** The experimental ECD spectrum of compound **3** in MeOH

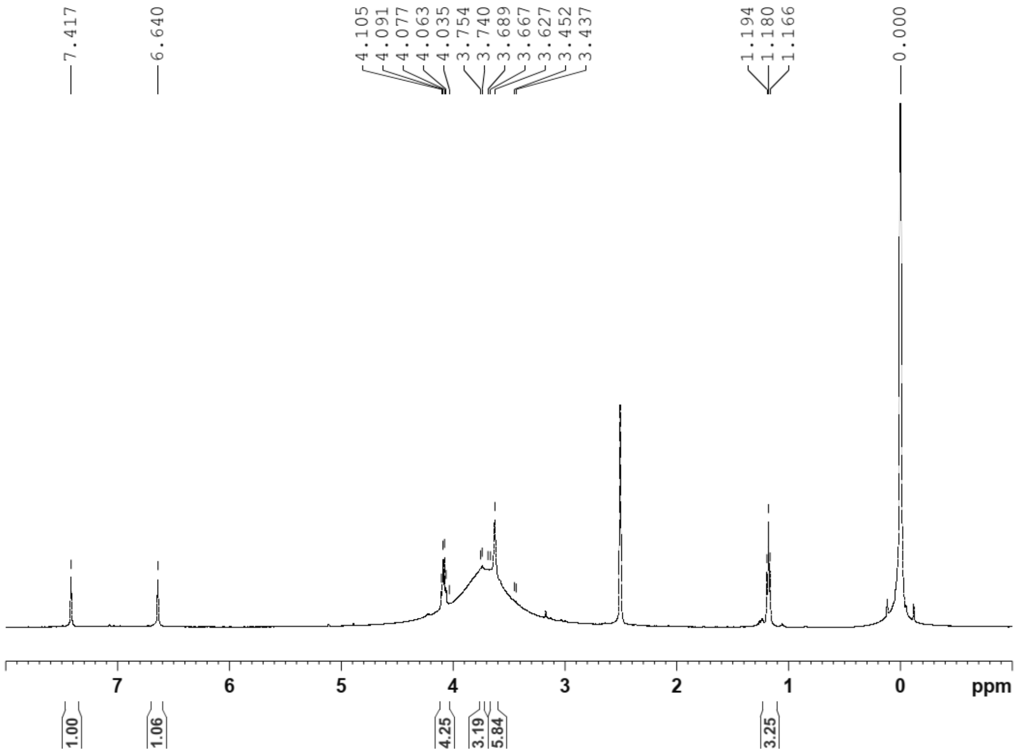


**Figure S20.** $^{1}H$ NMR (500 MHz, DMSO-$d_6$) spectrum of **4**

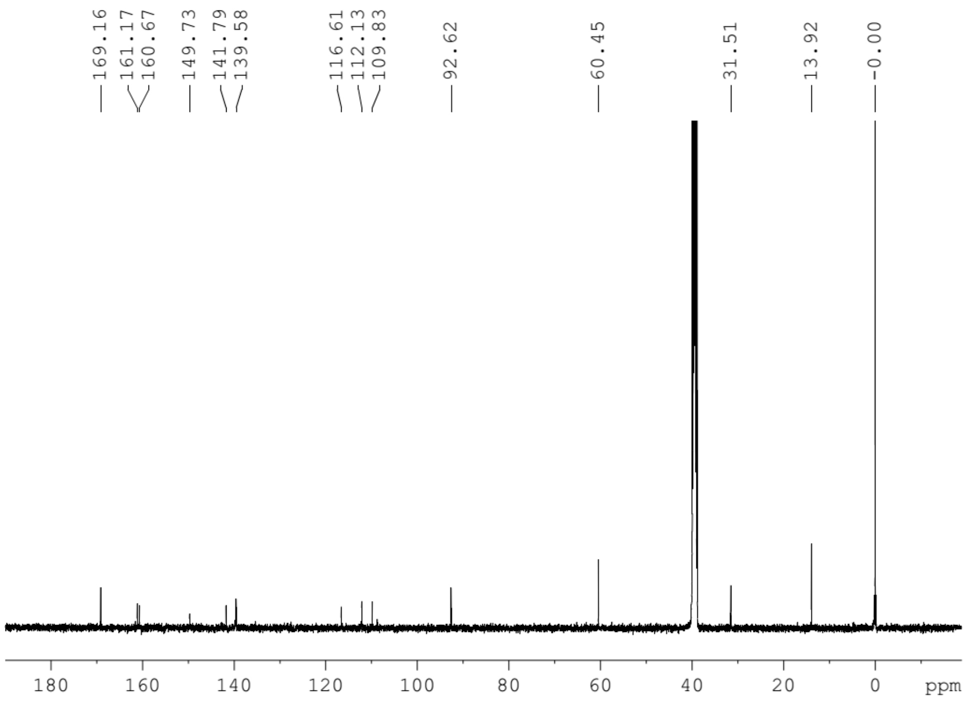


**Figure S21.** $^{13}C$ NMR (125 MHz, DMSO-$d_6$) spectrum of **4**

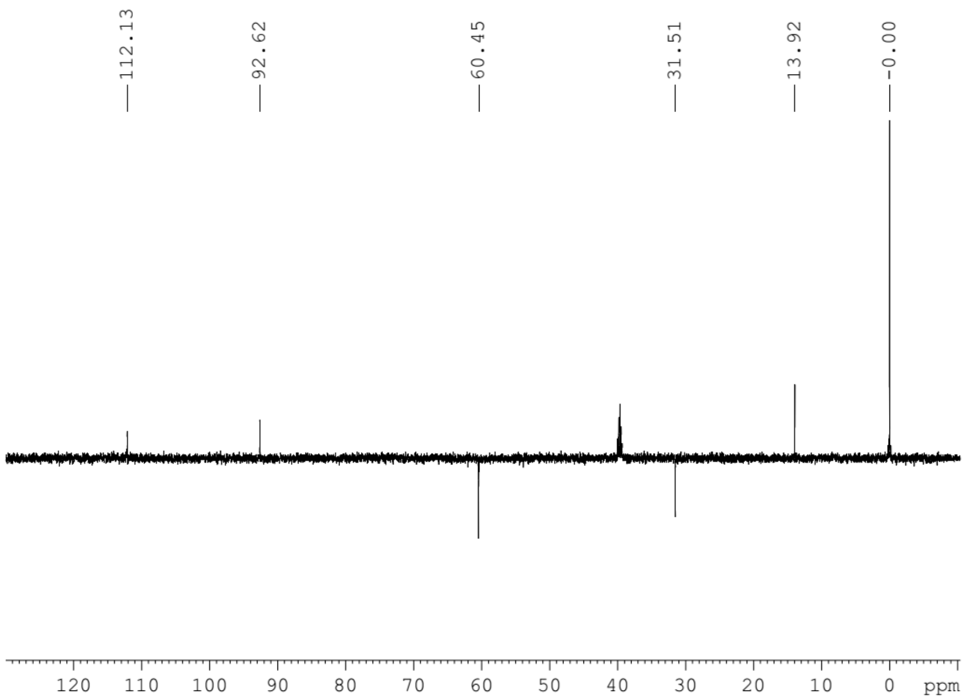


**Figure S22.** DEPT (125 MHz, DMSO-$d_6$) spectrum of **4**

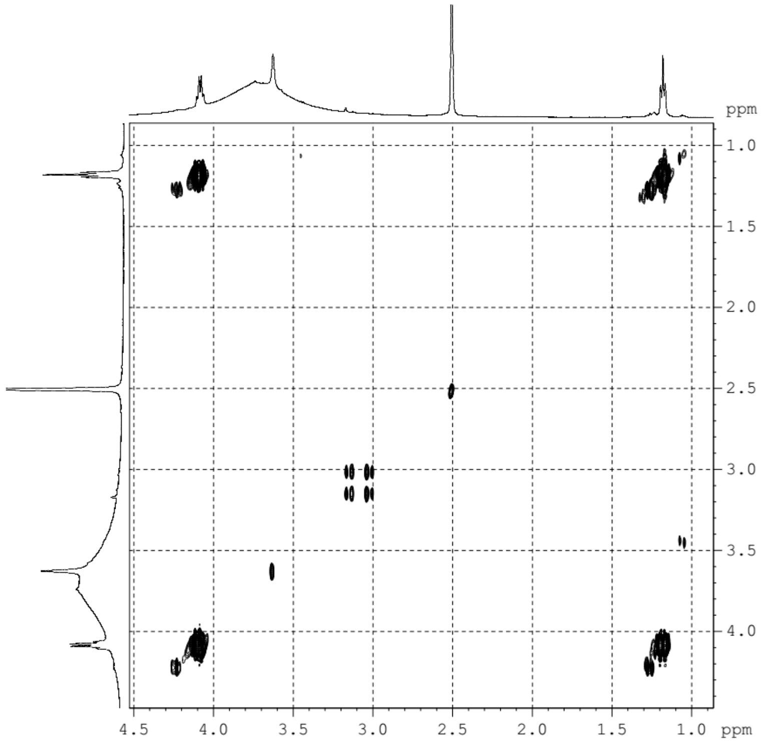


**Figure S23.** $^1$H $^1$H COSY (DMSO-$d_6$) spectrum of **4**

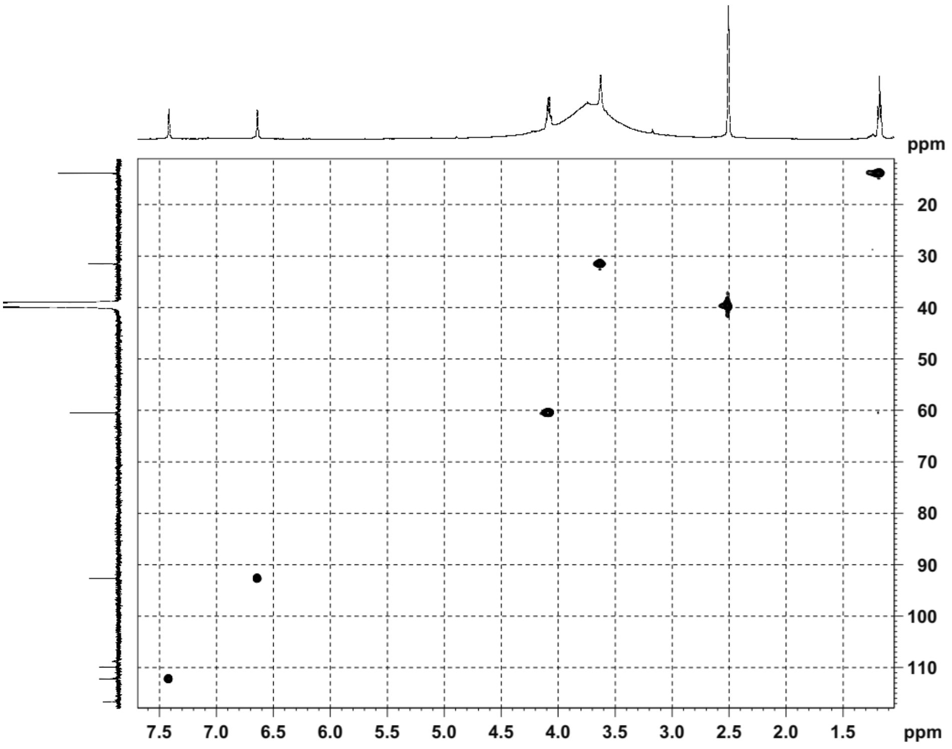


**Figure S24.** HSQC (DMSO-*d*6) spectrum of **4**

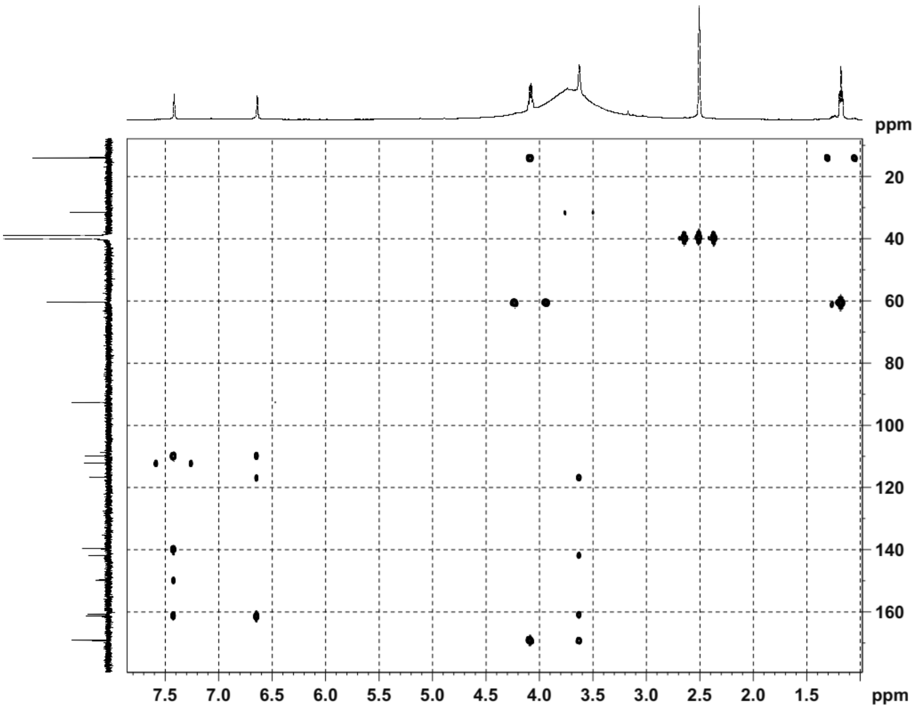


**Figure S25.** HMBC (DMSO-*d*6) spectrum of **4**

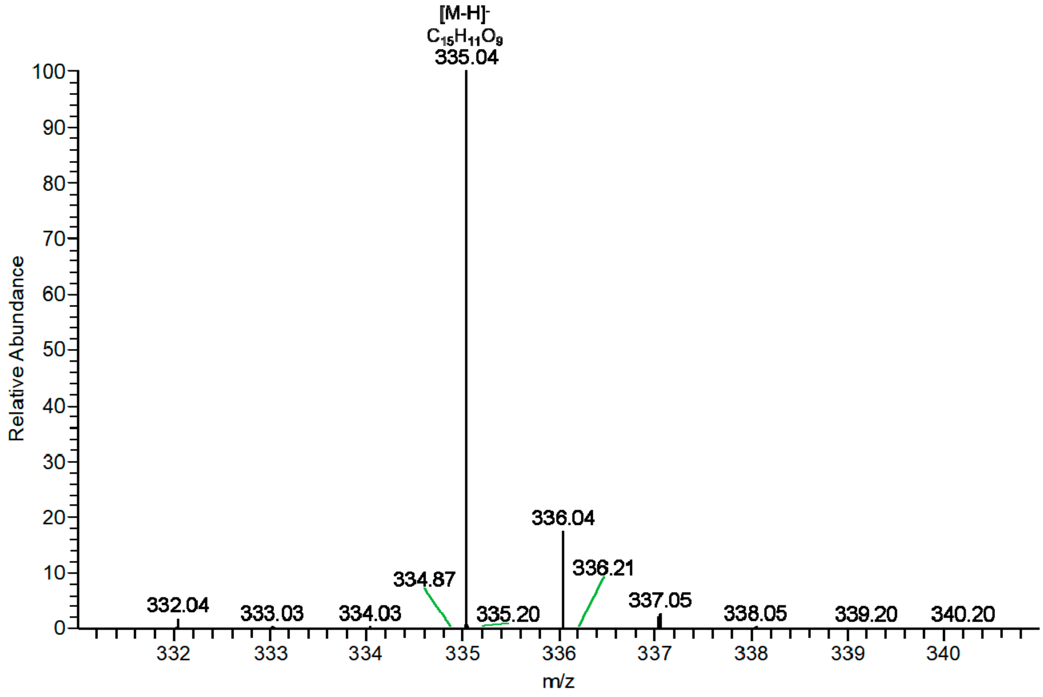


**Figure S26.** HRESIMS spectrum of **4**

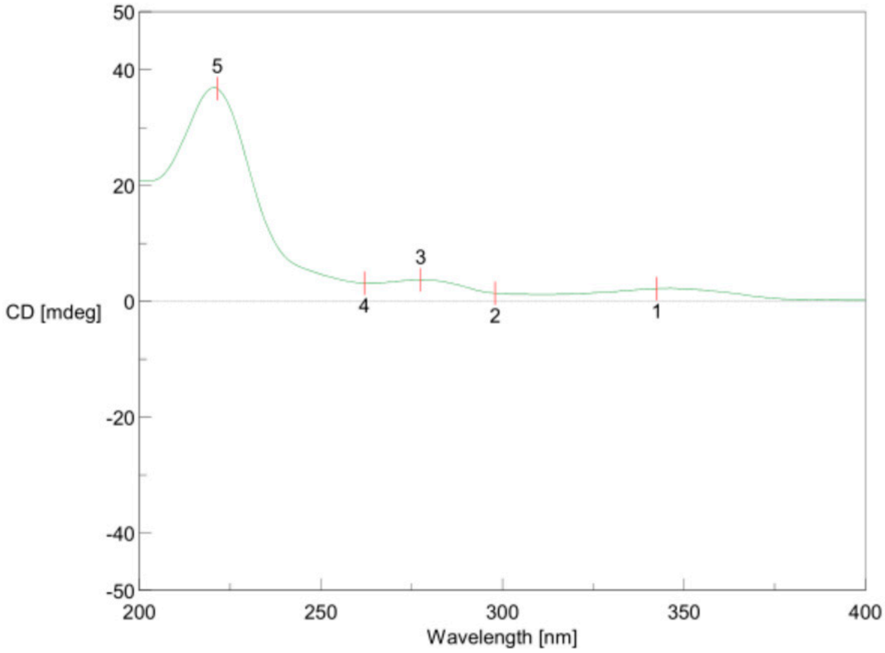


**Figure S27.** The experimental ECD spectrum of compound **4** in MeOH

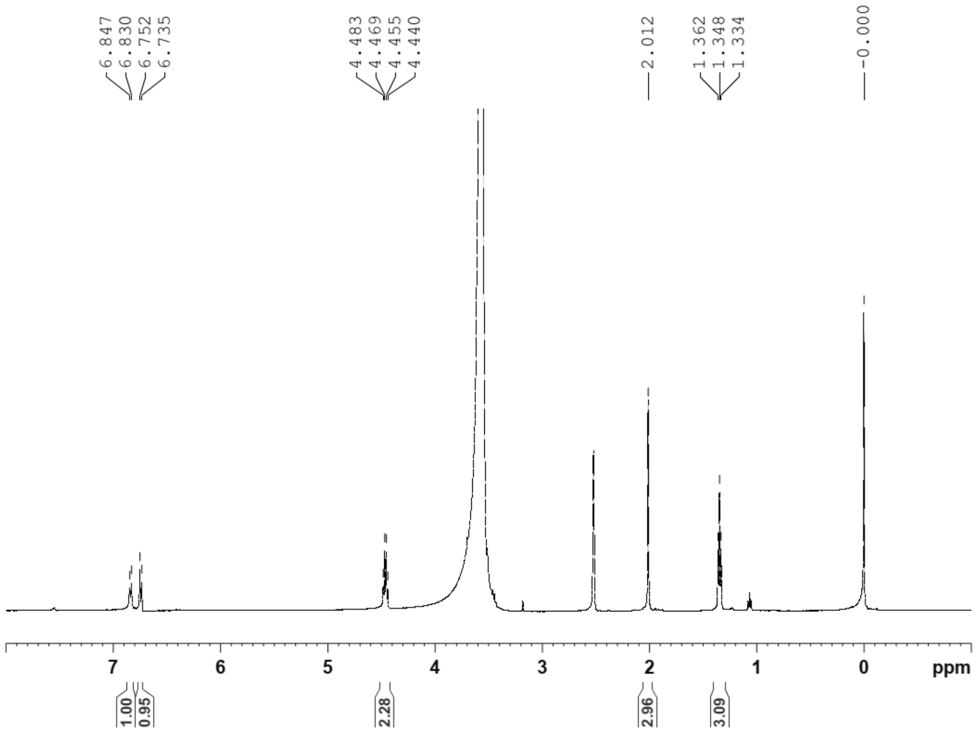


**Figure S28.** $^{1}H$ NMR (500 MHz, DMSO-$d_6$) spectrum of **5**

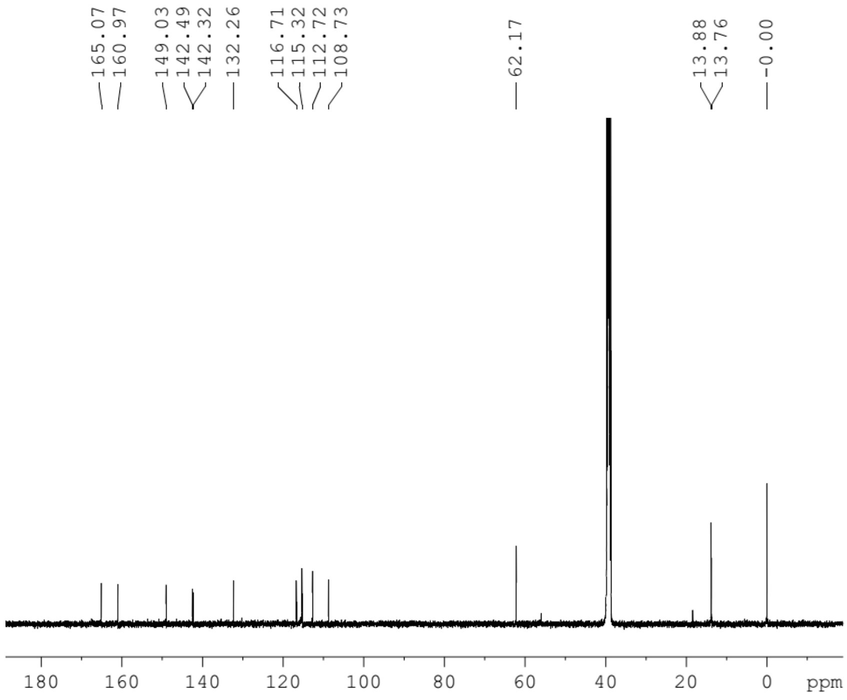


**Figure S29.** $^{13}C$ NMR (125 MHz, DMSO-$d_6$) spectrum of **5**

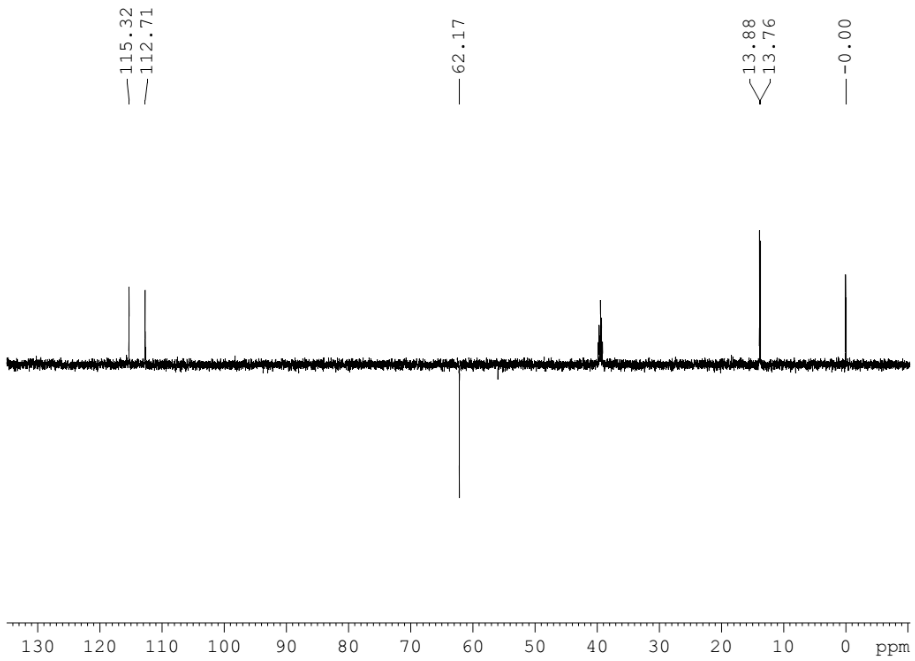


**Figure S30.** DEPT (125 MHz, DMSO-$d_6$) spectrum of **5**

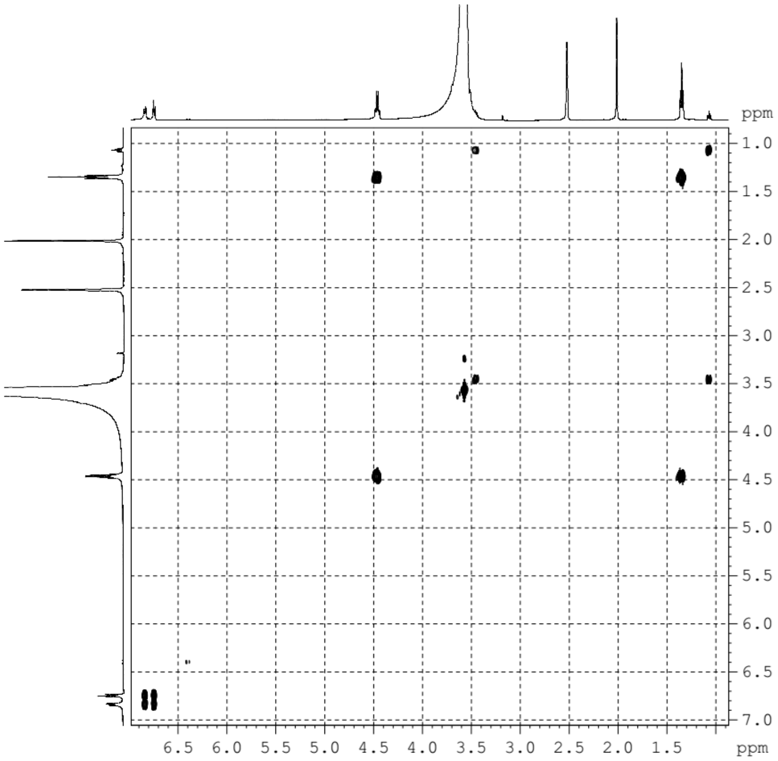


**Figure S31.** $^{1}H$ $^{1}H$ COSY (DMSO-$d_6$) spectrum of **5**

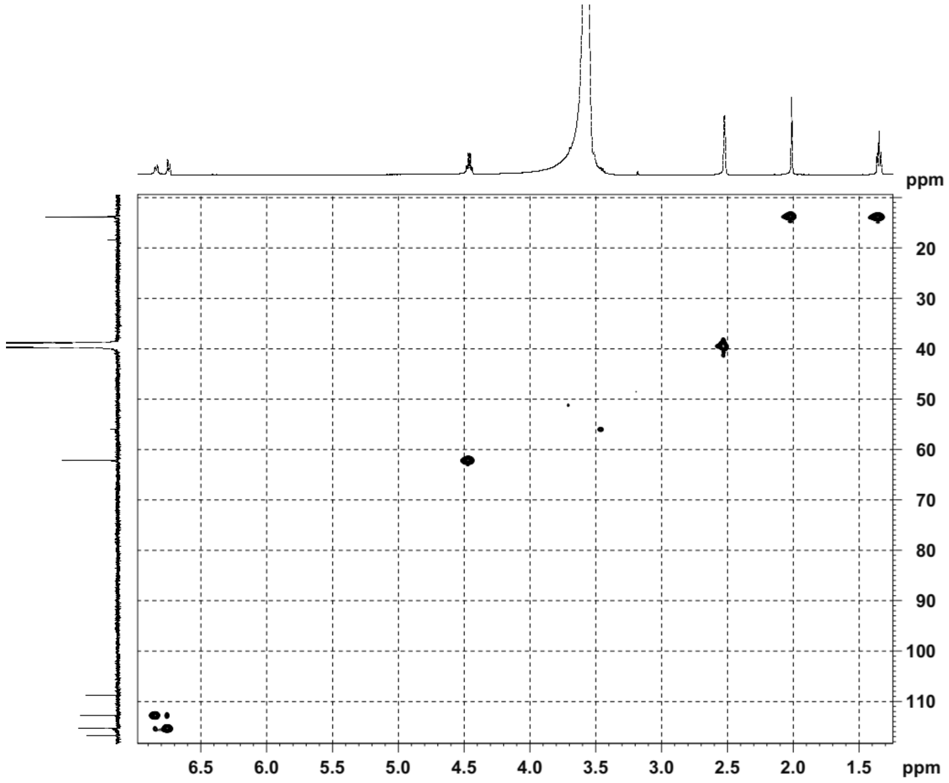


**Figure S32.** HSQC (DMSO-$d_6$) spectrum of **5**

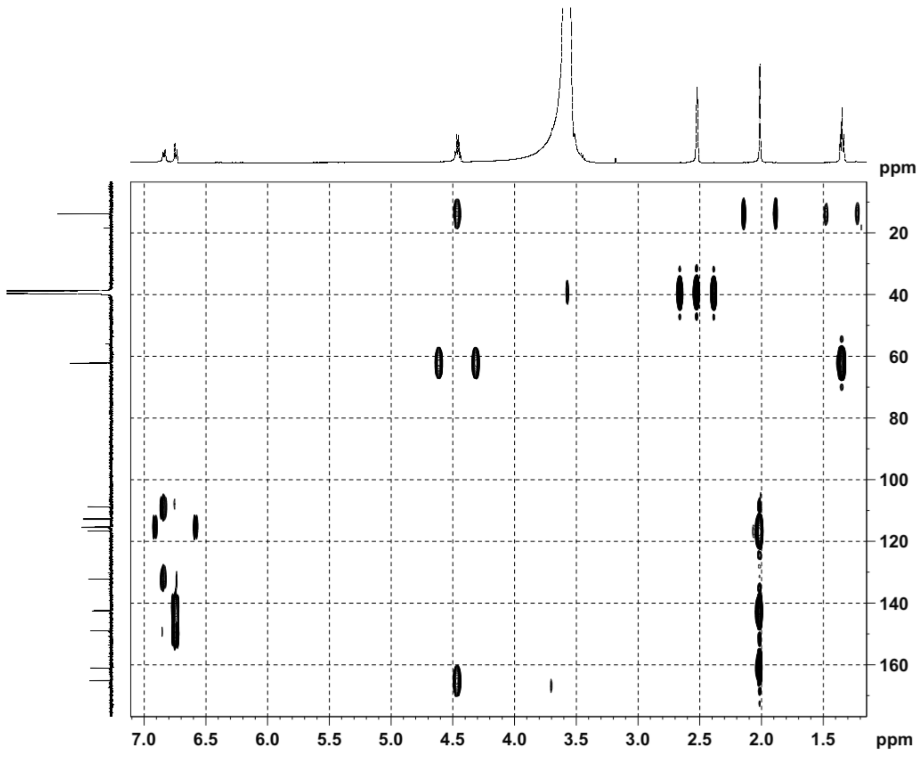


**Figure S33.** HMBC (DMSO-$d_6$) spectrum of **5**

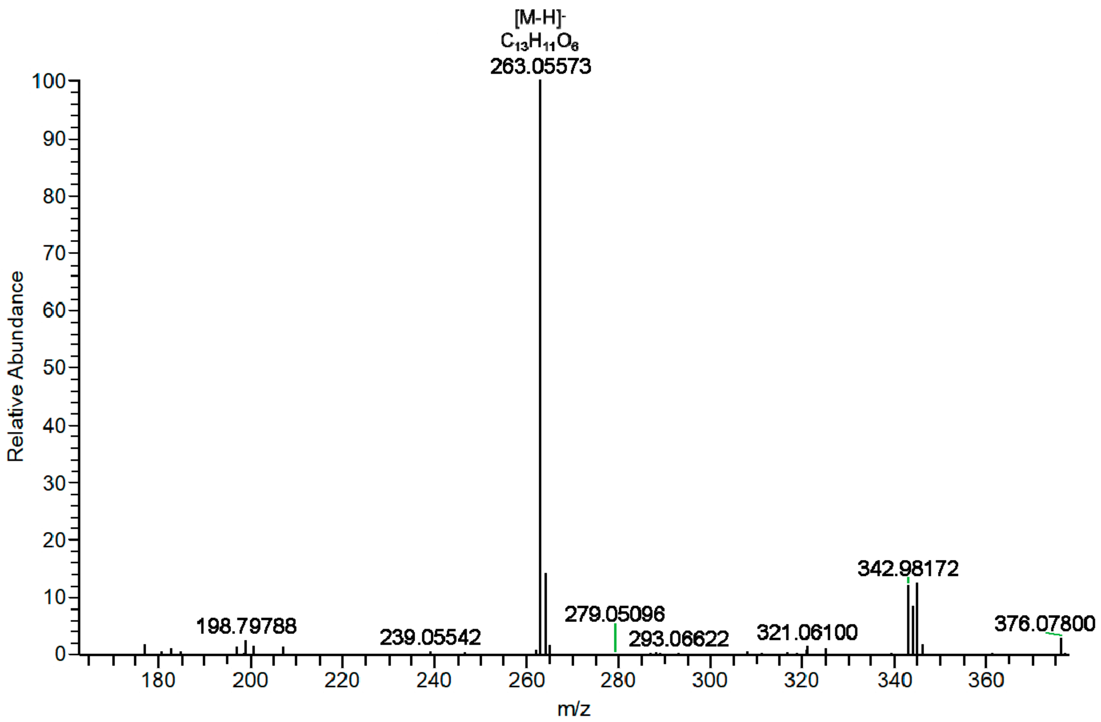


**Figure S34.** HRESIMS spectrum of **5**

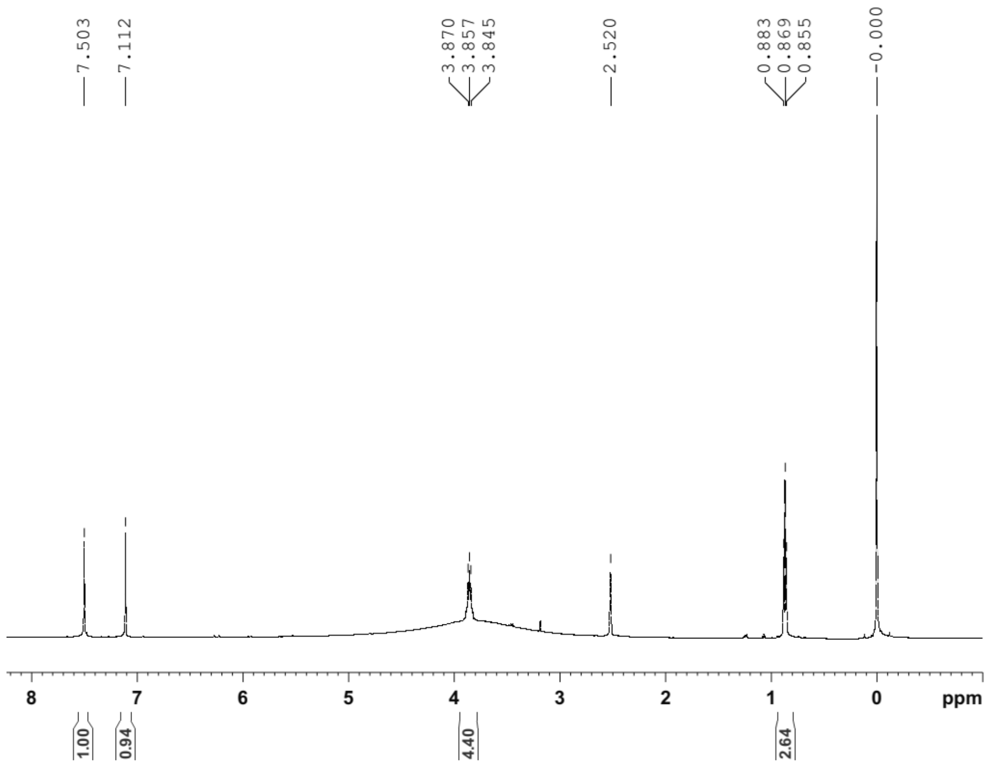


**Figure S35.** $^{1}H$ NMR (500 MHz, DMSO-$d_6$) spectrum of **6**

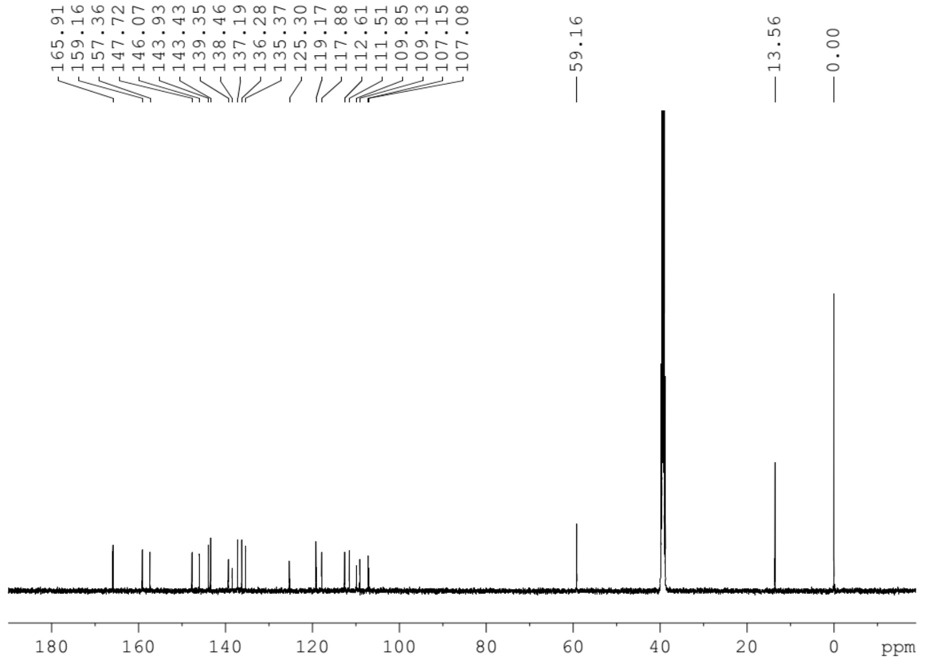


**Figure S36.** $^{13}C$ NMR (125 MHz, DMSO-$d_6$) spectrum of **6**

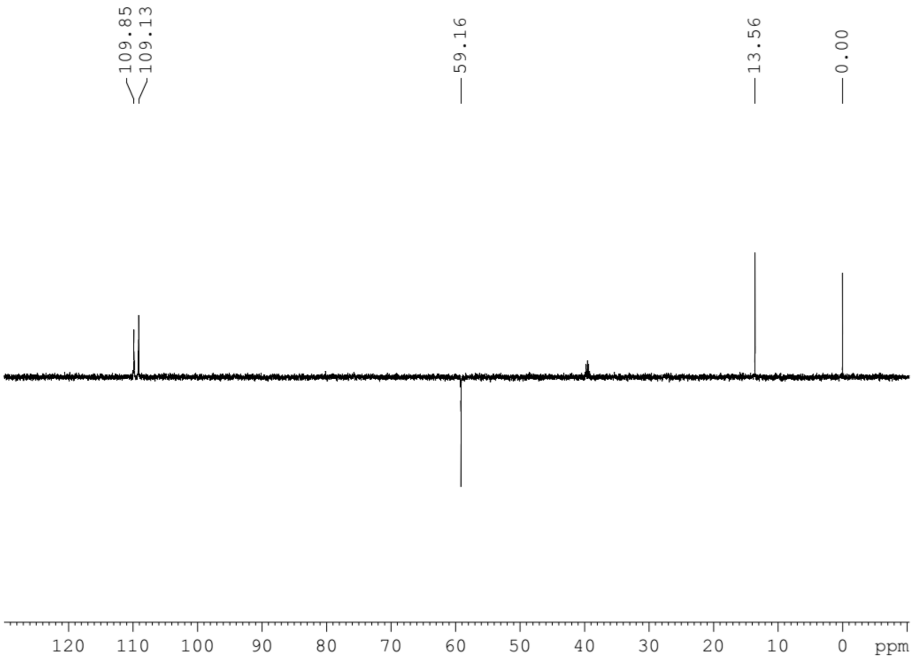


**Figure S37.** DEPT (125 MHz, DMSO-$d_6$) spectrum of **6**

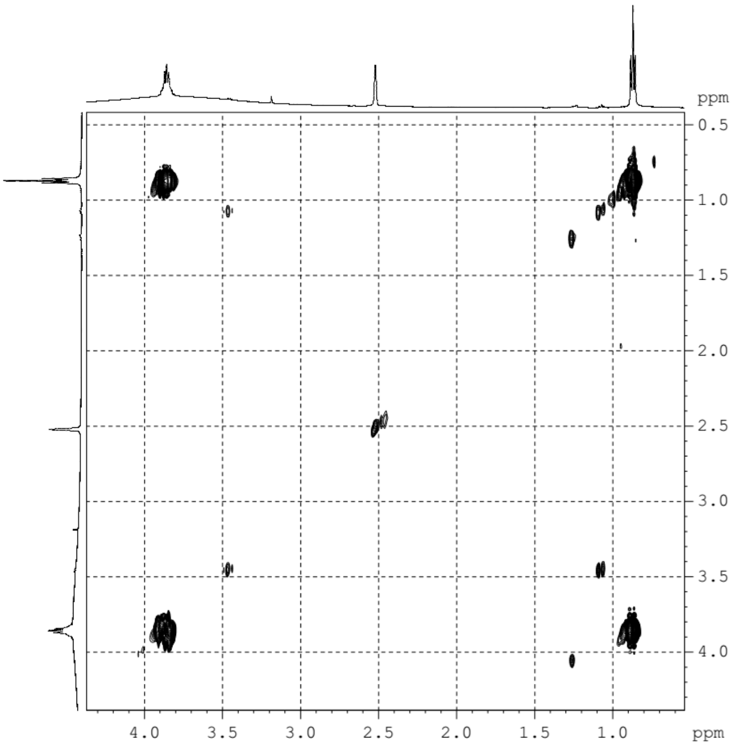


**Figure S38.** $^1H$ $^1H$ COSY (DMSO-$d_6$) spectrum of **6**

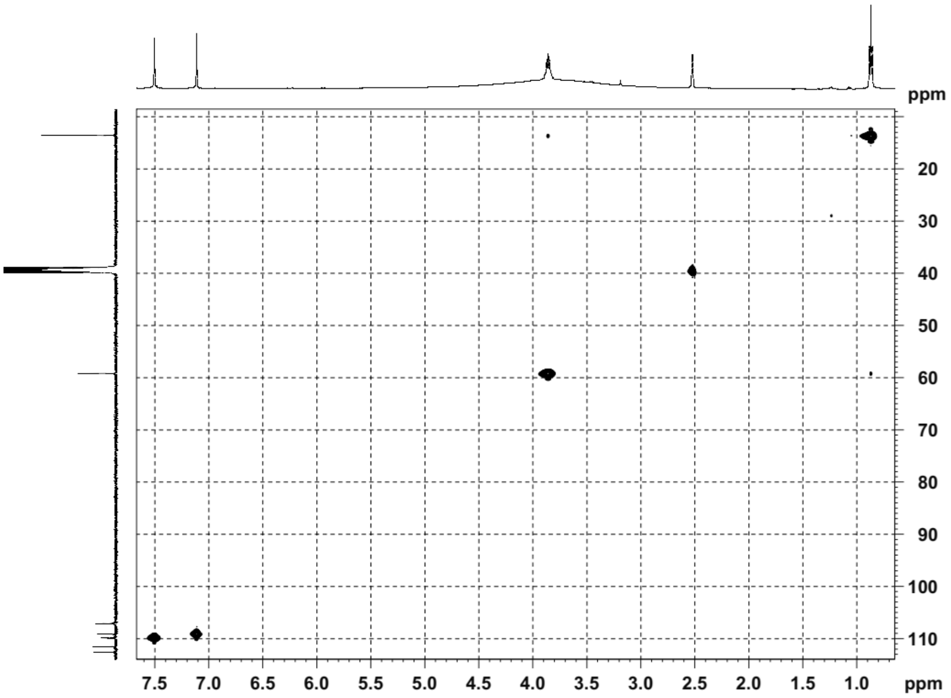


**Figure S39.** HSQC (DMSO-*d*6) spectrum of **6**

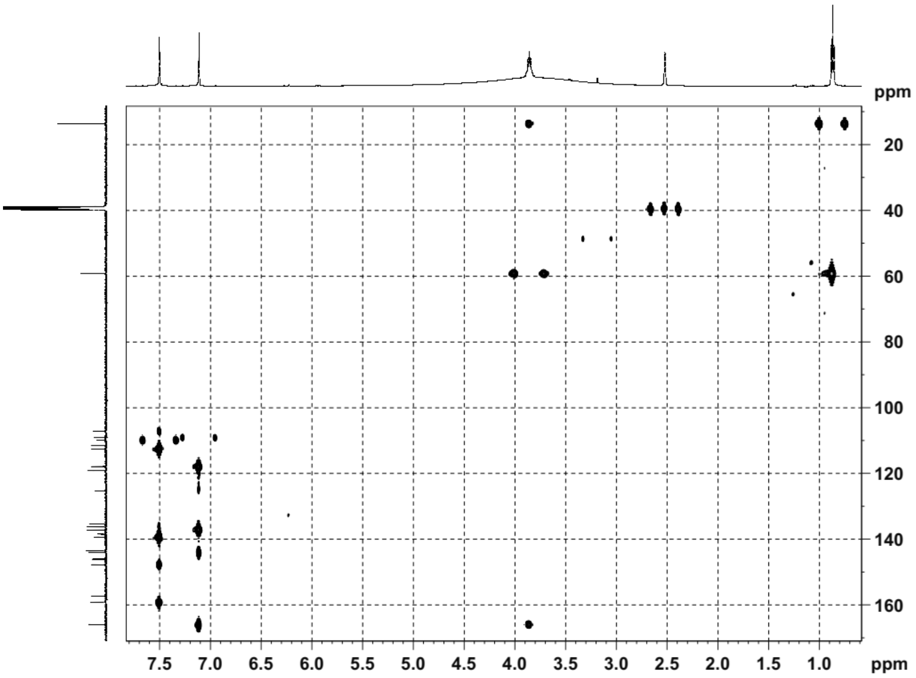


**Figure S40.** HMBC (DMSO-*d*6) spectrum of **6**

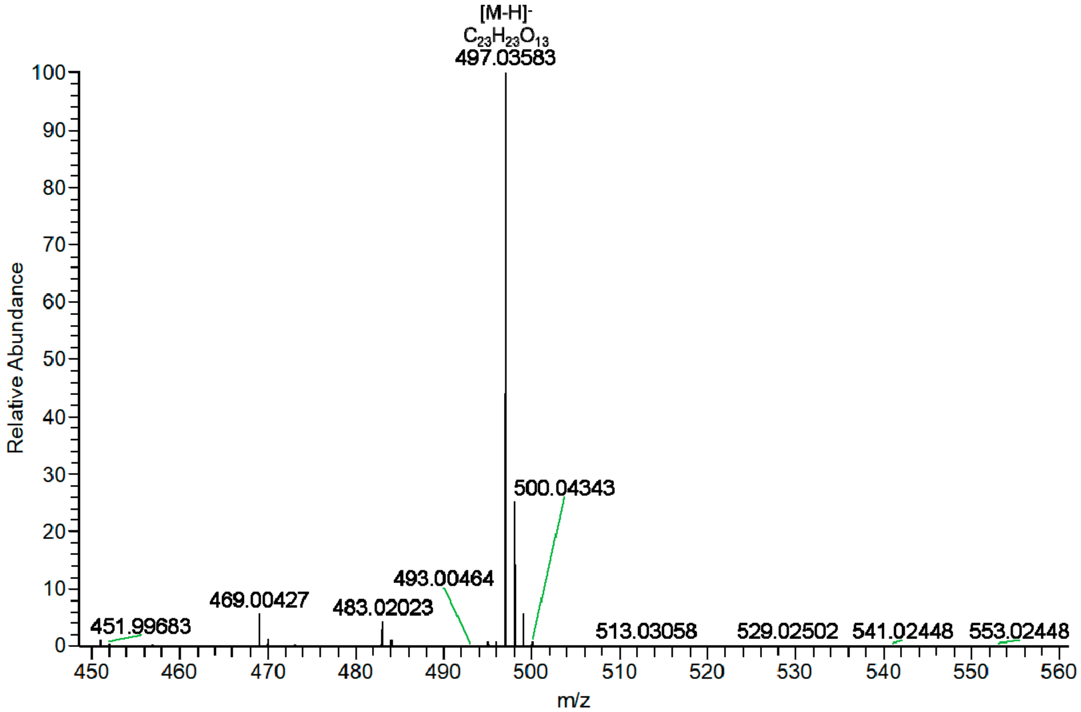


**Figure S41.** HRESIMS spectrum of **6**

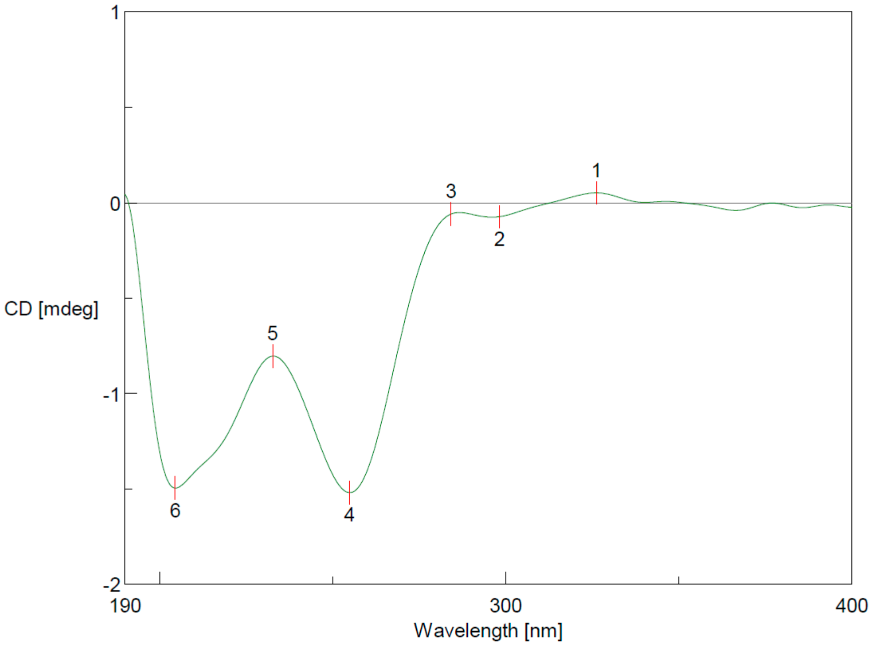


**Figure S42.** The experimental ECD spectrum of compound **6** in MeOH

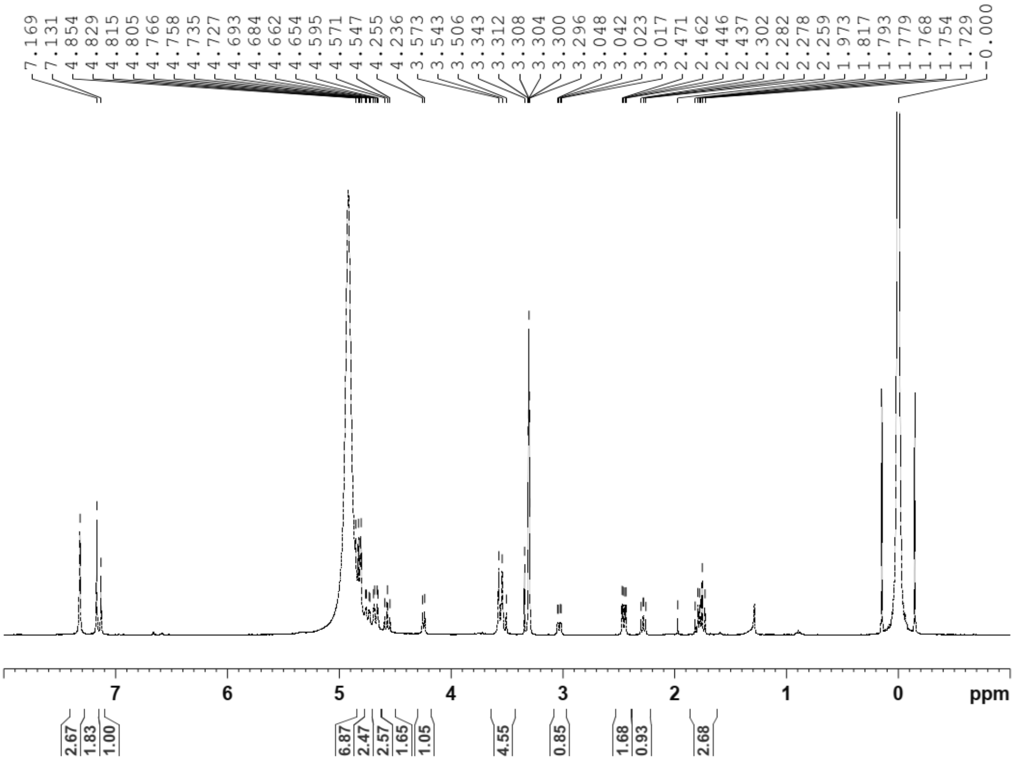

**Figure S43.** $^{1}H$ NMR (400 MHz, $CD_3OD$) spectrum of **7**

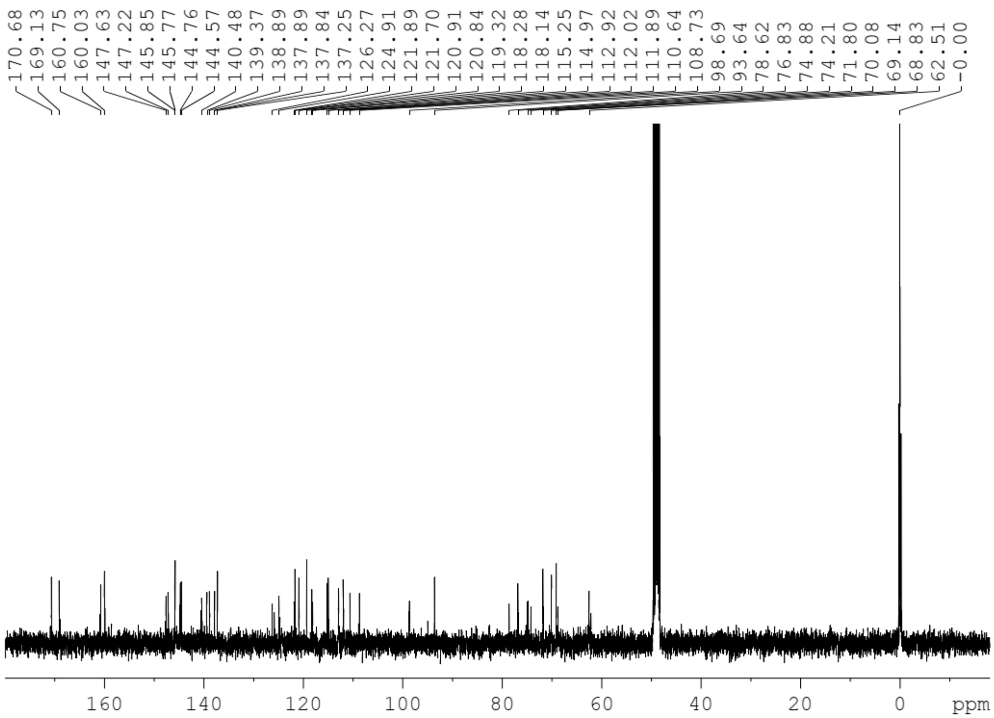

**Figure S44.** $^{13}C$ NMR (100 MHz, $CD_3OD$) spectrum of **7**

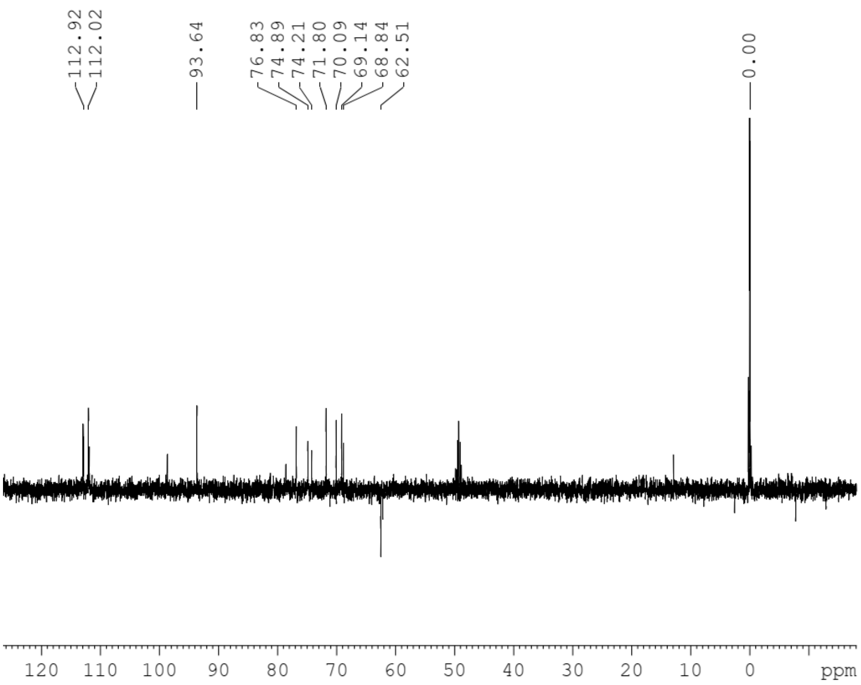


**Figure S45.** DEPT (100 MHz, $CD_3OD$) spectrum of **7**

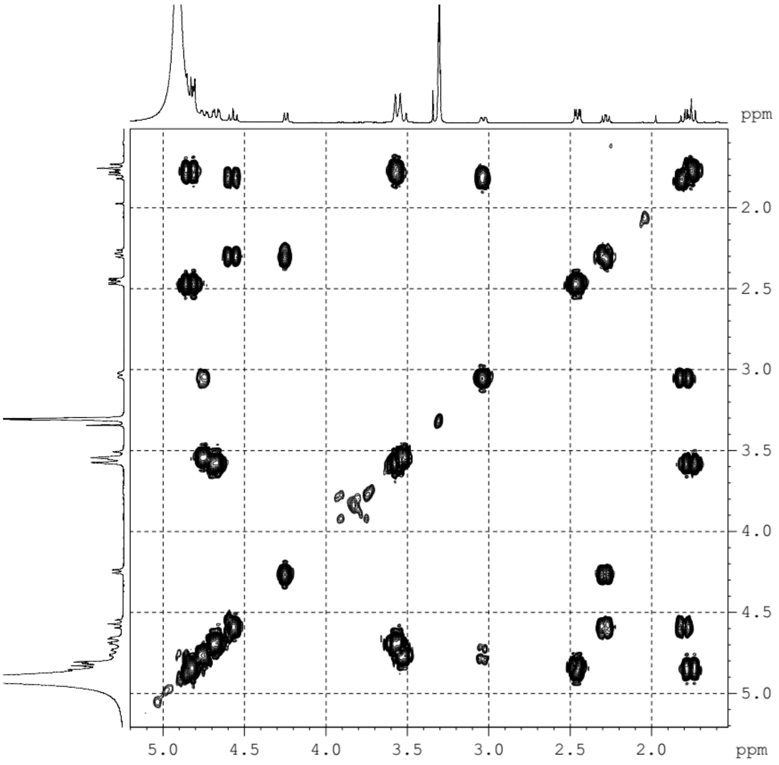


**Figure S46.** $^1H$ $^1H$ COSY ($CD_3OD$) spectrum of **7**

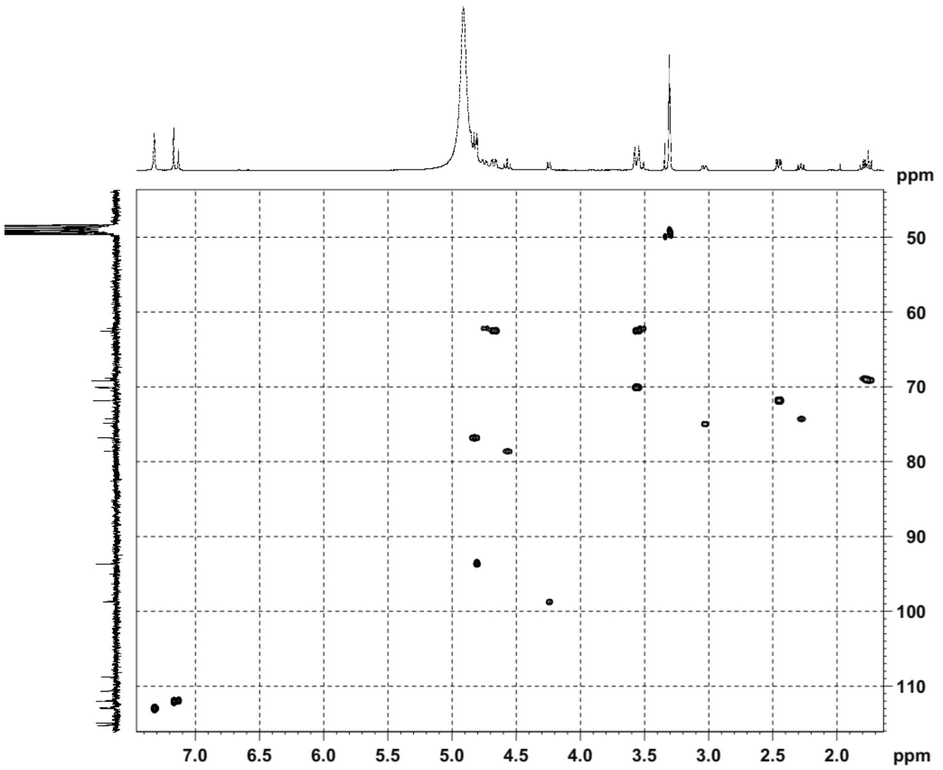


**Figure S47.** HSQC ($CD_3OD$) spectrum of **7**

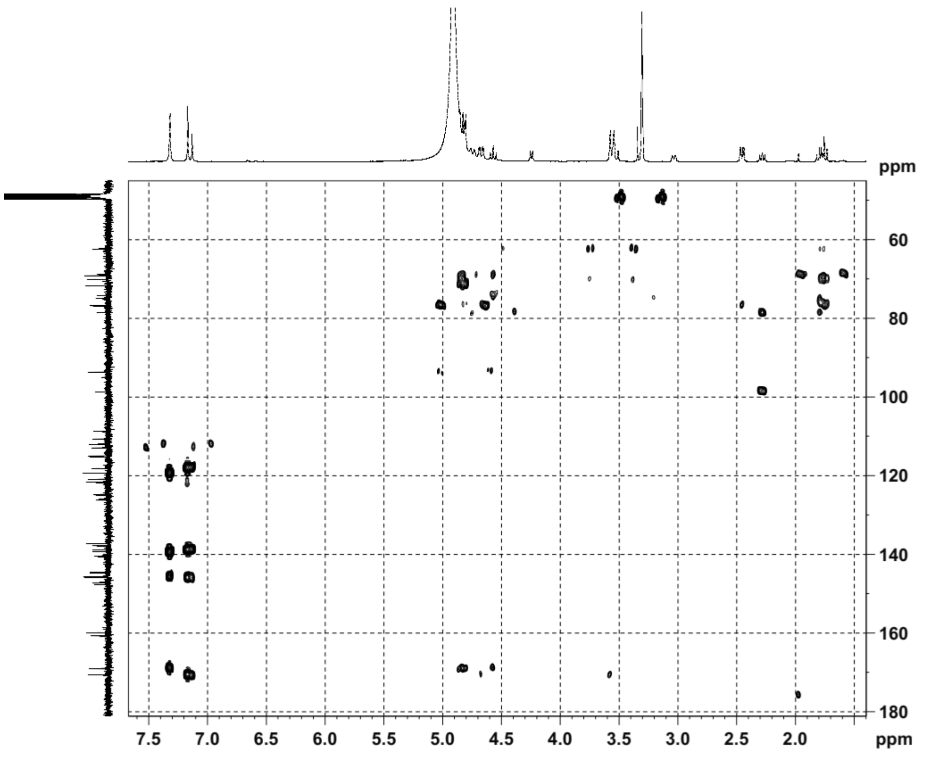


**Figure S48.** HMBC ($CD_3OD$) spectrum of **7**

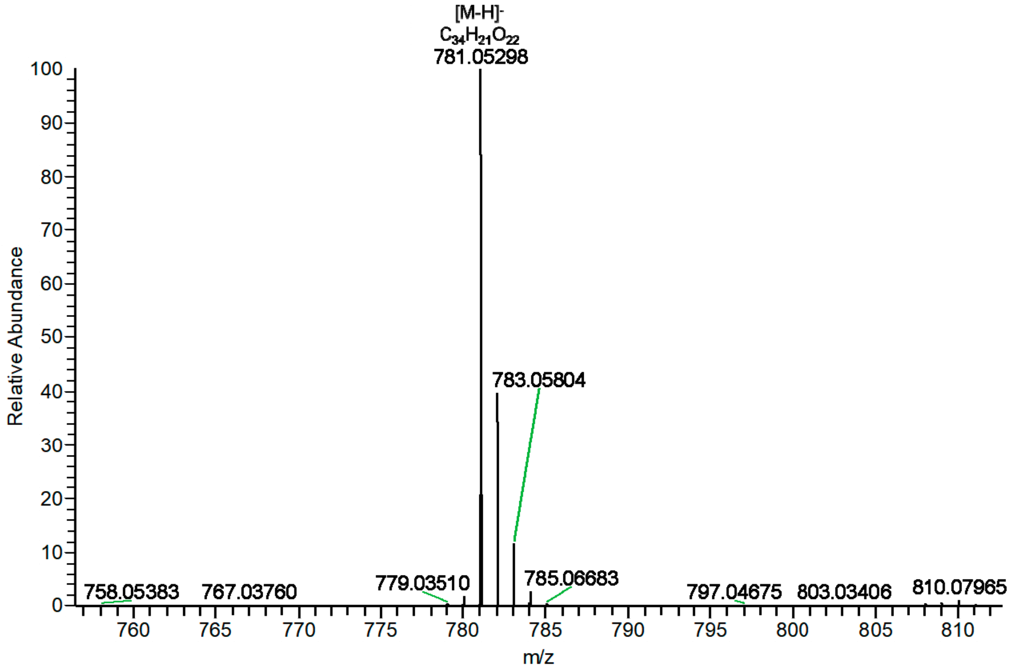


**Figure S49.** HRESIMS spectrum of **7**

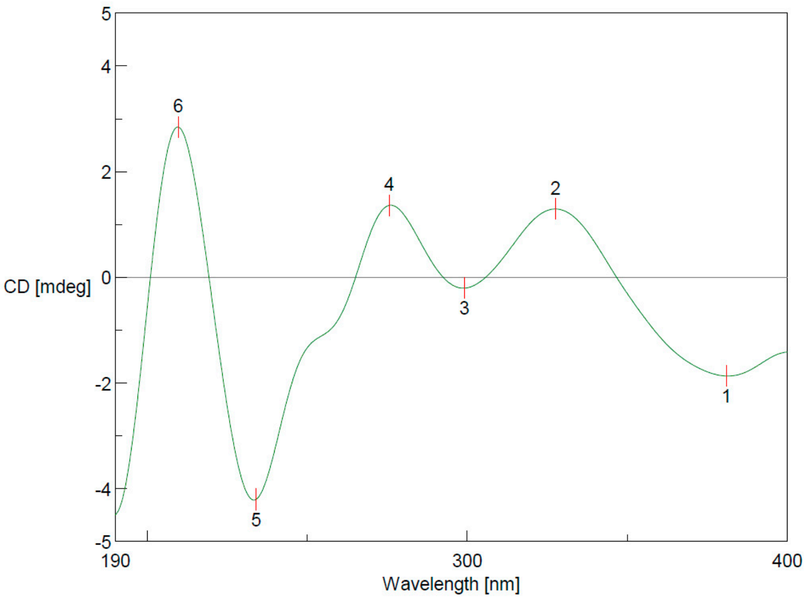


**Figure S50.** The experimental ECD spectrum of compound **7** in MeOH

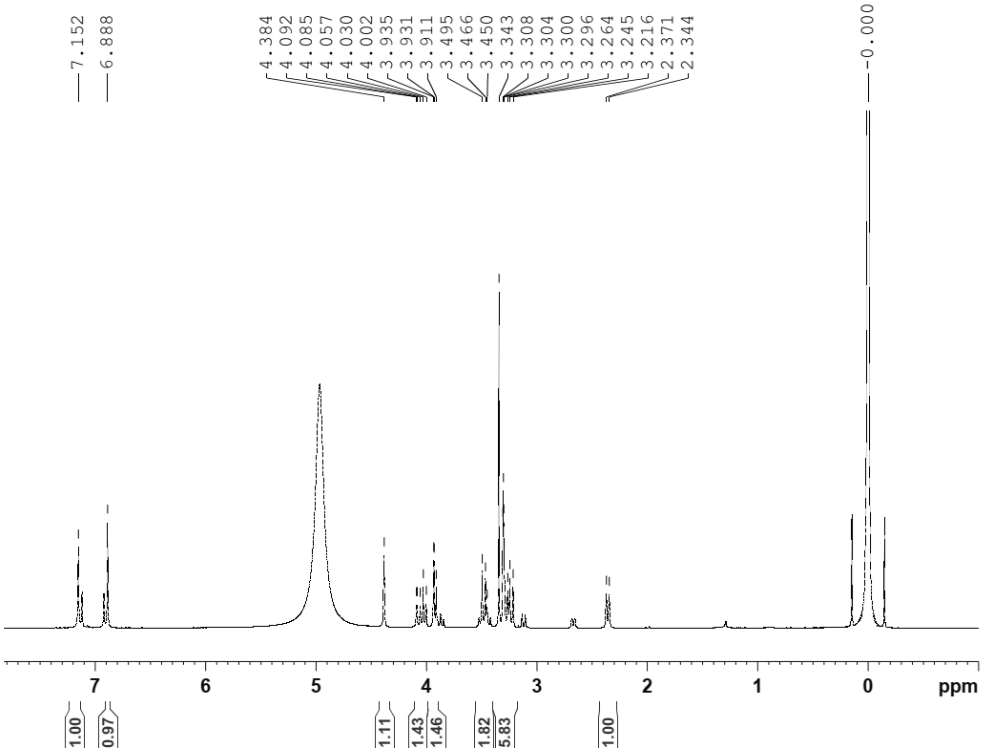


**Figure S51.** $^{1}H$ NMR (400 MHz, $CD_3OD$) spectrum of **8**

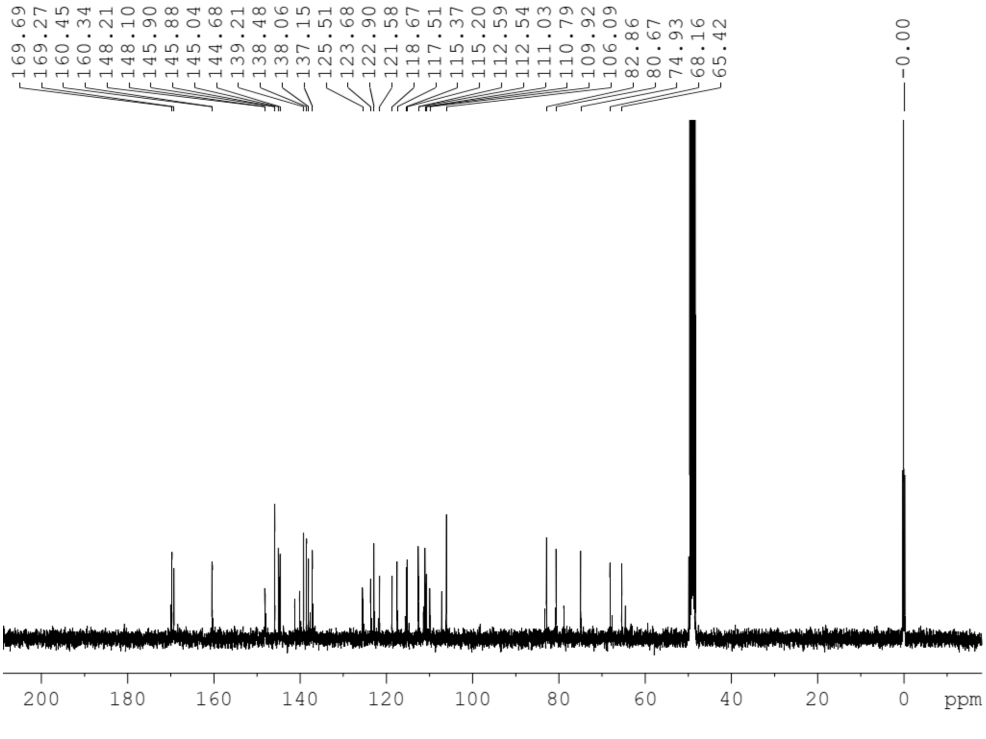


**Figure S52.** $^{13}C$ NMR (100 MHz, $CD_3OD$) spectrum of **8**

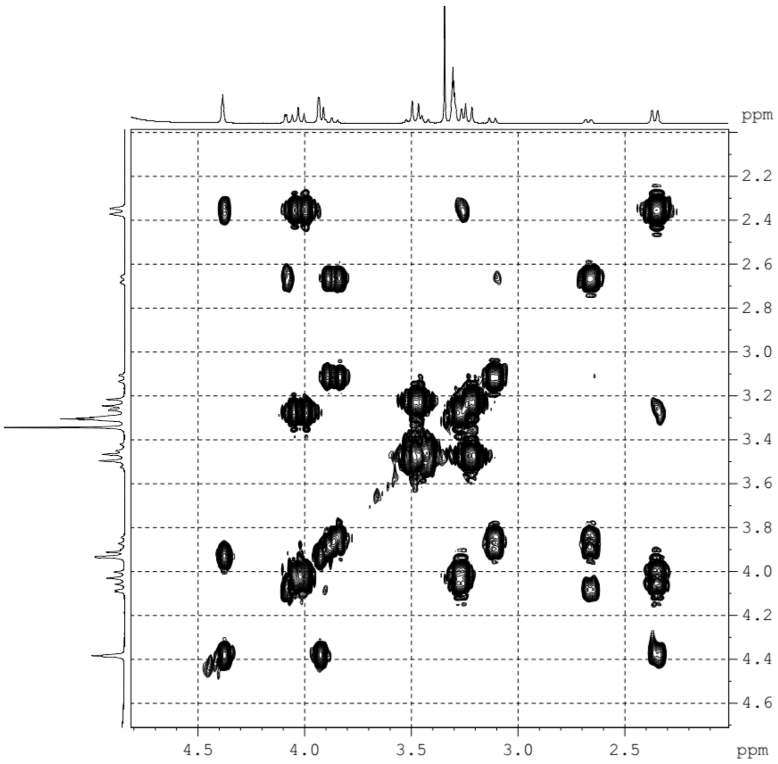


**Figure S53.** $^{1}$H $^{1}$H COSY ($CD_3OD$) spectrum of **8**

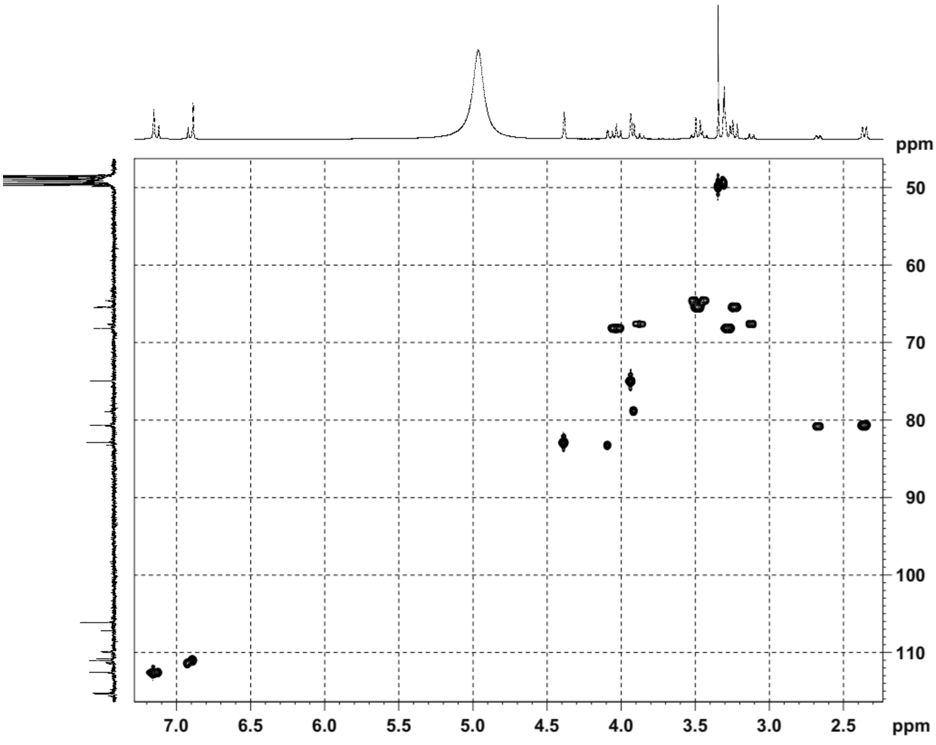


**Figure S54.** HSQC ($CD_3OD$) spectrum of **8**

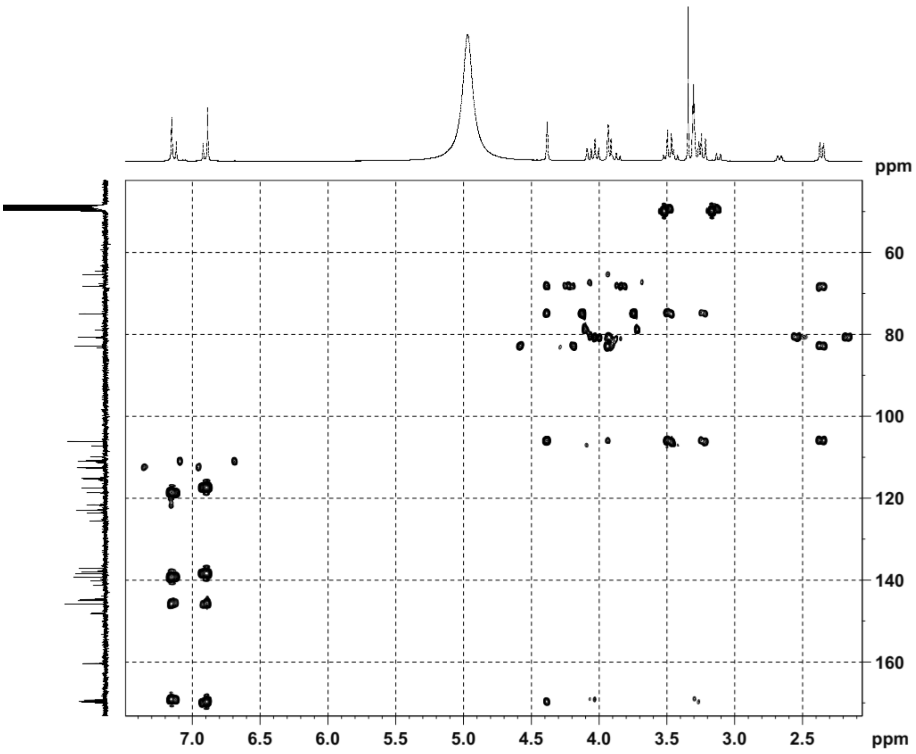


**Figure S55.** HMBC ($CD_3OD$) spectrum of **8**

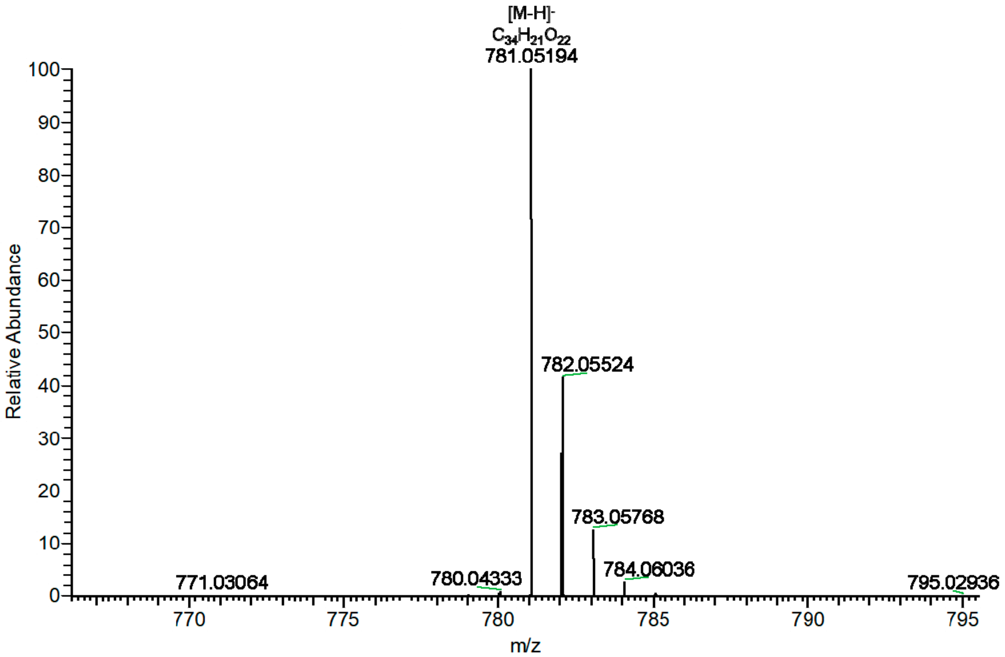


**Figure S56.** HRESIMS spectrum of **8**

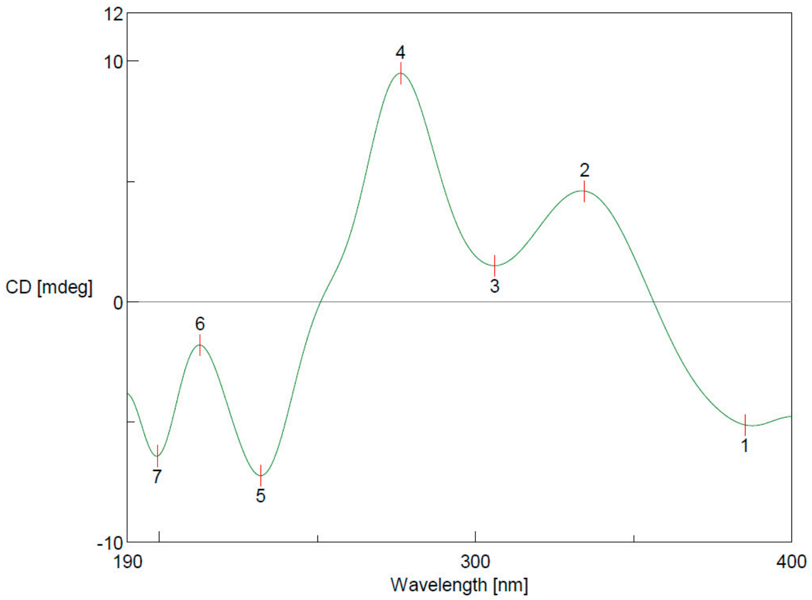


**Figure S57.** The experimental ECD spectrum of compound **8** in MeOH

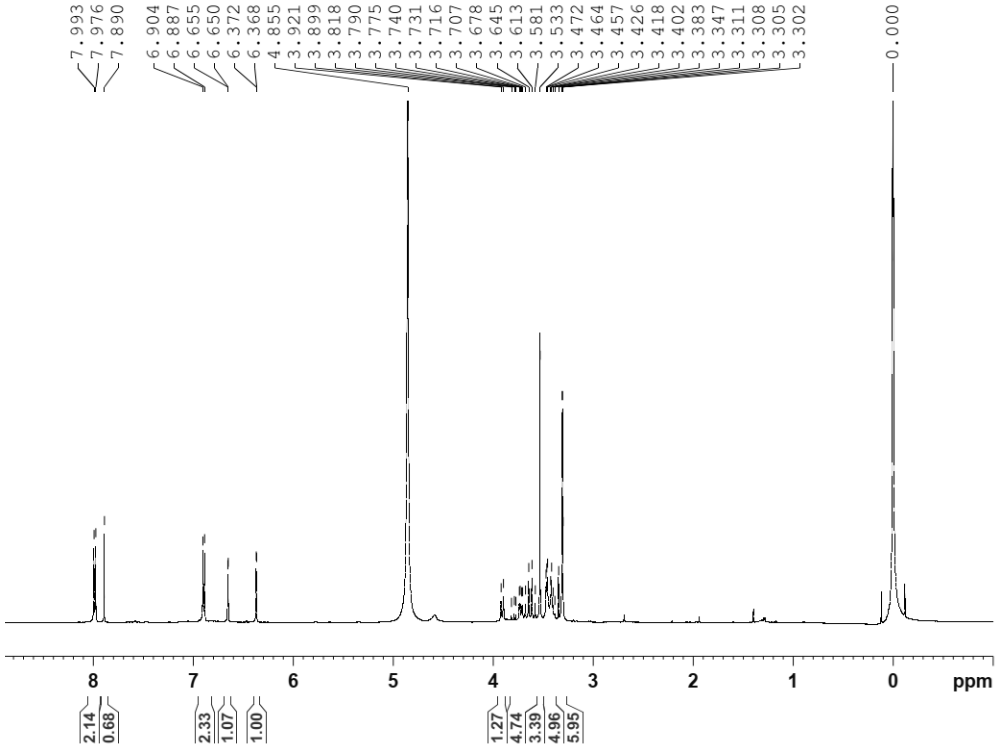


**Figure S58.** $^{1}H$ NMR (500 MHz, $CD_3OD$) spectrum of **9**

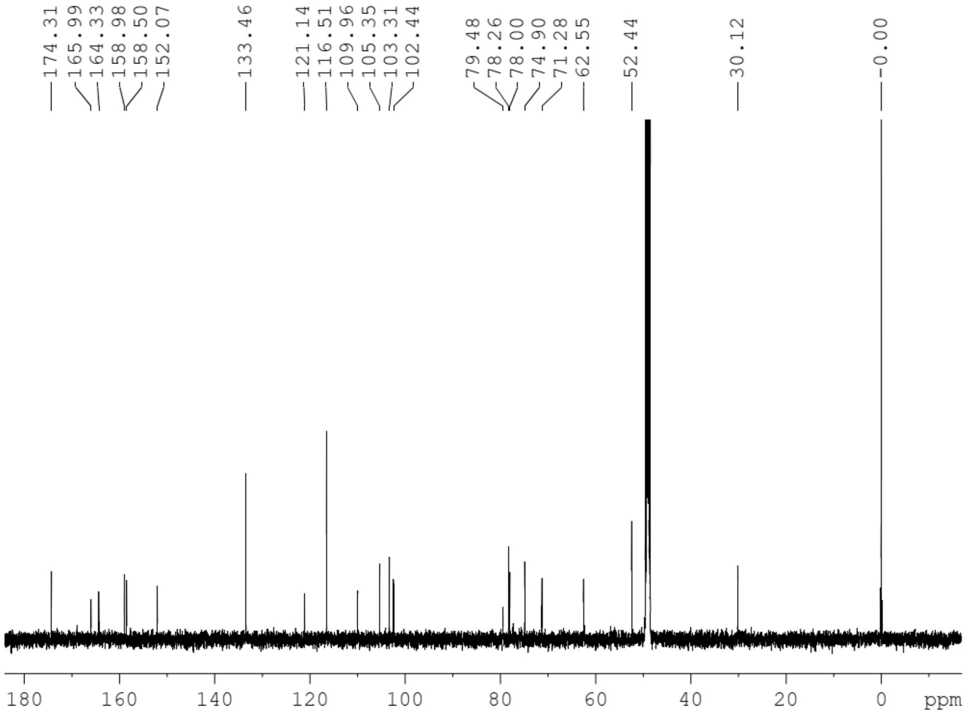


**Figure S59.** $^{13}C$ NMR (125 MHz, $CD_3OD$) spectrum of **9**

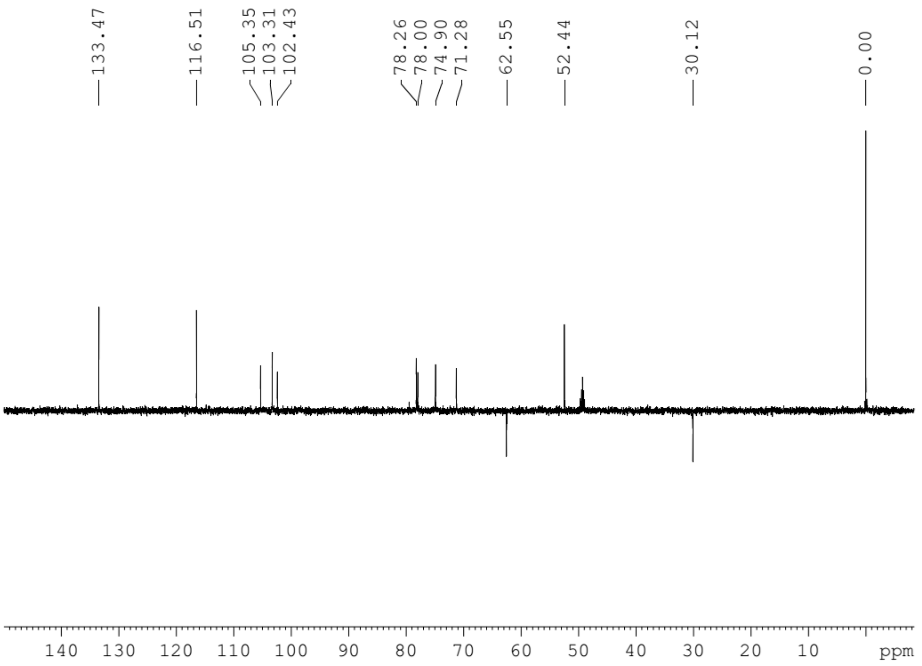


**Figure S60.** DEPT (125 MHz, $CD_3OD$) spectrum of **9**

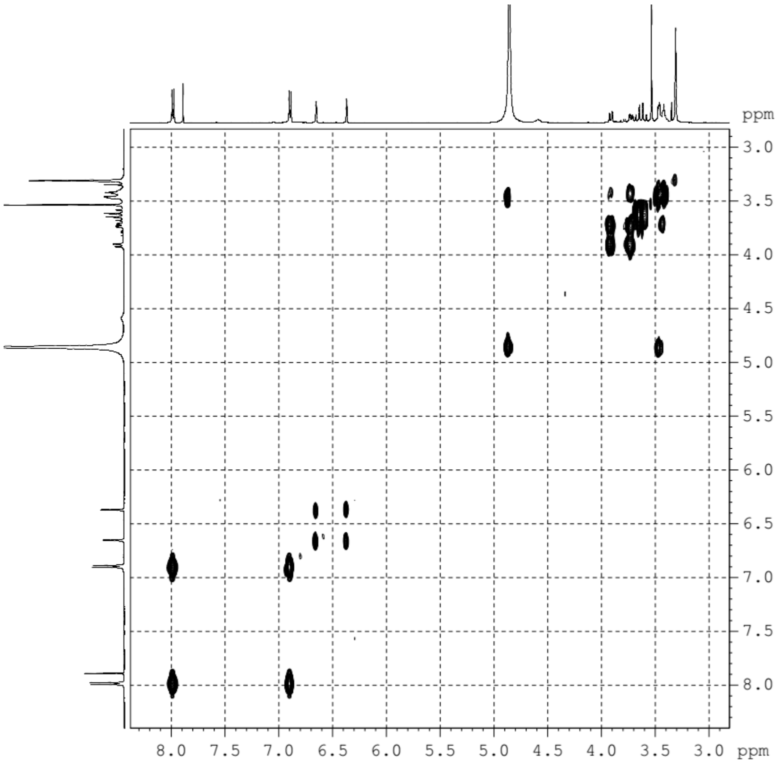


**Figure S61.** $^{1}$H $^{1}$H COSY ($CD_3OD$) spectrum of **9**

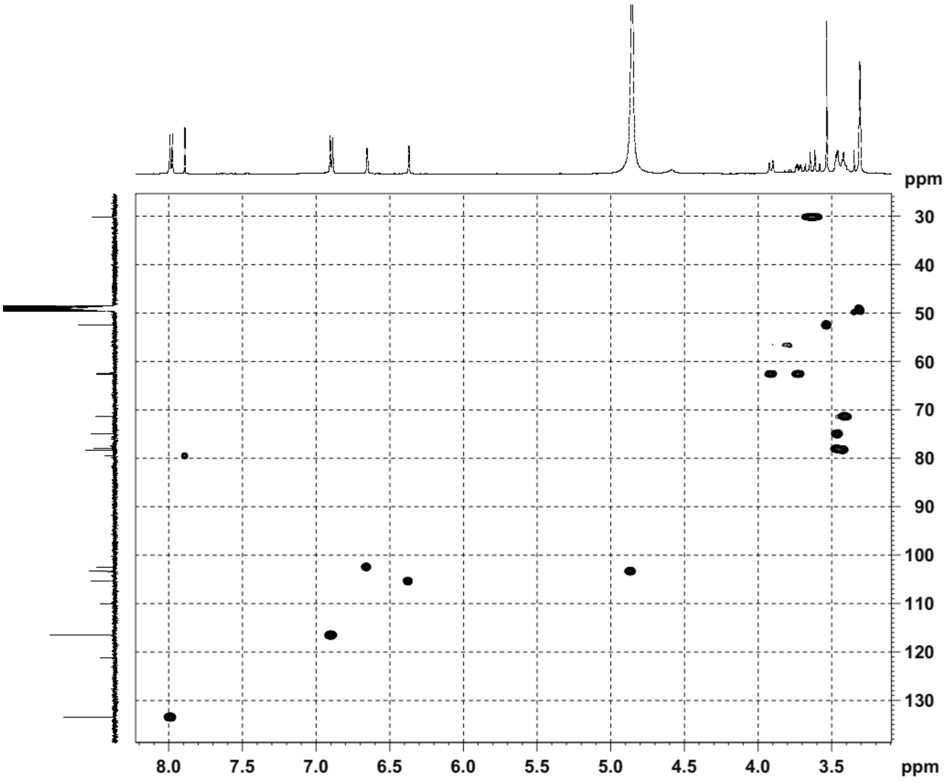


**Figure S62.** HSQC ($CD_3OD$) spectrum of **9**

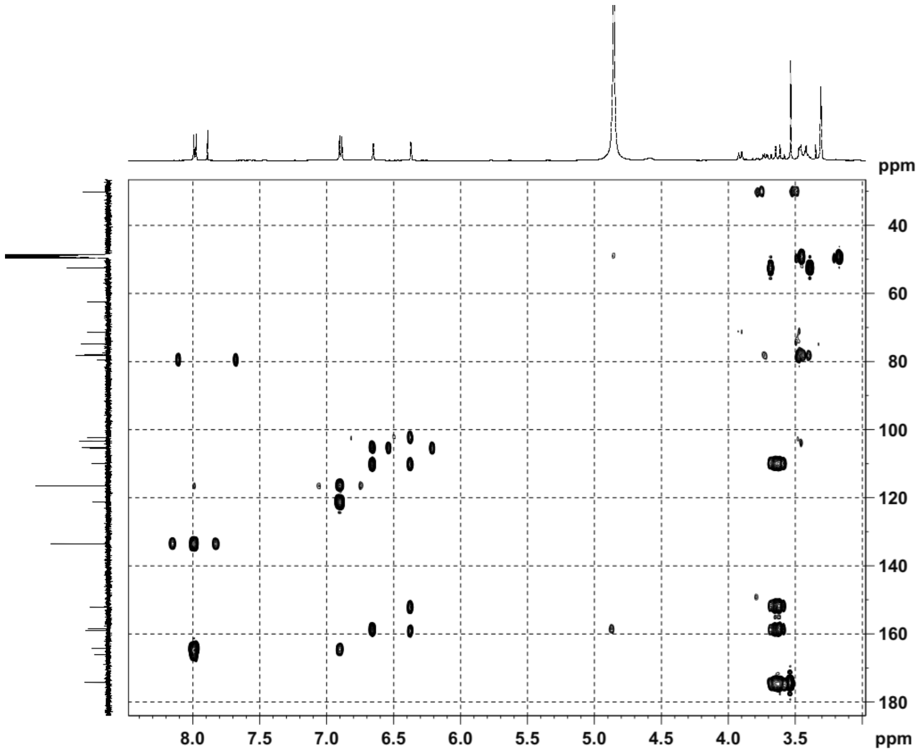


**Figure S63.** HMBC ($CD_3OD$) spectrum of **9**

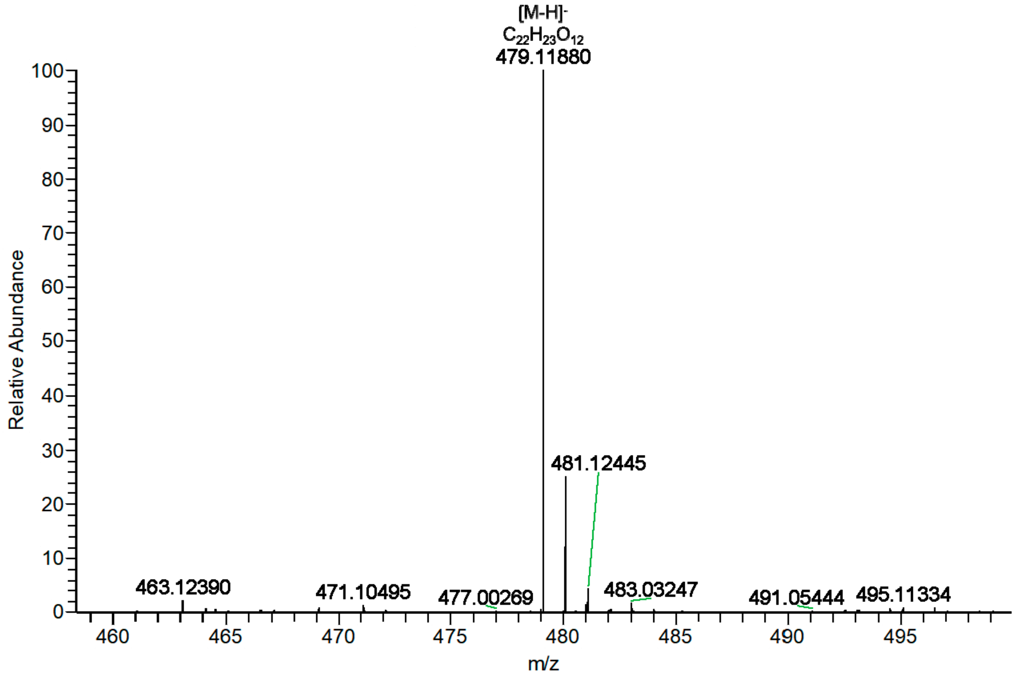


**Figure S64.** HRESIMS spectrum of **9**

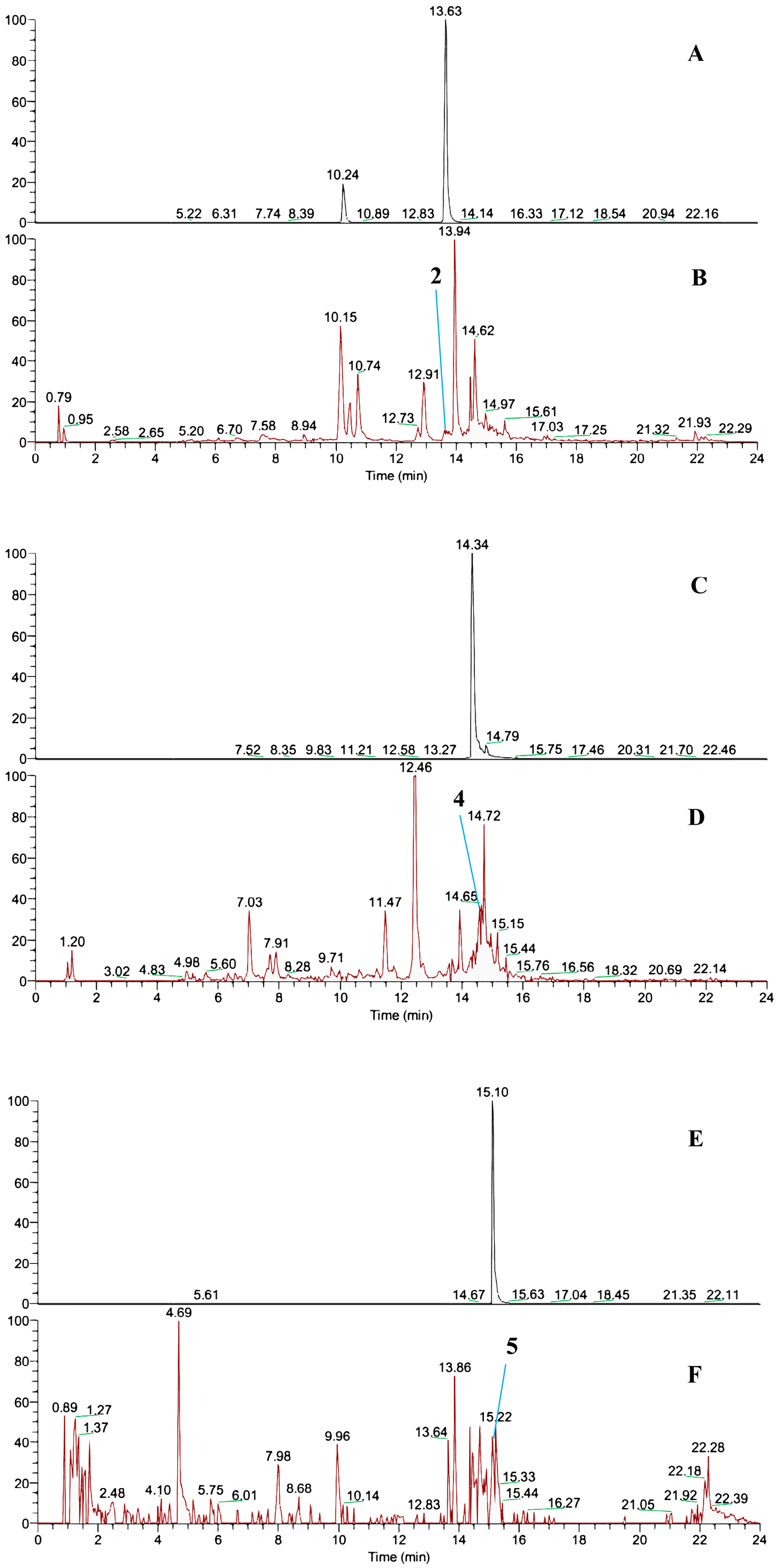
A
13.63
10.24
5.22 6.31 7.74 8.39 10.89 12.83 14.14 16.33 17.12 18.54 20.94 22.16
B
2
13.94
10.15
14.62
10.74
12.91
0.79
0.95
2.58 2.65 5.20 6.70 7.58 8.94 12.73 14.97 15.61 17.03 17.25 21.32 21.93 22.29
Time (min)
C
14.34
14.79
7.52 8.35 9.83 11.21 12.58 13.27 15.75 17.46 20.31 21.70 22.46
D
12.46
4
14.72
7.03
11.47
14.65
15.15
15.44
1.20
3.02 4.83 4.98 5.60 7.91 8.28 9.71 15.76 16.56 18.32 20.69 22.14
Time (min)
E
15.10
5.61 14.67 15.63 17.04 18.45 21.35 22.11
F
4.69
5
13.86
0.89 1.27 1.37
15.22
13.64
9.96
7.98
22.28
22.18
15.33
2.48 4.10 5.75 6.01 8.68 10.14 12.83 15.44 16.27 21.05 21.92 22.39
Time (min)

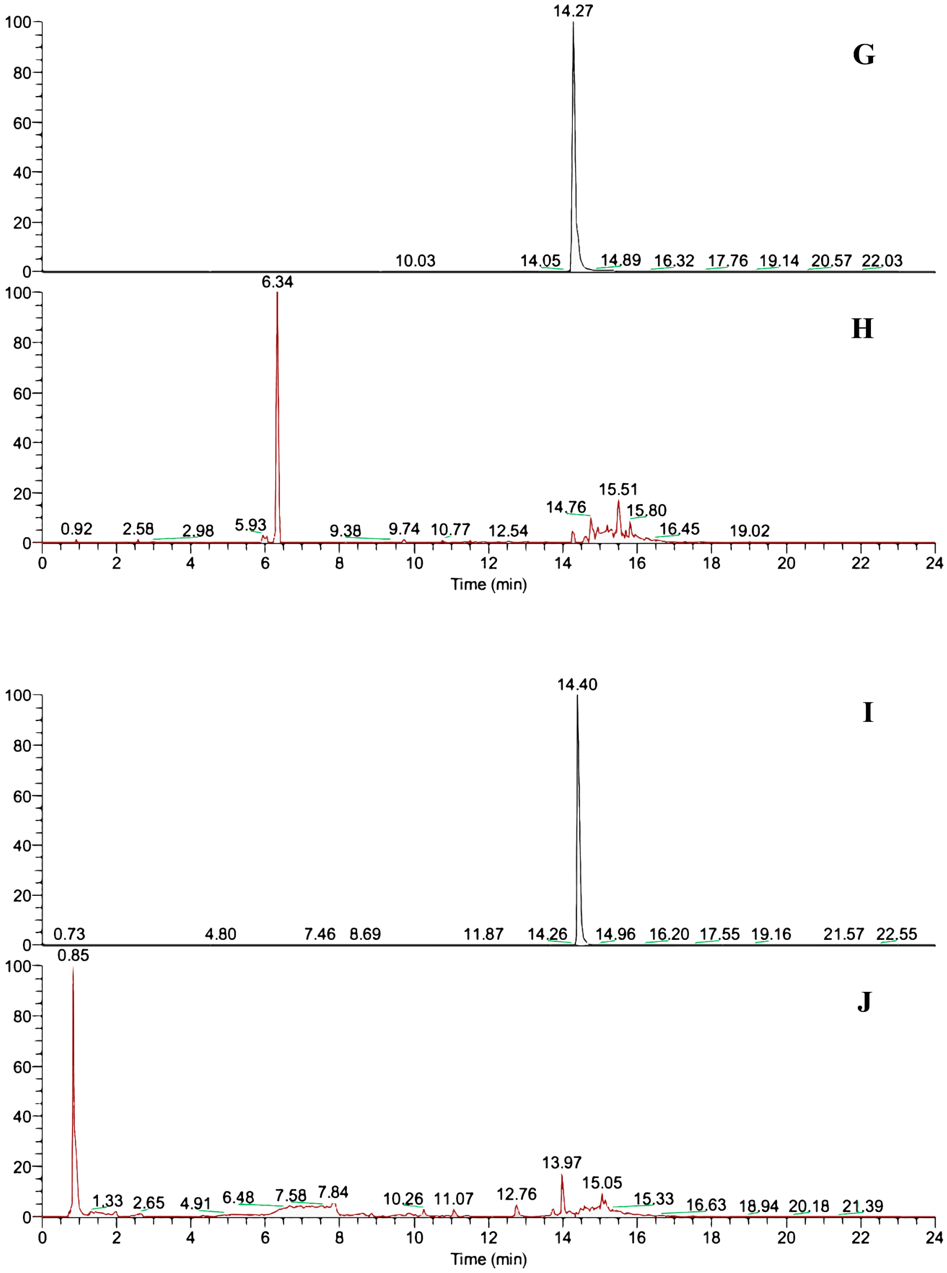


**Figure S65.** The LC-MS analysis of compounds **2**, **4**, **5**, **6** and **9** from *P. granatum* peels

A: The BPC for compound **2** (*m*/*z* 293.0305); B: The EIC of *m*/*z* 293.0305 from the 70% EtOH extract of *P. granatum* peels; C: The BPC for compound **4** (*m*/*z* 335.0400); D: The EIC of *m*/*z* 335.0400 from the 70% MeOH extract of *P. granatum* peels; E: The BPC for compound **5** (*m*/*z* 263.0561); F: The EIC of *m*/*z* 263.0561 from the 70% MeOH extract of *P. granatum* peels; G: The BPC for compound **6** (*m*/*z* 497.0388); H: The EIC of *m*/*z* 497.0388 from the 70% MeOH extract of *P. granatum* peels; I: The BPC for compound **9** (*m*/*z* 479.1211); J: The EIC of *m*/*z* 479.1211 from the 70% EtOH extract of *P. granatum* peels.

Chromatographic conditions: Column: ACQUITY UPLC CSH C18（1.7 $\mu$m, 2.1 × 100 mm）; mobile phase: A: $H_2O$; B: $CH_3CN$; gradient elution conditions: 0–2 min, 1% B, 2–3 min, 1%-5% B, 3–12 min, 5%-13% B, 12–13 min, 13%-25% B, 13–14 min, 25%-50% B, 14–22 min, 50%-100% B, 22–24 min, 100% B; column temperature: 35°C; flow rate: 0.3 mL/min; injection volume: 2 $\mu$L.

Mass spectrometry conditions: Ion source: heat electrospray ion source (HESI source); capillary voltage: 3.2 kV; capillary temperature: 350°C; ion source temperature: 320°C; sheath gas ($N_2$): 40 L/h; auxiliary gas ($N_2$): 10 L/h; normalized collision energy (NCE): 35 V; scan mode: target sim; scan range: 100–1500 *m*/*z*; detection time: 24 min; ESI acquisition mode: negative ion mode.

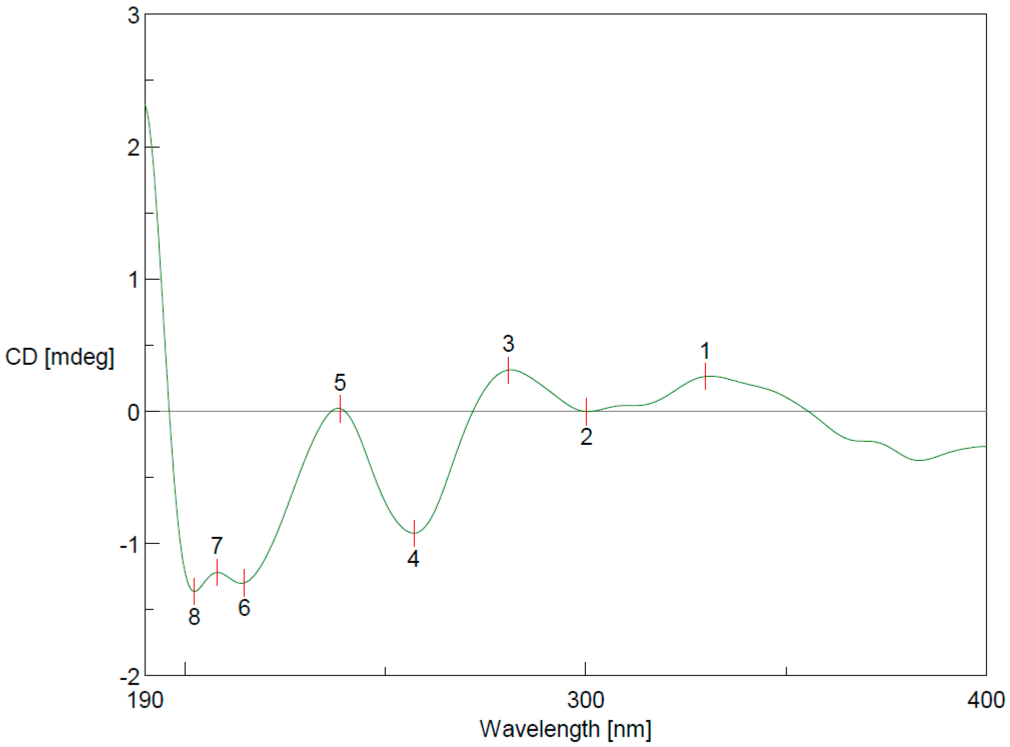


**Figure S66.** The experimental ECD spectrum of compound **14** in MeOH

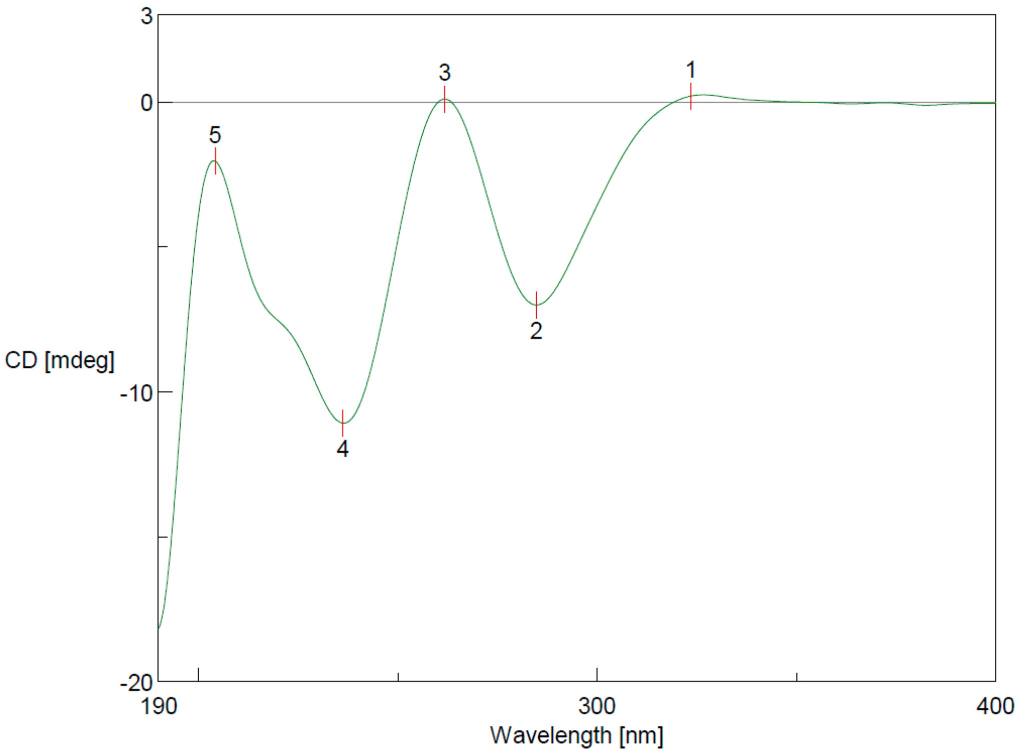


**Figure S67.** The experimental ECD spectrum of compound **16** in MeOH

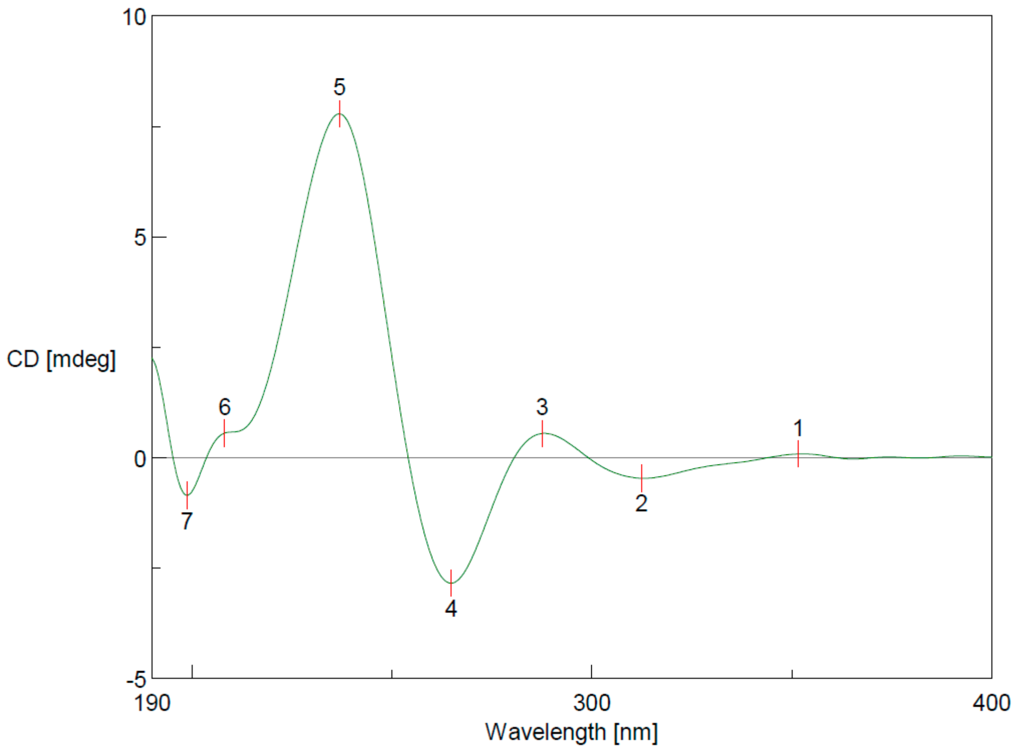


**Figure S68.** The experimental ECD spectrum of compound **17** in MeOH

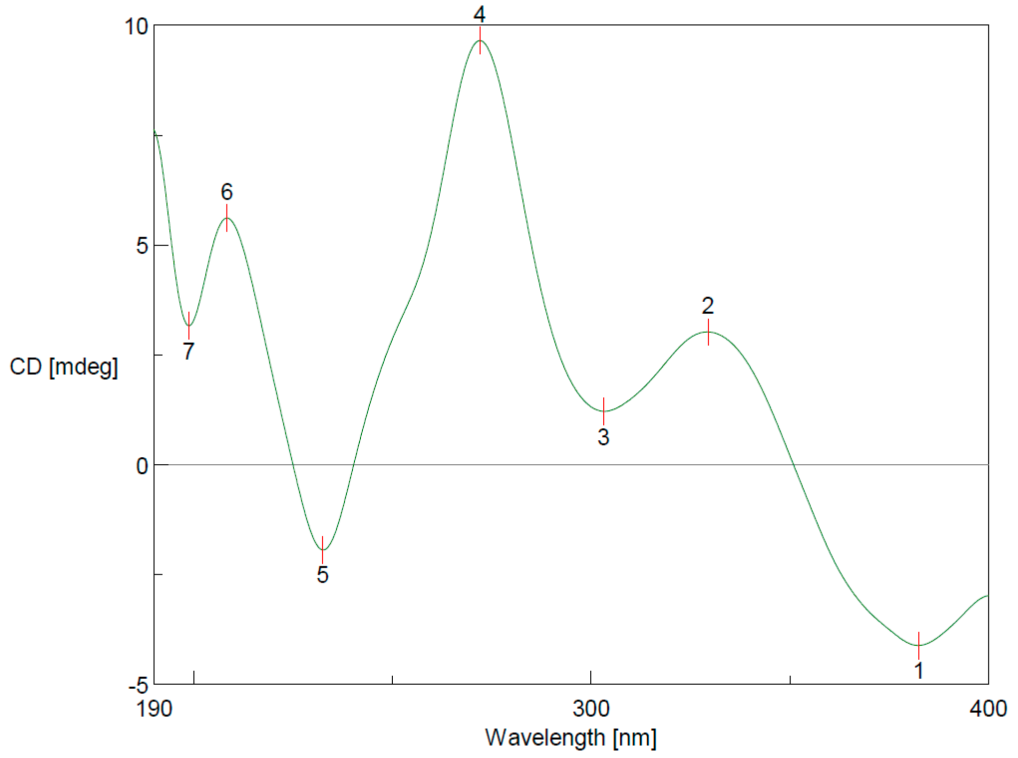


**Figure S69.** The experimental ECD spectrum of compound **18** in MeOH

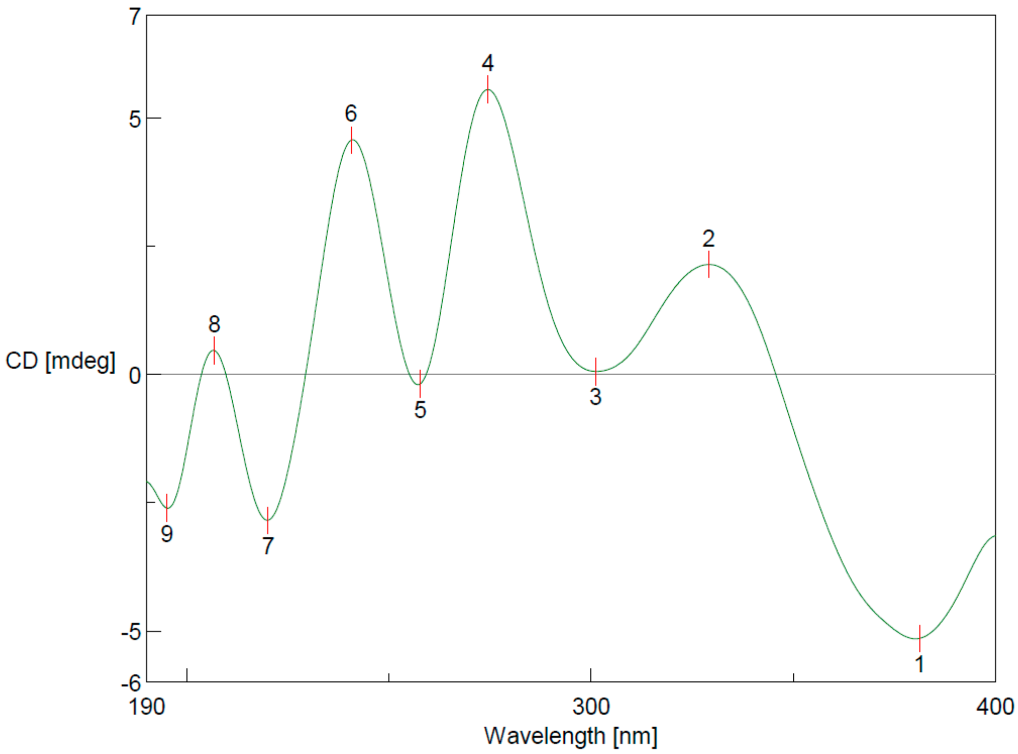


**Figure S70.** The experimental ECD spectrum of compound **19** in MeOH

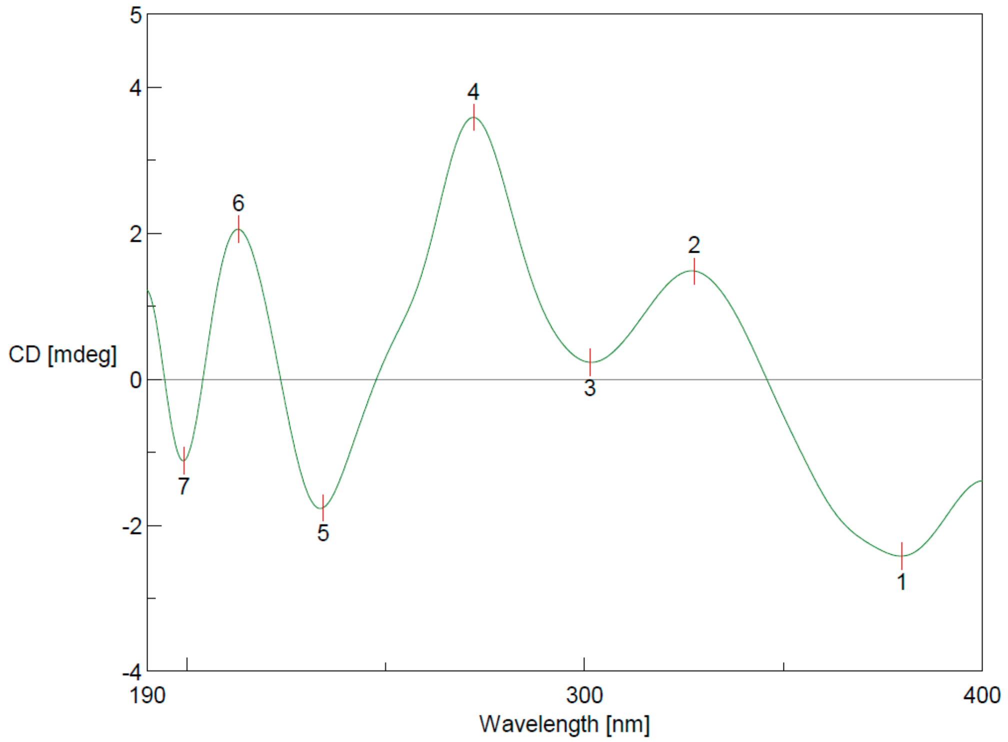


**Figure S71.** The experimental ECD spectrum of compound **20** in MeOH

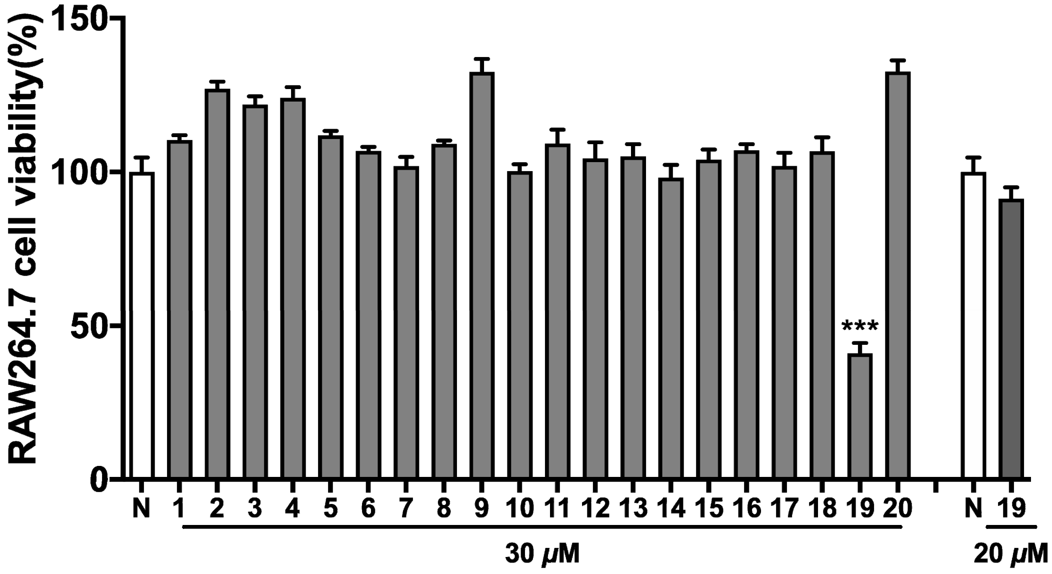


**Figure S72.** The MTT assay of compounds **1**–**18, 20** at 30 μM and **19** at 20 μM

N: normal group. Values represent the mean ± SD of six determinations. ***$P$ < 0.001 (Differences between compound-treated group and normal group).

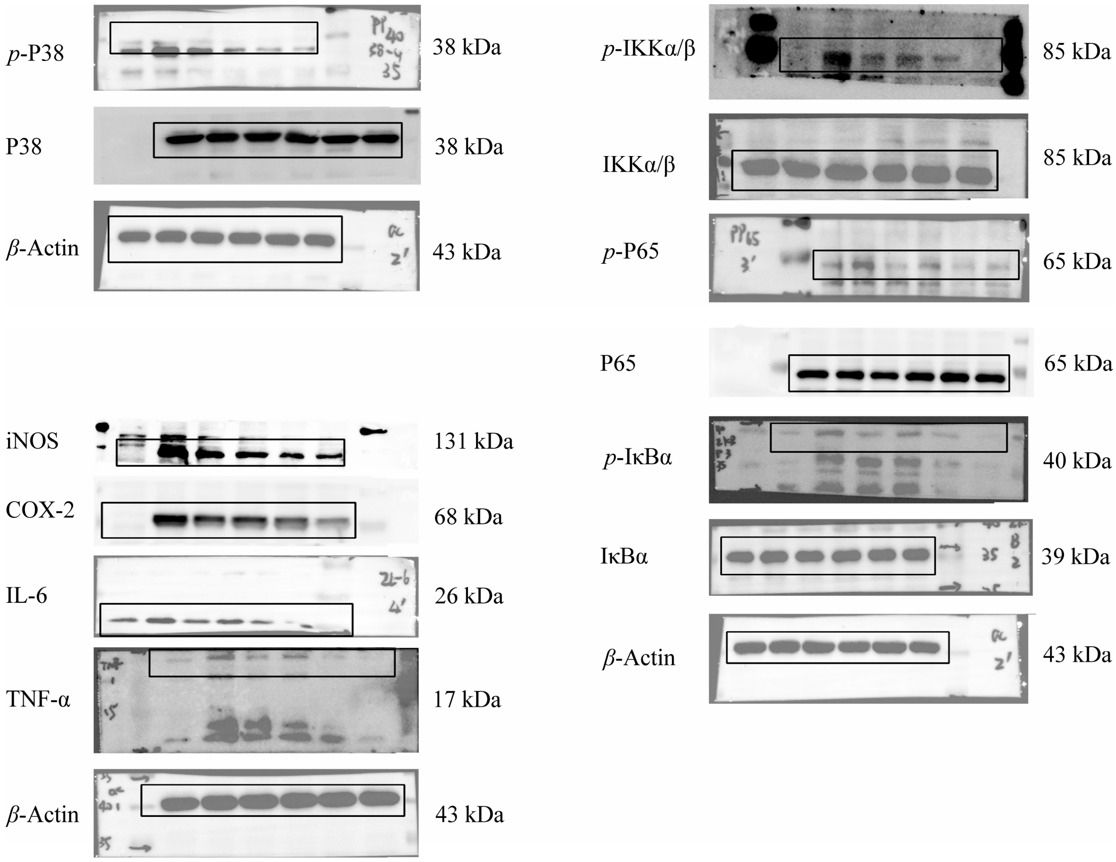


**Figure S73.** The raw data of Figure 4

**Physical and chemical data of compounds 10–20**

Brevifolin (**10**): yellow powder; $^1$H NMR (DMSO-$d_6$, 500 MHz): δ 7.29 (1H, s, H-7), 2.51 (2H, t, *J* = 4.0 Hz, H$_2$-9), 3.18 (2H, t, *J* = 4.0 Hz, H$_2$-10); $^{13}$C NMR (DMSO-$d_6$, 125 MHz): δ 141.4 (C-2), 144.9 (C-3), 115.4 (C-3a), 144.1 (C-4), 140.0 (C-5), 149.2 (C-6), 107.9 (C-7), 113.2 (C-7a), 160.5 (C-8), 23.8 (C-9), 32.9 (C-10), 195.3 (C-11); HRESIMS: *m*/*z* 247.0254 [M–H]$^-$ (calcd for $C_{12}H_7O_6$, 247.0248).

Brevifolincarboxylic acid (**11**): yellow powder; $^1$H NMR (DMSO-$d_6$, 400 MHz): δ 7.24 (1H, s, H-7), 4.37 (1H, d, *J* = 7.2 Hz, H-9), [2.63 (1H, d, *J* = 18.4 Hz), 2.83 (1H, dd, *J* = 7.2, 18.4 Hz), H$_2$-10]; $^{13}$C NMR (DMSO-$d_6$, 100 MHz): δ 141.0 (C-2), 145.3 (C-3), 115.2 (C-3a), 144.7 (C-4), 139.7 (C-5), 149.1 (C-6), 107.9 (C-7), 112.8 (C-7a), 160.4 (C-8), 41.3 (C-9), 37.3 (C-10), 193.8 (C-11), 173.1 (COOH); HRESIMS: *m*/*z* 291.0150 [M–H]$^-$ (calcd for $C_{13}H_7O_8$, 291.0146).

4-*O*-α-L-Rhamnopyranosyl ellagic acid (**12**): white powder; $^1$H NMR (DMSO-$d_6$, 500 MHz): δ 7.73 (1H, s, H-5), 7.46 (1H, br. s, H-5'), 5.47 (1H, br. s, H-1''), 4.02 (1H, br. d, ca. *J* = 3 Hz, H-2''), 3.86 (1H, dd, *J* = 3.09.0 Hz, H-3''), 3.34 (1H, dd, *J* = 9.09.0 Hz, H-4''), 3.58 (1H, m, H-5''), 1.15 (3H, d, *J* = 6.5 Hz, H$_3$-6''); $^{13}$C NMR (DMSO-$d_6$, 125 MHz): δ 114.6 (C-1), 136.4 (C-2), 142.0 (C-3), 146.4 (C-4), 111.6 (C-5), 106.7 (C-6), 159.0 (C-7), 107.0 (C-1'), 136.5 (C-2'), 140.9 (C-3'), 148.9 (C-4'), 109.8 (C-5'), 111.9 (C-6'), 159.2 (C-7'), 100.0 (C-1''), 69.8 (C-2''), 70.0 (C-3''), 71.7 (C-4''), 69.8 (C-5''), 17.8 (C-6''); HRESIMS: *m*/*z* 447.0576 [M–H]$^-$ (calcd for $C_{20}H_{15}O_{12}$, 447.0569).

4-*O*-β-D-Glucopyranosyl-3,3'-di-*O*-methylellagic acid (**13**): white powder; $^1$H NMR ($C_5D_5N$, 500 MHz): δ 8.45 (1H, s, H-5), 8.05 (1H, s, H-5'), 5.89 (1H, d, *J* = 7.5 Hz, H-1''), 4.41 (1H, dd, *J* = 9.0, 9.0 Hz, H-2''), 4.41 (1H, dd, *J* = 9.0, 9.0 Hz, H-3''), 4.38 (1H, dd, *J* = 9.0, 9.0 Hz, H-4''), 4.18 (1H, m, H-5''), [4.42 (1H, m), 4.60 (1H, dd, *J* = 2.0, 12.0 Hz), H$_2$-6''], 4.27 (3H, s, 3-OCH$_3$), 4.20 (3H, s, 3'-OCH$_3$); $^{13}$C NMR ($C_5D_5N$, 125 MHz): δ 114.9 (C-1), 142.4 (C-2), 142.9 (C-3), 152.6 (C-4), 113.2 (C-5), 112.9 (C-6), 159.2 (C-7), 111.7 (C-1'), 141.9 (C-2'), 142.9 (C-3'), 154.5 (C-4'), 113.1 (C-5'), 113.9 (C-6'), 159.1 (C-7'), 103.0 (C-1''), 74.9 (C-2''), 78.5 (C-3''), 71.1 (C-4''), 79.2 (C-5''), 62.4 (C-6''), 62.0 (3-OCH$_3$), 61.4 (3'-OCH$_3$); HRESIMS: *m*/*z* 491.0845 [M–H]$^-$ (calcd for $C_{22}H_{19}O_{13}$, 491.0831).

(*S*)-flavogallonic acid (**14**): white powder; $[\alpha]_D^{25}$ –10.0 (*c* 0.06, MeOH); CD (*c* 0.00021 M, MeOH) mdeg (*λ*nm): +0.02 (238), –0.92 (257), +0.31 (281), –0.00 (300), +0.26 (329); $^1$H NMR ($CD_3OD$, 500 MHz): δ 7.40 (1H, br. s, H-5), 7.31 (1H, s, H-6''); $^{13}$C-NMR ($CD_3OD$, 125 MHz): δ 114.5 (C-1), 137.6 (C-2)$^a$, 140.4 (C-3), 149.0 (C-4), 111.3 (C-5), 108.9 (C-6)$^b$, 161.6 (C-7), 114.1 (C-1'), 136.9 (C-2')$^a$, 140.1 (C-3'), 147.3 (C-4'), 109.2 (C-5')$^b$, 121.4 (C-6'), 160.2 (C-7'), 126.0 (C-1''), 119.1 (C-2''), 144.4 (C-3''), 139.1 (C-4''), 145.4 (C-5''), 111.8 (C-6''), 170.4 (C-7'') (the data of $^a$C-2 and C-2', $^b$C-6 and C-5' maybe exchanged); HRESIMS: *m*/*z* 469.0062 [M–H]$^-$ (calcd for $C_{21}H_9O_{13}$, 469.0049).

Valoneic acid dilactone (**15**): white powder; $^1$H NMR (DMSO-$d_6$, 500 MHz): δ 7.54 (1H, s, H-5), 7.01 (1H, s, H-5'), 7.04 (1H, s, H-6''); $^{13}$C NMR (DMSO-$d_6$, 125 MHz): δ 111.9 (C-1), 139.4 (C-2)$^a$, 139.6 (C-3), 148.5 (C-4), 110.3 (C-5), 108.0 (C-6), 159.2 (C-7), 113.9 (C-1'), 136.2 (C-2')$^a$, 141.1 (C-3'), 149.4 (C-4'), 108.6 (C-5'), 106.4 (C-6'), 159.1 (C-7'), 114.6 (C-1''), 139.1 (C-2''), 136.5 (C-3'')$^a$, 135.2 (C-4''), 142.8 (C-5''), 108.2 (C-6''), 166.0 (C-7'') ($^a$the data of C-2 and C-3'' maybe exchanged); HRESIMS: *m*/*z* 469.0062 [M–H]$^-$ (calcd for $C_{21}H_9O_{13}$, 469.0049).

Corilagin (**16**): white powder; CD (*c* 0.00016 M, MeOH) mdeg (*λ*nm): –11.1 (236), +0.09 (262), –7.01 (285), +0.19 (324); $^1$H NMR ($CD_3OD$, 500 MHz): δ 6.37 (1H, br. s, H-1), 3.99 (1H, br. s, H-2), 4.81 (1H, br. s, H-3), 4.47 (1H, br. s, H-4), 4.52 (1H, dd, *J* = 8.5, 11.0 Hz, H-5), [4.15 (1H, dd, *J* = 8.5, 11.0 Hz), 4.98 (1H, dd, *J* = 11.0, 11.0 Hz), H$_2$-6], 7.05 (2H, s, H-2',6'), 6.69 (1H, s, H-5''), 6.66 (1H, s, H-5'''); $^{13}$C NMR ($CD_3OD$, 125 MHz): δ 95.0 (C-1), 69.3 (C-2), 71.3 (C-3), 62.4 (C-4), 76.1 (C-5), 65.2 (C-6), 120.6 (C-1'), 111.0 (C-2',6'), 146.3 (C-3',5'), 140.4 (C-4'), 166.6 (C-7'), 117.1 (C-1''), 145.2 (C-2''), 138.2 (C-3''), 145.6 (C-4''), 110.2 (C-5''), 125.4 (C-6''), 168.5 (C-7''), 116.6 (C-1'''), 145.1 (C-2'''), 137.6 (C-3'''), 145.9 (C-4'''), 108.3 (C-5'''), 125.5 (C-6'''), 170.1 (C-7'''); HRESIMS: *m*/*z* 633.0704 [M–H]$^-$ (calcd for $C_{27}H_{21}O_{18}$, 633.0733).

2,3-(*S*)-Hexahydroxydiphenoyl-D-glucose (**17**) (1α:1β ≈ 7:8): white powder; CD (*c* 0.00021 M, MeOH) mdeg (*λ*nm): +7.78 (237), –2.84 (265), +0.55 (288), –0.47 (312), +0.08 (352); $^1$H NMR (Acetone-$d_6$:$D_2O$, 9:1, 400 MHz): δ 5.42 (1H, d, *J* = 3.6 Hz, Glc-H-1α), 4.93 (1H, dd, *J* = 3.6, 9.6 Hz, Glc-H-2α), 5.36 (1H, dd, *J* = 9.6, 9.6 Hz, Glc-H-3α), 3.80 (1H, dd, *J* = 9.6, 9.6 Hz, Glc-H-4α), 3.95 (1H, m, Glc-H-5α), 3.82–3.96 (2H, m, Glc-H$_2$-6α), 6.67 (1H, s, H-5'α), 6.77 (1H, s, H-5''α); 5.00 (1H, d, *J* = 8.0 Hz,

Glc-H-1β), 4.73 (1H, dd, *J* = 8.0, 9.6 Hz, Glc-H-2β), 5.03 (1H, dd, *J* = 9.6, 9.6 Hz, Glc-H-3β), 3.76 (1H, dd, *J* = 9.6, 9.6 Hz, Glc-H-4β), 3.56 (1H, m, Glc-H-5β), 3.82–3.96 (2H, m, Glc-$H_2$-6β), 6.67 (1H, s, H-5'β), 6.75 (1H, s, H-5''β); $^{13}$C NMR (Acetone-$d_6$:$D_2O$, 9:1, 100 MHz): δ 91.2 (Glc-C-1α), 75.5 (Glc-C-2α), 78.2 (Glc-C-3α), 68.2 (Glc-C-4α), 72.9 (Glc-C-5α), 61.7 (Glc-C-6α), 114.7 (C-1'α), 144.3 (C-2'α)[a], 136.2 (C-3'α), 145.2 (C-4'α), 107.4 (C-5'α), 126.6 (C-6'α)[b], 169.7 (C-7'α), 114.5 (C-1''α), 144.3 (C-2''α)[a], 136.1 (C-3''α), 145.2 (C-4''α), 107.6 (C-5''α), 126.6 (C-6''α)[b], 170.2 (C-7''α) (the data of [a]C-2'α and C-2''α; [b]C-6'α and C-6''α maybe exchanged); 94.5 (Glc-C-1β), 77.8 (Glc-C-2β), 80.5 (Glc-C-3β), 67.9 (Glc-C-4β), 77.7 (Glc-C-5β), 61.7 (Glc-C-6β), 114.7 (C-1'β), 144.2 (C-2'β)[a], 136.3 (C-3'β), 145.2 (C-4'β), 107.4 (C-5'β), 126.9 (C-6'β)[b], 169.6 (C-7'β), 114.6 (C-1''β), 144.4 (C-2''β)[a], 136.1 (C-3''β), 145.2 (C-4''β), 107.6 (C-5''β), 127.1 (C-6''β)[b], 170.2 (C-7''β) (the data of [a]C-2'α and C-2''α; [b]C-6'α and C-6''α maybe exchanged); HRESIMS: *m/z* 481.0624 [M–H]$^-$ (calcd for $C_{20}H_{17}O_{14}$, 481.0624).

Punicalin (**18**) (1α:1β ≈ 1:1): pale yellow powder; CD (*c* 0.00013 M, MeOH) mdeg (*λ*nm): –1.93 (233), +9.64 (272), +1.21 (303), +3.02 (329), –4.11 (383); $^1$H NMR (Acetone-$d_6$:$D_2O$, 9:1, 400 MHz): δ 4.87 (1H, d, *J* = 2.8 Hz, Glc-H-1α), 3.36 (1H, dd, *J* = 2.8, 7.6 Hz, Glc-H-2α), 3.81 (1H, dd, *J* = 7.6, 7.6 Hz, Glc-H-3α), 4.26 (1H, dd, *J* = 7.6, 7.6 Hz, Glc-H-4α), 2.87 (1H, m, Glc-H-5α), [2.59 (1H, br. d, *ca. J* = 11 Hz), 4.08 (1H, dd, *J* = 11.2, 11.2 Hz), Glc-$H_2$-6α], 6.76 (1H, s, gallagyl-H-4α), 7.07 (1H, s, gallagyl-H-6α); 4.34 (1H, d, *J* = 8.8 Hz, Glc-H-1β), 3.21 (1H, dd, *J* = 8.8, 8.8 Hz, Glc-H-2β), 3.47 (1H, dd, *J* = 8.8, 8.8 Hz, Glc-H-3β), 4.39 (1H, dd, *J* = 8.8, 8.8 Hz, Glc-H-4β), 2.46 (1H, m, Glc-H-5β), [2.35 (1H, br. d, *ca. J* = 11 Hz), 3.97 (1H, dd, *J* = 10.8, 10.8 Hz), Glc-$H_2$-6β], 6.75 (1H, s, gallagyl-H-4β), 7.03 (1H, s, gallagyl-H-6β); $^{13}$C NMR (Acetone-$d_6$:$D_2O$, 9:1, 100 MHz): δ 90.0 (Glc-C-1α), 71.8 (Glc-C-2α), 70.1 (Glc-C-3α), 73.4 (Glc-C-4α), 70.8 (Glc-C-5α), 64.1 (Glc-C-6α); 96.9 (Glc-C-1β), 75.1 (Glc-C-2β), 75.5 (Glc-C-3β), 74.0 (Glc-C-4β), 72.7 (Glc-C-5β), 65.2 (Glc-C-6β); gallagyl part: 109.8 (gallagyl-C-4α), 110.0, 110.0, 110.1 (gallagyl-C-4β), 110.2, 110.4, 111.69 (gallagyl-C-6β), 111.74 (gallagyl-C-6α), 114.2, 114.4, 114.6, 114.9, 115.0, 115.2, 117.9, 118.1, 122.3, 122.6, 122.8, 123.2, 125.0, 125.0, 125.1, 125.3, 136.3, 136.4, 136.59, 136.63, 137.1, 137.4, 137.9, 137.9, 139.1, 139.2, 140.3, 140.4, 144.1, 144.2, 144.5, 144.5, 145.4, 145.4, 145.5, 145.6, 147.6, 147.8, 147.9, 148.2, 159.2 (lactone carbonyl-C), 159.3 (lactone carbonyl-C), 159.7 (lactone carbonyl-C), 160.1 (lactone carbonyl-C), 168.8 (ester carbonyl-C-6β), 168.8 (ester carbonyl-C-6α), 170.0 (ester carbonyl-C-4a), 170.0 (ester carbonyl-C-4β).

$^1$H NMR ($CD_3OD$, 400 MHz): δ 4.84 (1H, d, *J* = 2.8 Hz, Glc-H-1α), 3.26 (1H, dd, *J* = 2.8, 8.4 Hz, Glc-H-2α), 3.65 (1H, dd, *J* = 8.0, 8.4 Hz, Glc-H-3α), 4.25 (1H, dd, *J* = 8.0, 8.0 Hz, Glc-H-4α), 2.95 (1H, m, Glc-H-5α), [2.31 (1H, br. d, ca. *J* = 11 Hz), 3.99 (1H, dd, *J* = 10.8, 10.8 Hz), Glc-$H_2$-6α], 6.69 (1H, s, gallagyl-H-4α), 6.93 (1H, s, gallagyl-H-6α); 4.23 (1H, d, *J* = 7.6 Hz, Glc-H-1β), 3.06 (1H, dd, *J* = 7.6, 8.8 Hz, Glc-H-2β), 3.31 (1H, dd, *J* = 8.8, 8.8 Hz, Glc-H-3β), 4.36 (1H, dd, *J* = 8.8, 8.8 Hz, Glc-H-4β), 2.44 (1H, m, Glc-H-5β), [2.20 (1H, br. d, ca. *J* = 11 Hz), 3.92 (1H, dd, *J* = 10.8, 10.8 Hz), Glc-$H_2$-6β], 6.69 (1H, s, gallagyl-H-4β), 6.91 (1H, s, gallagyl-H-6β); $^{13}$C NMR ($CD_3OD$, 100 MHz) δ: 91.7 (Glc-C-1α), 72.9 (Glc-C-2α), 69.2 (Glc-C-3α), 74.4 (Glc-C-4α), 72.5 (Glc-C-5α), 64.9 (Glc-C-6α), 97.7 (Glc-C-1β), 75.9 (Glc-C-2β), 76.7 (Glc-C-3β), 74.4 (Glc-C-4β), 73.1 (Glc-C-5β), 65.4 (Glc-C-6β); gallagyl part: δ 110.1 (gallagyl-C-4α), 110.3 (gallagyl-C-4β), 110.5, 110.5, 110.8, 110.9, 111.9 (gallagyl-C-6β), 111.9 (gallagyl-C-6α), 114.6, 114.7, 115.2, 115.3, 115.8, 115.9, 118.3, 118.6, 122.7, 1228, 123.5, 123.8, 125.2, 125.3, 125.6, 125.7, 137.0, 137.0, 137.1, 137.2, 137.5, 137.6, 138.7, 138.7, 139.9, 139.9, 141.0, 141.2, 144.4, 144.4, 144.9, 144.9, 146.0, 146.0, 146.1, 146.3, 148.4, 148.6, 148.7, 148.8, 160.2 (lactone carbonyl-C), 160.3 (lactone carbonyl-C), 160.4 (lactone carbonyl-C), 160.5 (lactone carbonyl-C), 169.7 (ester carbonyl-C-6β), 169.9 (ester carbonyl-C-6α), 171.4 (ester carbonyl-C-4α), 171.4 (ester carbonyl-C-4β); HRESIMS: *m/z* 781.0566 [M–H]$^-$ (calcd for $C_{34}H_{21}O_{22}$, 781.0530).

Punicalagin (**19**) (1α:1β ≈ 1:1): pale yellow powder; CD (*c* 0.00010 M, MeOH) mdeg (*λ*nm): +4.56 (240), –0.19 (257), +5.55 (275), +0.06 (301), +2.14 (329), –5.14 (381); $^1$H-NMR (Acetone-$d_6$:$D_2O$, 9:1, 400 MHz): δ 5.12 (1H, d, *J* = 3.2 Hz, Glc-H-1α), 4.81 (1H, dd, *J* = 3.2, 9.6 Hz, Glc-H-2α), 5.23 (1H, dd, *J* = 9.6, 9.6 Hz, Glc-H-3α), 4.82 (1H, dd, *J* = 9.6, 9.6 Hz, Glc-H-4α), 3.27 (1H, m, Glc-H-5α), [2.17 (1H, br. d, ca. *J* = 11 Hz), 4.07 (1H, br. d, *ca. J* = 11 Hz), Glc-$H_2$-6α], 6.57 (1H, s, HHDP-H-2α), 6.66 (1H, s, HHDP-H-3α), 6.73 (1H, s, gallagyl-H-4α), 7.01 (1H, s, gallagyl-H-6α); 4.68 (1H, d, *J* = 10.8 Hz, Glc-H-1β), 4.69 (1H, dd, *J* = 9.6, 10.8 Hz, Glc-H-2β), 4.92 (1H, dd, *J* = 9.6, 9.6 Hz, Glc-H-3β), 4.84 (1H, dd, *J* = 9.6, 9.6 Hz,

Glc-H-4β), 2.66 (1H, m, Glc-H-5β), [2.24 (1H, br. d, ca. *J* = 11 Hz), 4.12 (1H, br. d, ca. *J* = 11 Hz), Glc-$H_2$-6β], 6.58 (1H, s, HHDP-H-2β), 6.66 (1H, s, HHDP-H-3β), 6.80 (1H, s, gallagyl-H-4β), 7.02 (1H, s, gallagyl-H-6β); $^{13}$C NMR (Acetone-$d_6$:$D_2O$, 9:1, 100 MHz): δ 90.0 (Glc-C-1α), 74.4 (Glc-C-2α), 76.8 (Glc-C-3α), 71.0 (Glc-C-4α), 66.6 (Glc-C-5α), 64.4 (Glc-C-6α); 94.3 (Glc-C-1β), 76.7 (Glc-C-2β), 79.1 (Glc-C-3β), 70.8 (Glc-C-4β), 72.6 (Glc-C-5β), 64.3 (Glc-C-6β); HHDP and gallagyl parts: δ 107.3 (HHDP-C-2β), 107.5 (2C, HHDP-C-2α, 3β), 107.6 (HHDP-C-3α), 109.4 (gallagyl-C-4α), 109.9 (gallagyl-C-4β), 110.2, 110.3, 111.7 (gallagyl-C-6α), 111.8 (gallagyl-C-6β), 114.0, 114.1, 114.6, 114.6, 114.9, 115.0, 115.2, 115.3, 118.0, 118.2, 122.0, 122.0, 123.4, 123.6, 124.5, 124.7, 124.9, 125.1, 126.0, 126.3 (2C), 126.4, 136.3 (2C), 136.5, 136.6 (2C), 136.9, 136.9, 137.2, 137.9, 138.0, 138.9, 139.0, 140.1, 140.6, 144.0, 144.1, 144.3, 144.4, 144.4, 144.4, 144.7, 144.7, 145.1, 145.2, 145.2, 145.2, 145.3, 145.4, 145.4, 145.5, 145.7, 145.7, 147.8, 147.9, 148.2, 148.4, 158.8 (lactone carbonyl-C), 158.8 (lactone carbonyl-C), 159.2 (lactone carbonyl-C), 159.3 (lactone carbonyl-C), 168.6 (ester carbonyl-C-6β), 168.8 (ester carbonyl-C-6α), 168.9 (ester carbonyl-C-3β), 169.0 (ester carbonyl-C-3α), 169.1 (ester carbonyl-C-2β), 169.4 (ester carbonyl-C-2α), 169.7 (ester carbonyl-C-4β), 170.0 (ester carbonyl-C-4α); HRESIMS: *m/z*1083.0555 $[M–H]^-$ (calcd for $C_{48}H_{27}O_{30}$, 1083.0593).

Punicacortein C (**20**): pale yellow powder; CD (*c* 0.00010 M, MeOH) mdeg (*λ*nm): –1.76 (234), +3.58 (272), +0.23 (301), +1.48 (328), –2.42 (379); $[\alpha]_D^{25}$ –40.4 (*c* 0.58, MeOH). $^1$H NMR ($CD_3OD$, 400 MHz): δ 5.34 (1H, d, *J* = 4.8 Hz, Glc-H-1), 4.87 (1H, dd, *J* = 2.0, 4.8 Hz, Glc-H-2), 5.07 (1H, dd, *J* = 2.0, 5.2 Hz, Glc-H-3), 4.22 (1H, dd, *J* = 1.2, 5.2 Hz, Glc-H-4), 2.26 (1H, dd, *J* = 1.2, 10.0 Hz, Glc-H-5), [3.64 (1H, d, *J* = 10.0 Hz), 3.83 (1H, dd, *J* = 10.0, 10.0 Hz), Glc-$H_2$-6], 6.29 (1H, s, HHDP-H-3), 6.70 (1H, s, gallagyl-H-4), 7.26 (1H, s, gallagyl-H-6); $^{13}$C NMR ($CD_3OD$, 100 MHz): δ 68.7 (Glc-C-1), 75.3 (Glc-C-2), 68.2 (Glc-C-3), 74.2 (Glc-C-4), 73.2 (Glc-C-5), 68.5 (Glc-C-6); HHDP and gallagyl parts: 105.0 (HHDP-C-3), 109.5 (gallagyl-C-4), 110.5, 113.0 (gallagyl-C-6), 115.1, 115.7, 116.2, 116.5, 116.9, 119.0, 120.2, 120.5, 123.0, 125.0, 125.2, 127.2, 135.8, 137.2, 137.4, 138.6, 139.5, 139.6, 140.8, 140.9, 144.6, 144.7, 144.8, 145.3, 145.8, 146.2, 146.5, 146.9, 147.7, 148.6, 160.4 (lactone carbonyl-C), 161.4 (lactone carbonyl-C), 167.5 (ester carbonyl-C-2), 168.9 (ester carbonyl-C-6), 169.6 (ester carbonyl-C-4), 170.0 (ester carbonyl-C-3); HRESIMS: *m/z* 1083.0636 $[M–H]^-$ (calcd for $C_{48}H_{27}O_{30}$, 1083.0593).
